# Kinesin-like protein KIFC2 stabilizes CDK4 to accelerate growth and confer resistance in HR^+^/HER2^–^ breast cancer

**DOI:** 10.1172/JCI183531

**Published:** 2025-04-29

**Authors:** Shao-Ying Yang, Ming-Liang Jin, Lisa Andriani, Qian Zhao, Yun-Xiao Ling, Cai-Jin Lin, Min-Ying Huang, Jia-Yang Cai, Yin-Ling Zhang, Xin Hu, Zhi-Ming Shao, Fang-Lin Zhang, Xi Jin, A Yong Cao, Da-Qiang Li

**Affiliations:** 1 Cancer Institute, Fudan University Shanghai Cancer Center, Shanghai Medical College, Fudan University, Shanghai, China.; 2 Department of Oncology, Shanghai Medical College, Fudan University, Shanghai, China.; 3 Department of Breast Surgery, Fudan University Shanghai Cancer Center, Fudan University, Shanghai, China.; 4 Institutes of Biomedical Sciences, Shanghai Medical College, Fudan University, Shanghai, China.; 5 Precision Cancer Medicine Center, Fudan University Shanghai Cancer Center, Shanghai, China.; 6 Shanghai Key Laboratory of Breast Cancer, Shanghai Medical College, Fudan University, Shanghai, China

**Keywords:** Cell biology, Oncology, Therapeutics, Breast cancer, Drug therapy, Molecular biology

## Abstract

Hormone receptor–positive and human epidermal growth factor receptor 2–negative breast cancer (HR^+^/HER2^−^ BC) is the most common subtype, with a high risk of long-term recurrence and metastasis. Endocrine therapy (ET) combined with cyclin-dependent kinase 4/6 (CDK4/6) inhibitors is a standard treatment for advanced/metastatic HR^+^/HER2^–^ BC, but resistance remains a major clinical challenge. We report that kinesin family member C2 (*KIFC2*) was amplified in approximately 50% of patients with HR^+^/HER2^–^ BC, and its high expression was associated with poor disease outcome, increased tumor protein p53 (*TP53*) somatic mutation, and active pyrimidine metabolism. Functional assays revealed that depletion of KIFC2 suppressed growth and enhanced sensitivity of HR^+^/HER2^–^ BC cells to tamoxifen and CDK4/6 inhibitors. Mechanistically, KIFC2 stabilized CDK4 by enhancing its interaction with ubiquitin-specific peptidase 9 X-linked (USP9X). Importantly, reexpression of CDK4 in KIFC2-depleted cells partially rescued the decreased growth and increased sensitivity to tamoxifen and CDK4/6 inhibitors caused by KIFC2 depletion. Clinically, high *KIFC2* mRNA expression was negatively associated with the survival rate of patients with HR^+^/HER2^–^ BC who received adjuvant ET alone or in combination with CDK4/6 inhibitors. Collectively, these findings identify an important role for KIFC2 in HR^+^/HER2^–^ BC growth and therapeutic resistance, and support its potential as a therapeutic target and predictive biomarker.

## Abstract 2

KIFC2 stabilizes CDK4 to accelerate growth and confer resistance to endocrine therapy and CDK4/6 inhibitors in HR+/HER2- breast cancer

## Graphical abstract

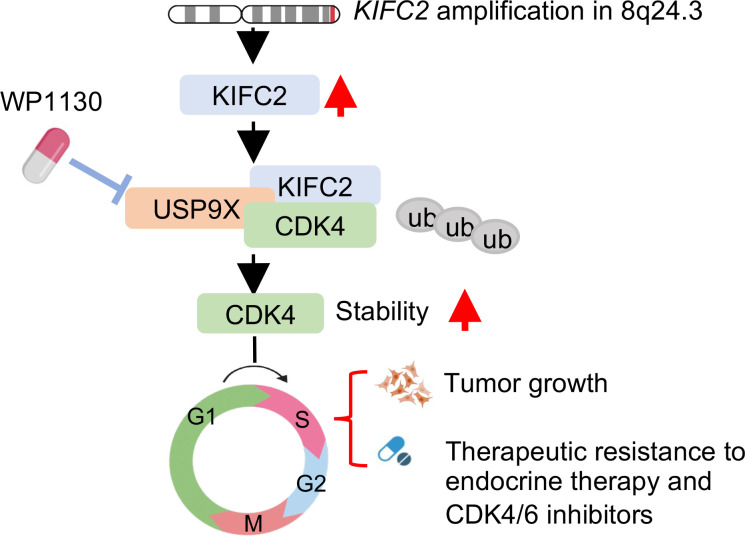

## Introduction

Breast cancer (BC) is the most common malignancy in women worldwide, with a high level of clinical and biological heterogeneity. Clinically, BC is divided into 3 primary molecular subtypes based on the expression status of estrogen and progesterone receptors (collectively referred to as hormone receptor [HR]) and human epidermal growth factor receptor 2 (HER2). The HR^+^/HER2^–^ subtype accounts for approximately two-thirds of all primary BC cases (1). Of note, approximately 10%–30% of patients with early-stage HR^+^/HER2^–^ BC experience local recurrence or distant metastasis after surgery that develops into advanced disease (1).

Estrogen-estrogen receptor (E2-ER) signaling is a key driver of HR^+^/HER2^–^ BC progression (2). Cyclin D, a major transcriptional target of E2-ER signaling, forms a complex with cyclin-dependent kinase 4/6 (CDK4/6) to phosphorylate retinoblastoma protein (RB), thereby activating E2F–mediated transcription and promoting G_1_- to S-phase transition (3). Endocrine therapy (ET) — including tamoxifen (Tam), fulvestrant, and aromatase inhibitors — targets this pathway and is widely used in HR^+^/HER2^−^ BC (1, 4). However, approximately 20% of early-stage and nearly all patients with metastases eventually develop resistance to ET (5).

Notably, emerging evidence shows that HR^+^/HER2^–^ BC cells resistant to ET rely on CDK4 to drive cell proliferation (3, 6). Thus, combination of CDK4/6 inhibitors with ET has the potential to overcome resistance to ET (7). Indeed, multiple clinical trials have demonstrated that CDK4/6 inhibitors combined with ET markedly improve the survival rate of patients with advanced or metastatic HR^+^/HER2^–^ BC (3, 8–10). Consequently, 3 CDK4/6 inhibitors (palbociclib [Palbo], ribociclib, and abemaciclib [Abema]) have been approved by the US Food and Drug Administration (FDA) and European Medicines Agency (EMA) for treatment of patients with advanced or metastatic HR^+^/HER2^–^ BC (3, 10, 11). Despite the substantial therapeutic benefits of CDK4/6 inhibitors, development of intrinsic or acquired resistance poses a major clinical challenge (3).

Kinesin family members (KIFs) are microtubule-associated motor proteins, with 45 identified in humans and classified into 14 subfamilies based on motor domain homology (12, 13). Generally, the members of the kinesin 1 through 12, kinesin 13, and kinesin 14 subfamilies have a motor domain in their N-terminal, middle, and C-terminal region, respectively (13, 14). KIFs mainly rely on hydrolysis of adenosine triphosphate to transport various cargoes (such as vesicles, organelles, chromosomes, mRNAs, and proteins) along microtubules within cells (13, 15). Additionally, KIFs can regulate gene transcription (16), protein stability, and subcellular localization (17–19), thus regulating multiple intracellular signaling pathways. Consequently, dysregulation of KIFs can impair normal cellular functions and contribute to diseases, including cancer (12, 13, 15). Additionally, some KIFs, such as kinesin family member 10 (KIF10) and KIF11, have emerged as potential therapeutic targets for the development of anticancer drugs (12). Given the crucial role of KIFs in cells, clarifying their biological functions and related mechanisms in human cancer will facilitate identification of molecular markers and therapeutic targets.

Kinesin family member C2 (KIFC2) is a poorly characterized KIF protein that belongs to the kinesin 14 subfamily and is distinct from the kinesin 13 subfamily member KIF2C (kinesin family member 2C) (13). To date, there is relatively little information on the structure and function of the KIFC2 protein. Available evidence from mouse models shows that KIFC2 is mainly expressed in neural tissues and is involved in organelle transport in axons and dendrites (20, 21). Additionally, its expression levels are downregulated in learned helplessness mice (22) but are upregulated following chronic alcohol stimulation (23). Recently, 2 bioinformatic analyses indicate that KIFC2 is a potential prognostic biomarker for colon adenocarcinoma (24) and prostate cancer (25). Another study shows that KIFC2 mediates prostate cancer progression via regulation of transcription factor p65 (26). However, the biological functions and related mechanisms of KIFC2 in the progression and therapeutic resistance of BC have not yet been explored.

In this study, we found by integrative analyses of HR^+^/HER2^–^ BC data sets from FUSCC (Fudan University Shanghai Cancer Center) (27), TCGA (The Cancer Genome Atlas), and METABRIC (Molecular Taxonomy of Breast Cancer International Consortium) that *KIFC2* was amplified in approximately 50% HR^+^/HER2^–^ BCs and that its high expression was associated with poor prognosis, increased tumor protein p53 (*TP53*) somatic mutation, and active pyrimidine metabolism. Functional and mechanistic investigations further revealed that KIFC2 promoted growth and conferred resistance to Tam and CDK4/6 inhibitors in HR^+^/HER2^–^ BC by recruiting ubiquitin-specific peptidase 9 X-linked (USP9X) to stabilize CDK4. Overall, this study reveals that KIFC2 may serve as a promising therapeutic target and predictive biomarker for therapeutic responsiveness in HR^+^/HER2^–^ BC.

## Results

### KIFC2 is highly amplified in HR^+^/HER2^–^ BC, and its high expression is associated with poor patient prognosis.

**Figure 1:**
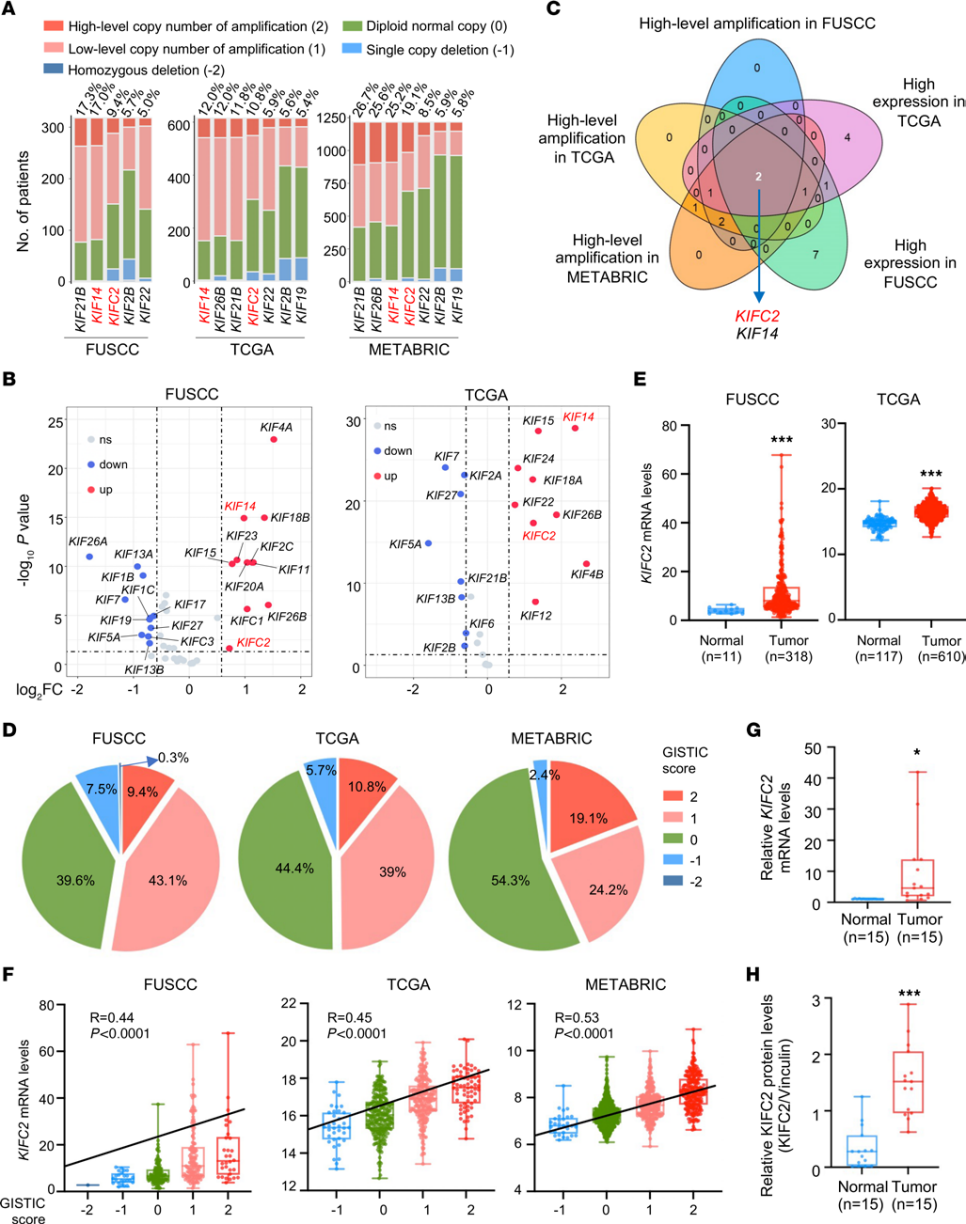
*KIFC2* is amplified and overexpressed in HR^+^/HER2^–^ BC. (**A**) High-level amplification of KIF members in over 5% of patients with HR^+^/HER2^–^ BC in the FUSCC (*n* = 318), TCGA (*n* = 610), and METABRIC (*n* = 1,217) data sets. (**B**) Differences in mRNA expression levels of the KIF family members between HR^+^/HER2^–^ BC tissues and normal samples in the FUSCC and TCGA data sets. (**C**) Venn diagram showing cross-analysis of the KIF family members, with both high-level amplification in over 5% patients in the FUSCC, TCGA, and METABRIC data sets and an upregulation in mRNA levels in the FUSCC and TCGA data sets. (**D**) CNA status of *KIFC2* in patients with HR^+^/HER2^–^ BC in the FUSCC, TCGA, and METABRIC data sets. (**E**) Analysis of mRNA levels of *KIFC2* in HR^+^/HER2^–^ BC cohorts from the FUSCC and TCGA data sets. Center lines represent the median. (**F**) Spearman’s correlation analysis of the relationship between the CNAs and mRNA expression levels of *KIFC2* in HR^+^/HER2^–^ BC from the FUSCC, TGCA, and METABRIC data sets. The black line indicates a correlation between CNA and mRNA; the center lines represent the median. (**G**) RT-qPCR analysis of *KIFC2* mRNA levels in 15 pairs of HR^+^/HER2^–^ BC tissues and matched noncancerous samples. The center lines represent the median. (**H**) Immunoblot analysis of KIFC2 protein levels in 15 pairs of HR^+^/HER2^–^ BC tissues and matched noncancerous samples. The center lines represent the median. **E**: Mann-Whitney *U* test; **G** and **H**: 2-tailed Student’s *t* test. *P < 0.05; ***P < 0.001.

Given the crucial roles of KIFs in cellular functions and human diseases (12, 13,15), we examined changes in expression of the KIF family members (Supplemental Table 1; supplemental material available online with this article; https://doi.org/10.1172/JCI183531DS1) in HR^+^/HER2^–^ BC by integrative analyses of HR^+^/HER2^–^ BC data sets from FUSCC (27), TCGA, and METABRIC. Of note, the FUSCC data set contains complete 4-dimensional data (*n* = 318), including copy number alternations (CNAs), RNA-Seq data, somatic mutations, and metabolomics (27). Interestingly, we found that 5 KIF genes with high-level copy number amplifications (Genomic Identification of Significant Targets in Cancer [GISTIC] score: 2) (28, 29) in over 5% of patients (Figure 1A and Supplemental Figure 1A). Similarly, 7 KIF genes displayed high-level amplifications in over 5% of patients in both the TCGA and METABRIC data sets (Figure 1A and Supplemental Figure 1, B and C). Moreover, analysis of transcriptional expression profiles indicated that 11 and 9 KIF genes were upregulated in HR^+^/HER2^–^ BC relative to normal control samples (log_2_ fold change [log_2_FC] > 0.58 corresponds to FC > 1.5-fold and *P* < 0.05) in the FUSCC and TCGA data sets, respectively (Figure 1B). Due to a lack of corresponding normal sample data, similar analysis could not be performed on the METABRIC data set. Cross-analysis of the above results identified only *KIFC2* and *KIF14* as concurrently amplified and upregulated in HR^+^/HER2^−^ BC (Figure 1C). As the functional roles of KIF14 in human cancer including BC have been documented previously (30, 31), we chose the poorly characterized KIFC2 as the focus of this study.

Next, we conducted an analysis of CNA events associated with *KIFC2*, and found that 52.5%, 49.8%, and 43.3% of patients with HR^+^/HER2^–^ BC had some form of *KIFC2* amplification (combined low-level and high-level) in the FUSCC, TCGA, and METABRIC data sets, respectively (Figure 1D). As expected, mRNA levels of *KIFC2* were markedly elevated in tumor compared with normal control tissues in both the FUSCC and TCGA data sets (Figure 1E). Consistently, a positive correlation between copy number amplification and elevated mRNA expression levels of *KIFC2* was observed in the these data sets (Figure 1F).

To validate these results, we obtained 15 pairs of HR^+^/HER2^−^ BC specimens and adjacent noncancerous tissues to examine mRNA levels of *KIFC2* by reverse transcription quantitative PCR (RT-qPCR) and protein levels by immunoblot assays. The results showed that expression levels were higher in tumor tissues than matched normal samples at both the mRNA (Figure 1G) and protein (Figure 1H and Supplemental Figure 1D) levels. Additionally, survival analyses in the FUSCC and METABRIC cohorts demonstrated that higher *KIFC2* mRNA levels were associated with poorer overall survival (OS) (Supplemental Figure 2, A and B). We did not observe a correlation of *KIFC2* mRNA levels with OS of patients with HR^+^/HER2^–^ BC in the TCGA data set, probably due to differences in sample characteristics (such as source and quantity) and in data processing methods (such as preprocessing steps and data standardization methods). Together, these findings suggest that *KIFC2* is highly amplified in HR^+^/HER2^–^ BC and its elevated expression levels are associated with poor clinical outcomes.

### KIFC2 promotes growth of HR^+^/HER2^–^ BC cells both in vitro and in vivo.

**Figure 2:**
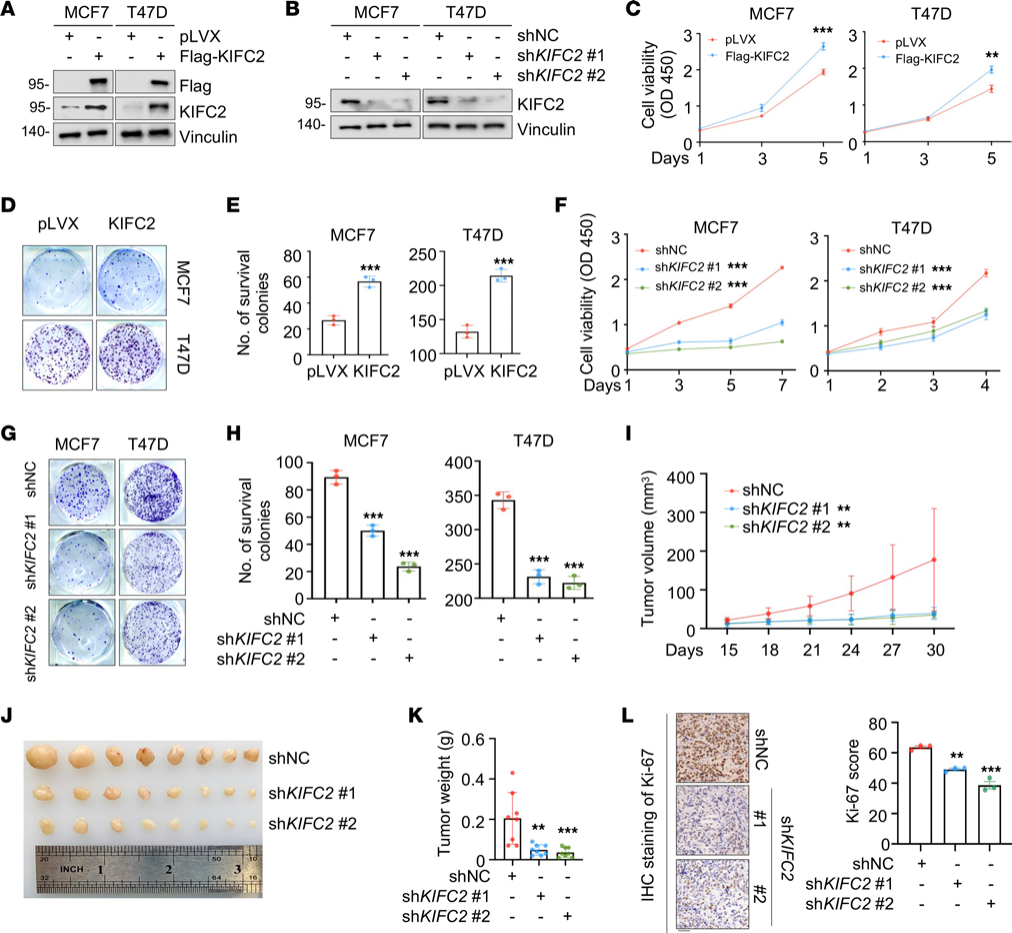
KIFC2 promotes the growth of HR^+^/HER2^–^ BC cells both in vitro and in mouse xenograft tumor models. (**A** and **B**) Immunoblot analysis of KIFC2 overexpression and knockdown efficiency in HR^+^/HER2^–^ BC cells stably expressing pLVX or Flag-KIFC2 (**A**) and shNC or sh*KIFC2* (1 and 2) (**B**). (**C**) Proliferation capacity of the cells shown in **A** was assessed using CCK-8 assays. (**D** and **E**) Colony formation assays of the cells shown in **A**. Images of surviving colonies (**D**) and results of quantitative analysis (**E**) are shown. (**F**) Proliferation capacity of the cells shown in **B** was assessed using CCK-8 assays. (**G** and **H**) Colony formation assays of the cells shown in **B**. Images of surviving colonies (**G**) and quantitative analysis (**H**) are shown. (**I**–**K**) MCF7 cells stably expressing shNC or sh*KIFC2* (1 and 2) were injected into the mammary fat pad of BALB/c female nude mice. Tumor growth rates (**I**), xenograft tumors (**J**), and tumor weight (**K**) are shown. (**L**) IHC detection of Ki-67 expression in mouse xenograft tumors. Representative images and quantitative analysis are shown. Scale bar: 50 μm. Data are mean ± SD; **C**, **E**, **F**, **H**, and **L**, *n* = 3 per group; **I** and **K**, *n* = 8 per group. **C** and **E**: 2-tailed Student’s *t* test; **F**, **H**, **I**, **K**, and **L**: 1-way ANOVA. **P < 0.01; ***P < 0.001.

To determine the biological functions of KIFC2 in HR^+^/HER2^–^ BC, we stably overexpressed Flag-KIFC2 or depleted endogenous KIFC2 in HR^+^/HER2^–^ MCF7 and T47D cells (32) by lentiviral infection. Of note, few HR^+^/HER2^–^ BC cell lines are available to date (32). Expression of KIFC2 in these established stable cell lines was verified by immunoblot assays (Figure 2, A and B). Cell Counting Kit-8 (CCK-8) and colony formation assays showed that ectopic expression of KIFC2 promoted proliferation (Figure 2C) and colony formation capacity (Figure 2, D and E) of MCF7 and T47D cells. Conversely, knockdown of endogenous KIFC2 in MCF7 and T47D cells reduced cell viability (Figure 2F) and colony formation ability in these cells (Figure 2, G and H).

Next, we assessed the effects of KIFC2 on the tumorigenic ability of HR^+^/HER2^–^ BC cells in xenograft tumor models in mice. The depletion of KIFC2 delayed tumor growth (Figure 2, I–K). Immunohistochemical (IHC) staining further demonstrated that the proportion of proliferation marker Ki-67–positive cells was lower in KIFC2-depleted tumors than in control samples (Figure 2L). Collectively, those results demonstrated that KIFC2 promoted the growth of HR^+^/HER2^–^ BC cells.

### Depletion of KIFC2 enhances the sensitivity of HR^+^/HER2^–^ BC cells to Tam and CDK4/6 inhibitors.

**Figure 3:**
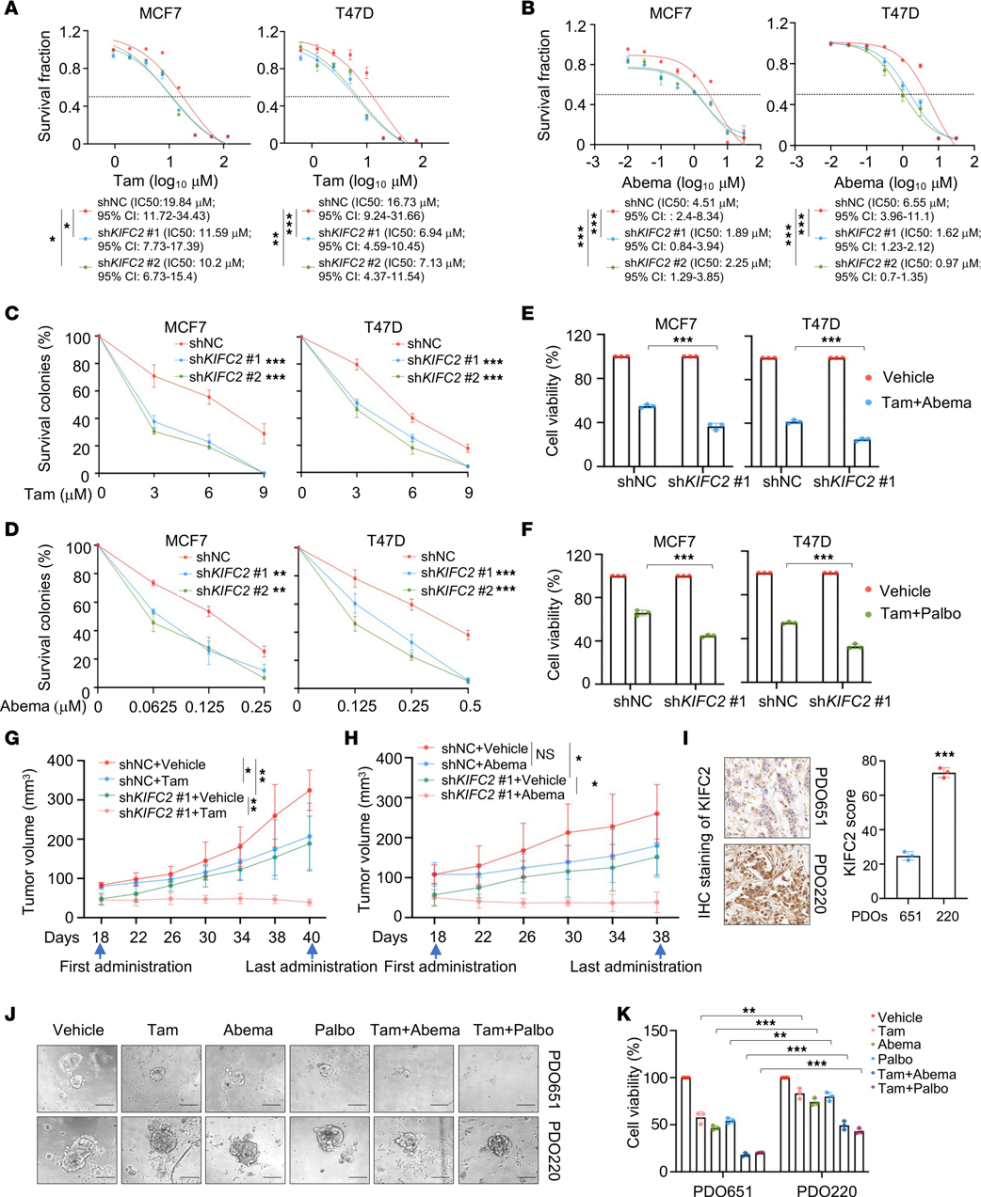
Knockdown of KIFC2 enhances the sensitivity of HR^+^/HER2^–^ BC cells to the ET drug Tam and the CDK4/6 inhibitor Abema. (**A** and **B**) Cells stably expressing shNC or sh*KIFC2* (1 and 2) were treated with increasing concentrations of Tam (**A**) and Abema (**B**) for 72 hours. IC_50_ values were determined using CCK-8 assays. (**C** and **D**) Cells stably expressing shNC or sh*KIFC2* (1 and 2) were subjected to clonogenic survival assays with increasing concentrations of Tam (**C**) and Abema (**D**) for 7–9 days. Quantitative analyses are shown. (**E** and **F**) Cells stably expressing shNC or sh*KIFC2* 1 were treated with vehicle, Tam combined with Abema (**E**), or Tam combined with Palbo (**F**) for 72 hours, then subjected to CCK-8 assays. (**G** and **H**) MCF7 cells with stable shNC or sh*KIFC2* 1 expression were injected into BALB/c nude mice. After 18 days, mice were treated with vehicle, Tam (**G**), or Abema (**H**). Tumor growth rates are shown. (**I**) Expression status of KIFC2 in HR^+^/HER2^–^ BC PDOs was assessed by IHC staining of postoperative pathological tissue slices from the same patients. Scale bars: 50 μm. (**J** and **K**) CellTiter-Glo 3D assays were conducted in HR^+^/HER2^–^ BC PDOs treated with Tam, Abema, Palbo, or their combinations. Representative images (**J**) and quantitative data (**K**) are shown. Scale bars: 100 μm. Data are mean ± SD; **A**–**F**, **I**, and **K**, *n* = 3 per group; **G** and **H**, *n* = 6 per group. **A** and **B**: extra-sum-of-squares *F* test; **C**, **D**, **G**, and **H**: 1-way ANOVA; **E**, **F**, **I**, and **K**: 2-tailed Student’s *t* test. *P < 0.05; **P < 0.01; ***P < 0.001.

ET combined with CDK4/6 inhibitors has emerged as a standard-of-care treatment for patients with advanced or metastatic HR^+^/HER2^–^ BC (1, 8, 10). Thus, we next evaluated the effects of KIFC2 on cellular sensitivity of HR^+^/HER2^–^ BC to the most-used ET drug, Tam, and the FDA/EMA-approved CDK4/6 inhibitors Abema and Palbo (10). The results showed that knockdown of KIFC2 resulted in a reduction in IC_50_ of Tam (Figure 3A), Abema (Figure 3B), and Palbo (Supplemental Figure 3A) in MCF7 and T47D cells. Consistent with this finding, colony formation assays demonstrated that KIFC2-depleted MCF7 and T47D cells were more sensitive to Tam (Figure 3C and Supplemental Figure 3B), Abema (Figure 3D and Supplemental Figure 3C), and Palbo (Supplemental Figure 3, D and E) than shNC-expressing control cells. Moreover, knockdown of KIFC2 also enhanced cellular sensitivity to Tam combined with Abema (Figure 3E) or with Palbo (Figure 3F).

To validate these findings in vivo, we generated mouse xenograft tumor models derived from MCF7 cells. As expected, tumors with KIFC2 knockdown showed slower growth and greater responsiveness to Tam (Figure 3G and Supplemental Figure 4, A–D) or Abema (Figure 3H and Supplemental Figure 4, E–H). Next, we evaluated the effects of KIFC2 on drug sensitivity in HR^+^/HER2^–^ BC patient-derived organoids (PDOs). The expression status of KIFC2 in PDOs was assessed by IHC staining of postoperative pathological tissue slices from the same patients (Figure 3I). The results showed that PDO651, with low expression of KIFC2, was more sensitive to Tam, Abema, and Palbo alone or to Tam combined with Abema or with Palbo than PDO220, with high KIFC2 expression (Figure 3, J and K). We must acknowledge that other factors besides KIFC2 may have influenced the sensitivity of PDOs to these tested drugs given the heterogeneity of their genetic backgrounds. Taken together, these results suggest that depletion of KIFC2 enhances the sensitivity of HR^+^/HER2^–^ BC cells to Tam and CDK4/6 inhibitors.

### Amplification of KIFC2 is associated with increased TP53 somatic mutation and active pyrimidine metabolism.

As somatic mutations in cancer driver genes and metabolic dysregulation are intimately linked to the progression and therapeutic responsiveness of HR^+^/HER2^–^ BC (27), we next delineated the discrepancies in somatic mutational profiles between the tumors with and without *KIFC2* amplification in the FUSCC (27), TCGA, and METABRIC data sets. The results showed an increased frequency of *TP53* mutations in the samples with *KIFC2* amplification (Supplemental Figure 5, A and B). Conversely, the tumors with *TP53* mutations displayed an increased frequency of *KIFC2* gene amplification (Supplemental Figure 5C).

To further examine the effects of *TP53* mutation on KIFC2 expression levels, we knocked down endogenous p53 in WT p53–expressing MCF7 cells and then reexpressed WT p53 or several of the most-reported mutant p53 variants (R175H, Y220C, R248W, and R273H). Immunoblot analysis showed that knockdown of p53 resulted in a marked upregulation of KIFC2 protein levels, and this effect was partially reversed by reexpression of WT p53 but not mutant p53 variants (Supplemental Figure 6A). These results suggest that WT p53 negatively regulated KIFC2 expression, whereas mutant p53 lacked the noted inhibitory effects on KIFC2 expression.

To examine the functional roles of KIFC2 in cells expressing WT or mutant p53, we knocked down endogenous p53 in WT p53–expressing MCF7 cells and then reexpressed Flag-KIFC2 alone or in combination with WT p53 or p53-mutant R175H (the mutation with the highest frequency in patients with HR^+^/HER2^–^ BC in the TCGA and METABRIC data sets) (Supplemental Figure 6, B and C). Functional assays demonstrated that expression of WT p53, but not p53-mutant R175H, attenuated KIFC2-mediated growth-promoting and drug-resistant phenotypes (Supplemental Figure 6, D–G). These findings suggest that KIFC2 may play different roles in promoting HR^+^/HER2^–^ BC progression and therapeutic resistance depending on the genetic background of p53.

Next, we analyzed the impact of *KIFC2* amplification on metabolic pathway alterations using metabolomic (Supplemental Figure 7A) and transcriptomic (Supplemental Figure 7B) data from the FUSCC cohort (27). Further cross-analysis revealed consistent upregulation of pyrimidine metabolism and downregulation of propionate metabolism in *KIFC2*-amplified tumors (Supplemental Figure 7C). Accumulating evidence has shown that pyrimidine metabolism is pivotal in cancer progression and therapy resistance in human cancer (33, 34). In contrast, the role of the propionate metabolism pathway in human cancer is less well understood. Thus, we then focused on investigating the correlation between pyrimidine metabolism and *KIFC2* amplification in this study. Spearman’s correlation analysis showed a positive relationship between pyrimidine metabolism and *KIFC2* amplification (Supplemental Figure 7D).

To further validate these results, we conducted liquid chromatography–tandem mass spectrometry–based (LC-MS/MS–based) metabolomics to evaluate the impact of KIFC2 knockdown on pyrimidine metabolism. Kyoto Encyclopedia of Genes and Genomes (KEGG) pathway enrichment analysis revealed that the metabolites that were downregulated upon KIFC2 knockdown were enriched in the pyrimidine metabolism pathway (Supplemental Figure 7E). And 4 pyrimidine metabolites were consistently downregulated in KIFC2-knocked down cells (Supplemental Figure 7F). These results further support the notion that *KIFC2* amplification may be involved in pyrimidine metabolism.

We next evaluated whether KIFC2 affects cellular sensitivity to chemotherapy agents targeting pyrimidine metabolism. As shown in Supplemental Figure 8, A–C, KIFC2-overexpressing cells exhibited increased sensitivity to the antimetabolite chemotherapy agent capecitabine, which is effective for the clinical treatment of breast cancer (35). Consistently, PDO518, with high KIFC2 expression, showed increased sensitivity to capecitabine compared with PDO474, with low expression of KIFC2 (Supplemental Figure 8, D–G). Together, these results indicate that antimetabolite chemotherapy agents such as capecitabine might be effective for the treatment of patients with HR^+^/HER2^–^ BC with high KIFC2 expression.

### KIFC2 interacts with CDK4 and enhances its protein stability.

**Figure 4:**
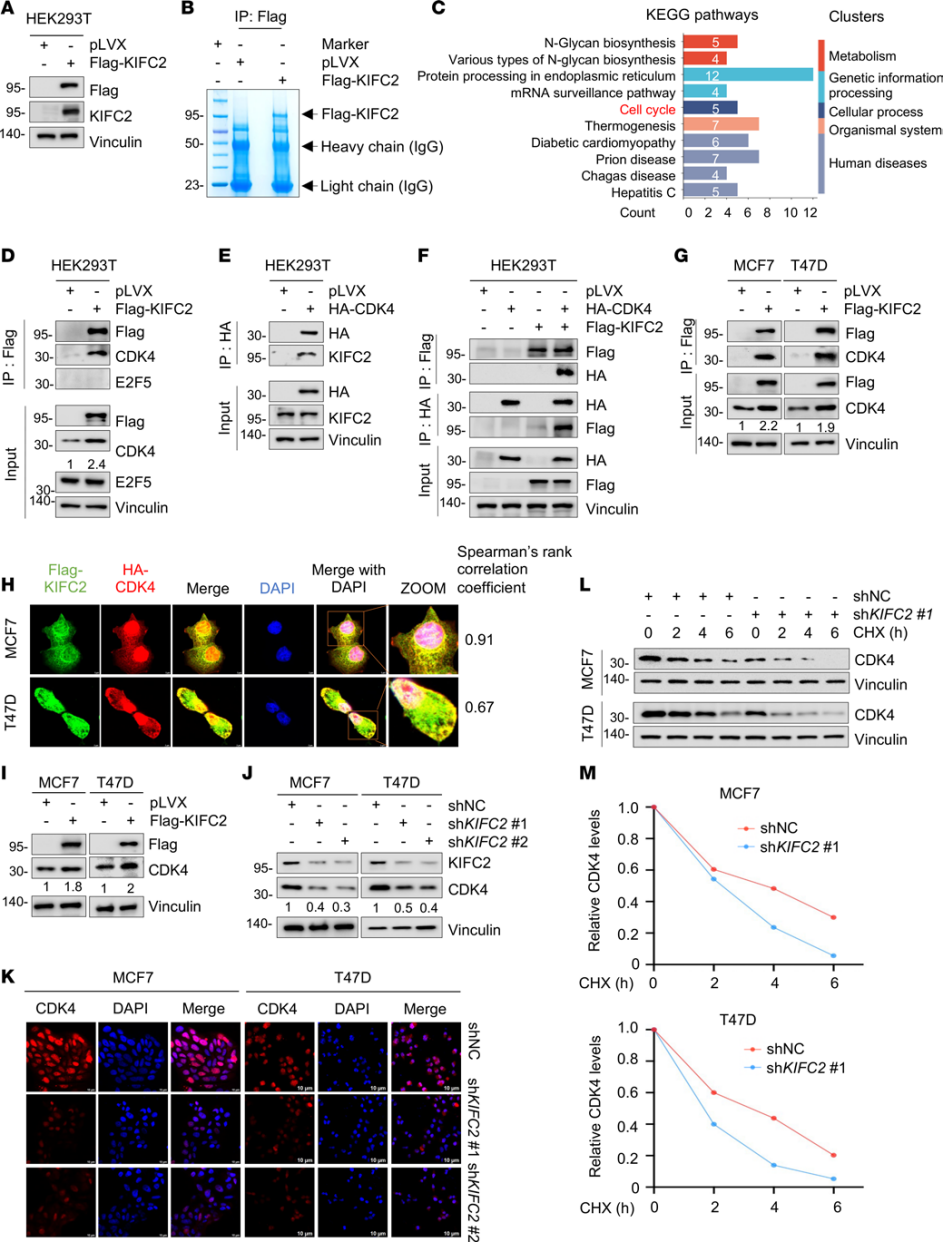
KIFC2 interacts with CDK4 and enhances its protein stability. (**A** and **B**) IP assays were performed on HEK293T cells expressing pLVX and Flag-KIFC2 with anti-Flag magnetic beads (**A**), followed by LC-MS/MS analysis of the gels (**B**). (**C**) KEGG pathway enrichment analysis of the 165 identified KIFC2-interacting proteins. (**D**–**F**) HEK293T cells were transfected with pLVX, Flag-KIFC2, or HA-CDK4 plasmids alone or in combination, then subjected to sequential IP and immunoblot assays with the indicated antibodies. (**G**) MCF7 and T47D cells stably expressing pLVX or Flag-KIFC2 were subjected to IP and immunoblot assays with the indicated antibodies. (**H**) IF staining was performed to examine the colocalization of Flag-KIFC2 (green) and HA-CDK4 (red) in MCF7 and T47D cells. Nuclei were visualized by counterstaining with DAPI. Spearman’s rank correlation coefficient for both proteins was calculated with ImageJ software. Scale bars: 5 μm. (**I** and **J**) Immunoblot analysis of CDK4 protein levels in MCF7 and T47D cells with ectopic expression (**I**) or knockdown (**J**) of KIFC2. (**K**) IF staining was performed to analyze CDK4 protein levels in HR^+^/HER2^–^ BC cells with stable shNC or sh*KIFC2* (1 and 2) expression. Scale bar: 10 μm. (**L** and **M**) MCF7 and T47D cells stably expressing shNC or sh*KIFC2* 1 were treated with or without 100 μg/mL CHX for the indicated times, then subjected to immunoblot assays (**L**). (**M**) Relative protein levels of CDK4 (CDK4/vinculin).

To elucidate the mechanisms underlying KIFC2 function in HR^+^/HER2^−^ BC, we performed immunoprecipitation coupled with LC-MS/MS in human embryonic kidney 293T (HEK293T) cells expressing pLVX or Flag-KIFC2 to examine the binding partners of KIFC2 (Figure 4, A and B). This approach identified 165 proteins as potential KIFC2 interactors, each with at least 3 unique peptide matches with confidence levels exceeding 95%. KEGG enrichment analysis found that these identified proteins were mainly involved in metabolism (N-glycan biosynthesis), genetic information processing (protein processing in endoplasmic reticulum and mRNA surveillance), cellular process (cell cycle), organismal system (thermogenesis), and human diseases (diabetic cardiomyopathy, prion disease, Chagas disease, and hepatitis C) (Figure 4C). Given the vital roles of cell-cycle pathway in the progression and therapeutic responsiveness of HR^+^/HER2^–^ BC (3), we focused on the identified cell cycle–related proteins CDK4, protein phosphatase 2 catalytic subunit beta (PPP2CB), E2 transcription factor 5 (E2F5), tyrosine 3-monooxygenase/tryptophan 5-monooxygenase activation protein gamma (YWHAG), and transcription factor Dp-1 (TFDP1) for further investigation (Supplemental Figure 9A). Previous studies have well documented that CDK4 serves as a critical regulator of the G_1_- to S-phase transition in the cell cycle and that its overexpression contributes to resistance to Tam and CDK4/6 inhibitors (3, 6, 36). In contrast, there are relatively few reports concerning the functional roles of PPP2CB, E2F5, YWHAG, and TFDP1 in cell-cycle progression of BC.

Next, we carried out IP assays to determine whether KIFC2 interacts with CDK4. Reciprocal IP assays revealed an interaction between KIFC2 and CDK4 in HEK293T cells (Figure 4, D–F). Additionally, we noticed that KIFC2 did not bind to transcription factor E2F5 (Figure 4D). The interaction between KIFC2 and CDK4 was further confirmed by IP assays in MCF7 and T47D cells (Figure 4G). Moreover, immunofluorescence staining (IF) demonstrated that Flag-KIFC2 partially colocalized with HA-CDK4 in MCF7 and T47D cells (Figure 4H, yellow). Together, these results suggest that KIFC2 is a binding partner of CDK4.

We next investigated the mutual regulatory relationship between KIFC2 and CDK4. The results showed that overexpression of KIFC2 led to upregulation (Figure 4I), whereas knockdown of KIFC2 resulted in downregulation (Figure 4, J and K), of protein levels of CDK4. However, neither ectopic expression nor depletion of KIFC2 affected *CDK4* mRNA levels (Supplemental Figure 9, B and C), indicating that regulation of CDK4 by KIFC2 occurs at the posttranscriptional level. Conversely, neither overexpression nor knockdown of CDK4 influenced protein levels of KIFC2 (Supplemental Figure 9, D and E). These findings collectively indicate that KIFC2 positively regulated the protein levels of CDK4. Chase assays using the protein synthesis inhibitor cycloheximide (CHX) further demonstrated that silencing of endogenous KIFC2 shortened the half-life of CDK4 protein in MCF7 and T47D cells (Figure 4, L and M), indicating that KIFC2 enhanced the stability of CDK4 protein.

Given the pivotal role of CDK4 in phosphorylating RB and G_1_- to S-phase transition of the cell cycle (3), we next assessed expression levels of RB phosphorylation and its downstream effector cyclin A2 by immunoblotting. The results showed that expression levels of RB phosphorylation and cyclin A2 were reduced upon KIFC2 knockdown (Supplemental Figure 10A). Consistently, cell-cycle analysis by FACS showed an increase in the number of cells in G_1_ phase, along with a reduction in the proportion of cells in the S phase following KIFC2 depletion (Supplemental Figure 10, B–D).

### KIFC2 suppresses ubiquitin-dependent proteasomal degradation of CDK4.

**Figure 5:**
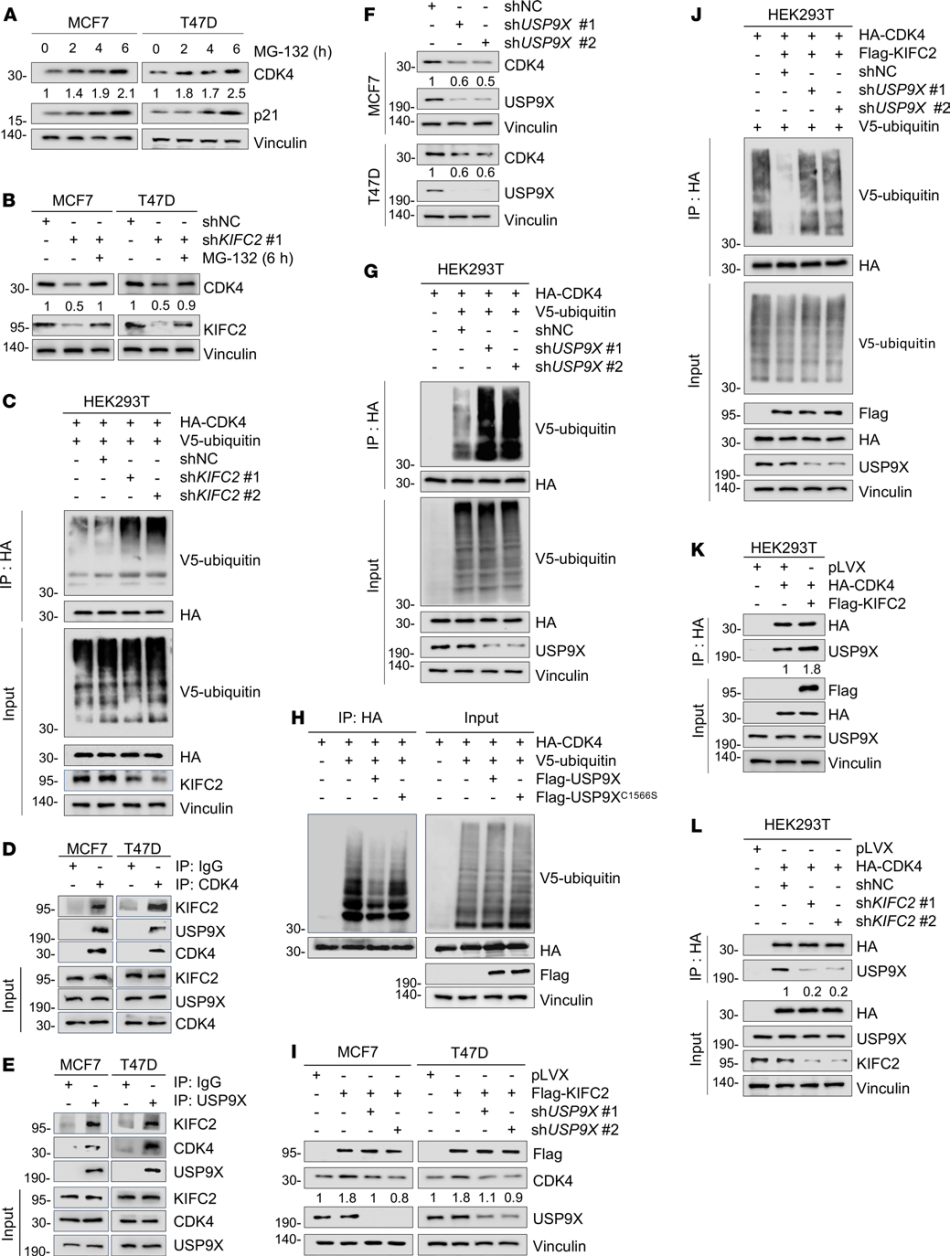
KIFC2 recruits USP9X to stabilize CDK4. (**A**) MCF7 and T47D cells were treated with or without 10 μM MG-132, then subjected to immunoblot assays. (**B**) MCF7 and T47D cells stably expressing shNC or sh*KIFC2* 1 were treated with or without MG-132, then subjected to immunoblot assays. (**C**) Detection of CDK4 ubiquitination levels in KIFC2-knockdown cells. (**D** and **E**) Endogenous interaction of KIFC2, CDK4, and USP9X in MCF7 and T47D cells. (**F**) MCF7 and T47D cells expressing shNC or sh*USP9X* (1 and 2) were subjected to immunoblot assays. (**G**) Detection of CDK4 ubiquitination levels in USP9X-knockdown cells. (**H**) HEK293T cells were transfected with the indicated plasmids for 48 hours, then incubated with MG-132 for another 6 hours. Cellular lysates were collected for IP and immunoblot assays. (**I**) Immunoblot analysis of CDK4 protein levels in KIFC2-overexpressing cells infected with shNC or sh*USP9X* (1 and 2) lentiviruses. (**J**) HEK293T cells were transfected with plasmids for 24 hours, infected with shNC or sh*USP9X* (1 and 2) viruses for 48 hours, and incubated for 6 hours with MG-132. Cells were collected for IP and immunoblotting. (**K** and **L**) Detection of the interaction between USP9X and CDK4 in KIFC2-overexpressing (**K**) or -knockdown (**L**) cells.

In eukaryotic cells, protein degradation primarily occurs through 2 major routes, including the ubiquitin-proteasome pathway and the autophagy-lysosome system (37). To address the molecular mechanisms of KIFC2-mediated CDK4 stabilization, we treated MCF7 and T47D cells with the proteasome inhibitor MG-132 or autophagy inhibitor bafilomycin-A1 (Baf-A1). Notably, treatment with MG-132 resulted in a time-dependent increase in levels of CDK4 and p21 (positive control) (Figure 5A) and partially restored the downregulated CDK4 protein levels caused by KIFC2 depletion (Figure 5B). In contrast, administration of Baf-A1 did not markedly affect CDK4 expression levels (Supplemental Figure 11A) or rescue the decreased CDK4 levels following KIFC2 knockdown (Supplemental Figure 11B). Additionally, ubiquitination assays demonstrated that ubiquitination levels of CDK4 were decreased by KIFC2 overexpression (Supplemental Figure 11C; compare lane 4 with lane 3), while they were increased by KIFC2 depletion (Figure 5C, compare lanes 3 and 4 with lane 2). These results indicate that KIFC2 suppressed ubiquitination-dependent proteasomal degradation of CDK4.

### KIFC2 recruits deubiquitinase USP9X to stabilize CDK4.

As KIFC2 is not a putative deubiquitinase (DUB), we speculated that KIFC2 might recruit certain DUBs to mediate CDK4 deubiquitination. By analyzing the potential KIFC2-interacting proteins identified above by LC-MS/MS assays (Figure 4, A and B), we noticed that the DUB USP9X was a potential partner of KIFC2 (Supplemental Figure 11D). Moreover, we found that mRNA levels of *USP9X* were upregulated in HR^+^/HER2^–^ BC specimens relative to normal tissues (Supplemental Figure 11E) and that its high expression was associated with poor OS of patients with HR^+^/HER2^–^ BC (Supplemental Figure 11F) in the FUSCC data set (27).

We next examine whether KIFC2 interacts with USP9X and CDK4. Reciprocal IP assays demonstrated that Flag-KIFC2 interacted with endogenous CDK4 and USP9X in HEK293T, MCF7, and T47D cells (Supplemental Figure 11, G and H). And Flag-CDK4 also interacted with endogenous KIFC2 and USP9X (Supplemental Figure 11, I and J). IF further revealed partial colocalization between Flag-KIFC2 and USP9X, as well as between Flag-CDK4 and USP9X in MCF7 and T47D cells (Supplemental Figure 11, K and L). At the endogenous level, IP using CDK4 and USP9X antibodies also detected interactions with KIFC2 in MCF7 and T47D cells (Figure 5, D and E), despite the unavailability of a suitable KIFC2 antibody for IP. These findings indicate that KIFC2, CDK4, and USP9X may form a ternary complex in HR^+^/HER2^–^ BC cells.

Next, we assessed the impact of USP9X on expression and ubiquitination levels of CDK4. The results indicated that USP9X knockdown decreased CDK4 protein levels without affecting mRNA (Figure 5F and Supplemental Figure 12A), increased CDK4 ubiquitination (Figure 5G, compare lanes 3 and 4 with lane 2), and shortened CDK4 protein half-life (Supplemental Figure 12, B and C). Notably, ectopic expression of WT USP9X, but not its catalytically inactive mutant C1566S (38), resulted in a reduction in CDK4 ubiquitination (Figure 5H), suggesting that USP9X promotes CDK4 deubiquitination in a manner that depends on its DUB activity. In support of this notion, treatment with the USP9X inhibitor WP1130 (39) attenuated USP9X-mediated deubiquitination of CDK4 (Supplemental Figure 12, D and E) and reduced CDK4 protein levels (Supplemental Figure 12F). Together, these results suggest that USP9X acts as a DUB for CDK4 protein stability.

Next, we sought to explore whether KIFC2-induced CDK4 stability depends on USP9X. Immunoblot assays showed that overexpression of KIFC2 induced an increase in CDK4 protein levels, but this effect was impaired upon USP9X knockdown (Figure 5I). Furthermore, silencing of USP9X could reverse the inhibitory effects of KIFC2 on CDK4 ubiquitination levels (Figure 5J, compare lanes 3 and 4 with lane 2). Consistently, administration of WP1130 (39) attenuated KIFC2-mediated deubiquitination of CDK4 (Supplemental Figure 12G) and compromised KIFC2-induced upregulation of CDK4 protein levels (Supplemental Figure 12H). Conversely, knockdown of KIFC2 impaired USP9X-mediated deubiquitination of CDK4 (Supplemental Figure 12I). These results collectively suggest that KIFC2 cooperates with USP9X to mediate CDK4 deubiquitination.

Additionally, we noticed that overexpression or knockdown of KIFC2 did not affect expression of USP9X (Supplemental Figure 12, J and K). Thus, we proposed that KIFC2 regulates CDK4 stability, possibly by influencing the interaction between USP9X and CDK4. Indeed, IP assays demonstrated that the interaction between USP9X and CDK4 was enhanced following KIFC2 overexpression (Figure 5K, compare lane 3 with lane 2), whereas it was decreased by KIFC2 depletion (Figure 5L, compare lanes 3 and 4 with lane 2). Collectively, these results suggest that KIFC2 recruits USP9X to stabilize CDK4.

### KIFC2 boosts the growth-promoting and drug-resistant phenotypes of HR^+^/HER2^–^ BC cells partially though regulation of CDK4.

**Figure 6:**
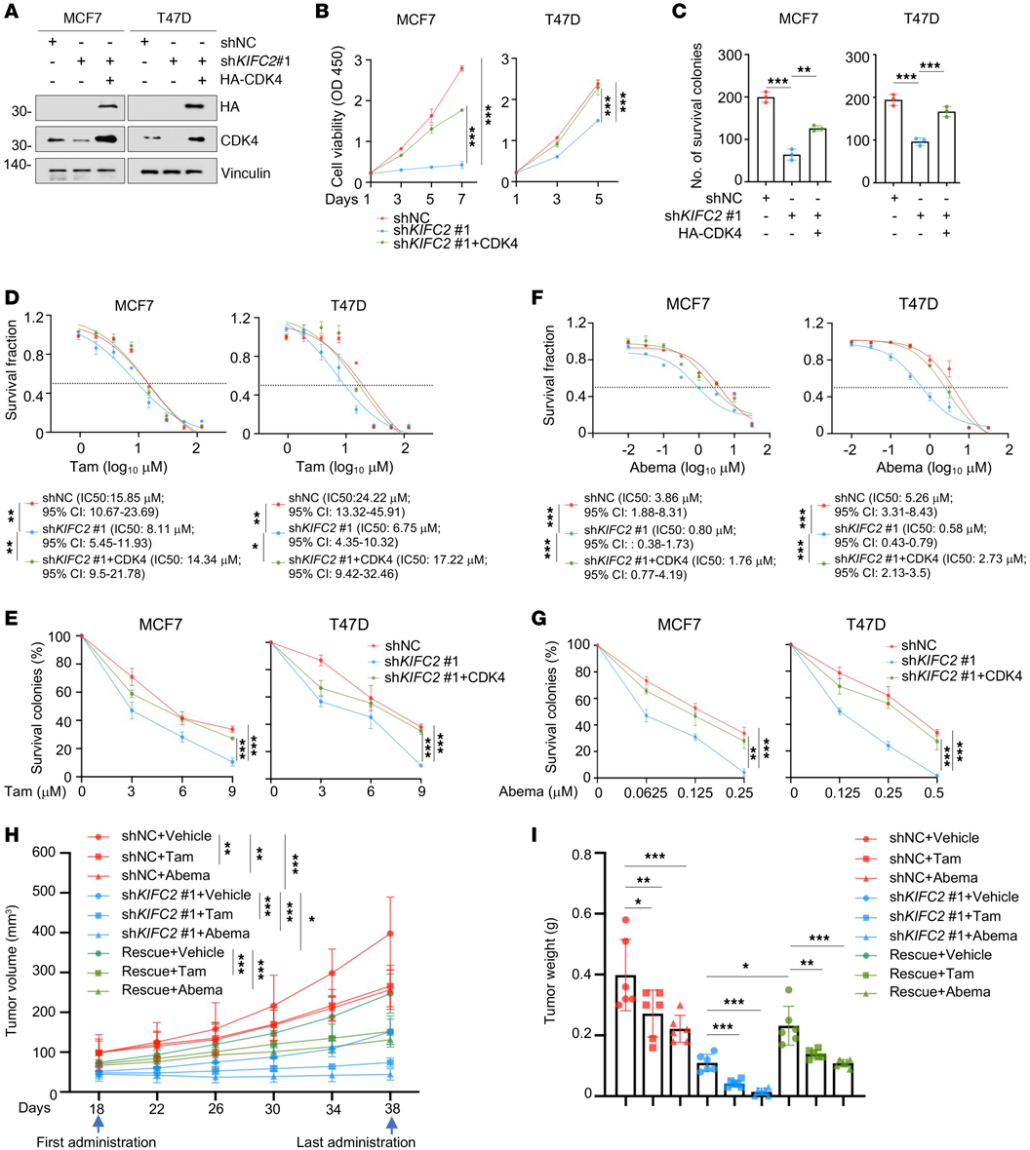
KIFC2 boosts the growth-promoting and drug-resistant phenotypes of HR^+^/HER2^–^ BC cells partially though regulation of CDK4. (**A**) MCF7 and T47D cells stably expressing shNC or sh*KIFC2* 1 alone or in combination with HA-CDK4 were subjected to immunoblot assays. (**B** and **C**) CCK-8 (**B**) and colony formation (**C**) assays were performed using the cells shown in **A**. (**D**) The cells shown in **A** were treated with increasing concentrations of Tam for 72 hours and underwent CCK-8 assays for determination of IC_50_ values. (**E**) Clonogenic survival assays were performed using the cells shown in **A** in the presence or absence of Tam for 7–9 days. (**F**) The cells shown in **A** were treated with increasing concentrations of Abema for 72 hours and underwent CCK-8 assays for determination of IC_50_ values. (**G**) Clonogenic survival assays were performed using the cells shown in **A** in the presence or absence of Abema for 7–9 days. (**H** and **I**) MCF7 cells stably expressing shNC, sh*KIFC2* 1, or sh*KIFC2* 1 + HA-CDK4 were injected into the mammary fat pad of BALB/c female nude mice. After 18 days of injection, mice in each group were administered vehicle, Tam, or Abema. (**H**) Tumor volume. (**I**) Tumor weight. Rescue refers to KIFC2-knockdown cells with CDK4 reexpression. Data are mean ± SD; **B**–**G**, *n* = 3 per group; **H** and **I**, *n* = 6 per group). **B**, **C**, **E**, **G**, **H**, and **I**: 1-way ANOVA; **D** and **F**: extra-sum-of-squares *F* test. *P < 0.05; **P < 0.01; ***P < 0.001.

To explore whether KIFC2 exerts its oncogenic functions via CDK4, we reintroduced CDK4 into KIFC2-depleted cells for rescue experiments in vitro and in vivo (Figure 6A). The results showed that CDK4 reexpression restored proliferation and colony formation impaired by KIFC2 knockdown in MCF7 and T47D cells (Figure 6, B and C, and Supplemental Figure 13A) and partially reversed their increased sensitivity to Tam, Abema, and Palbo (Figure 6, D–G, and Supplemental Figure 13, B–F). In vivo assays using xenograft tumor models in mice further demonstrated that reduced tumor growth and enhanced sensitivity to Tam and Abema caused by silencing of KIFC2 were partially reversed by CDK4 overexpression (Figure 6, H and I, and Supplemental Figure 13, G–I). Together, these results suggest that KIFC2 promotes growth and confers resistance to Tam and CDK4/6 inhibitors partially by regulating CDK4 in HR^+^/HER2^–^ BC cells.

To further validate these results, we next investigated the effects of USP9X knockdown on cellular sensitivity to Tam and CDK4/6 inhibitors alone or in combination. The results showed that knockdown of USP9X enhanced cellular sensitivity to Tam, Abema, and Palbo (Supplemental Figure 14, A–J) or to Tam combined with Abema or with Palbo (Supplemental Figure 14, K and L). Moreover, we found that the USP9X inhibitor WP1130 enhanced the sensitivity of resistant PDO220 to Tam, Abema, and Palbo (Supplemental Figure 15, A and B). These results suggest that targeting USP9X could be an effective strategy to overcome therapeutic resistance in these models.

To investigate whether the KIFC2-USP9X/CDK4 axis is involved in the acquired resistance to ET and CDK4/6 inhibitors, we determined expression levels of KIFC2 and USP9X in parental, Tam-resistant (TamR), and Palbo-resistant (PalboR) MCF7 cell lines. The results showed that expression levels of KIFC2 and USP9X were upregulated in TamR and PalboR cell lines compared with their parental counterparts (Supplemental Figure 16A). Moreover, treatment with WP1130 (39) partially restored sensitivity of the MCF7-TamR and MCF7-PalboR cells to Tam (Supplemental Figure 16, B–D) and Palbo (Supplemental Figure 16, E–G), respectively. Collectively, these findings highlight the potential contributions of KIFC2-USP9X/CDK4 axis to therapeutic resistance to ET and CDK4/6 inhibitors alone or in combination.

### High KIFC2 mRNA expression is associated with poor survival of patients with HR^+^/HER2^–^ BC receiving adjuvant ET alone or in combination with CDK4/6 inhibitors.

**Figure 7:**
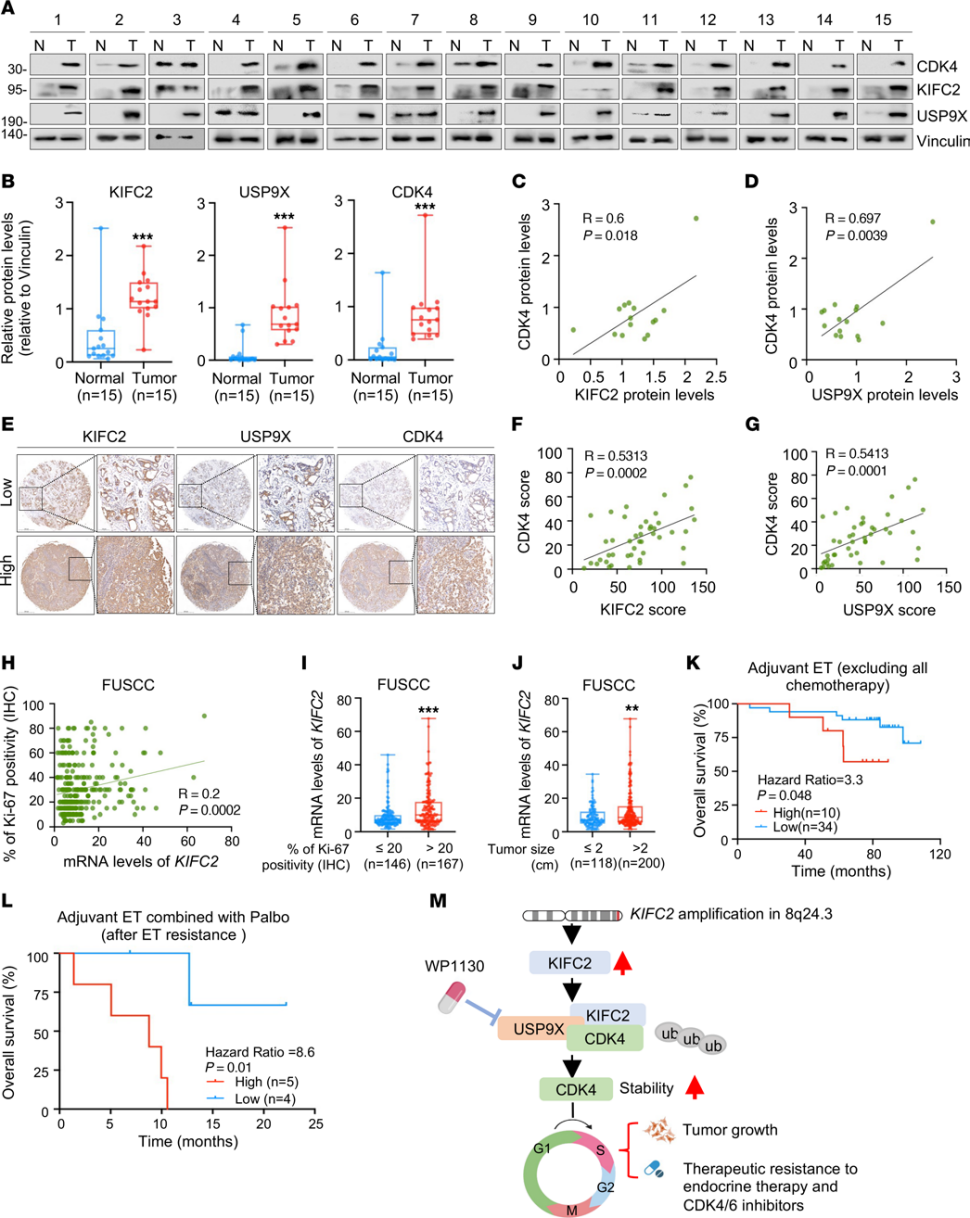
Clinical relevance of the KIFC2-USP9X/CDK4 axis in HR^+^/HER2^–^ BC. (**A** and **B**) Immunoblotting of CDK4, KIFC2, and USP9X in 15 pairs of HR^+^/HER2^–^ BC (T) and matched noncancerous (N) tissues (**A**), with quantification shown in **B**. Center lines indicate the median. (**C** and **D**) Pearson’s analysis of CDK4-KIFC2 (**C**) and CDK4-USP9X (**D**) correlations in the samples represented in **A**. (**E**) IHC analysis of KIFC2, USP9X, and CDK4 in 45 HR^+^/HER2^–^ BC samples. Top row, low protein expression; bottom row, high protein expression. Scale bars: 500 μm (left image of each pair [low-magnification view] and 100 μm (right image [high-magnification view]). (**F** and **G**) Pearson’s analysis of CDK4-KIFC2 (**F**) and CDK4-USP9X (**G**) correlations in the samples represented in **E**. (**H**) Pearson’s correlation analysis of *KIFC2* mRNA expression and Ki-67 protein levels (IHC) in HR^+^/HER2^–^ BC in the FUSCC cohort. (**I** and **J**) The relationship between *KIFC2* mRNA expression levels and the percentage of Ki-67 positivity (**I**) and tumor size (**J**) in HR^+^/HER2^–^ BC in the FUSCC cohort. Center lines indicate the median. (**K** and **L**) Kaplan-Meier analysis of overall survival of patients with HR^+^/HER2^–^ BC (FUSCC cohort) with adjuvant ET (excluding all chemotherapy) alone (**K**) or in combination with CDK4/6 inhibitors (led by Palbo) after ET resistance (**L**). Patients were stratified according to high and low expression levels of KIFC2. (**M**) Proposed working model. *KIFC2* amplification at chromosome 8q24.3 leads to increased expression in HR^+^/HER2^–^ BC. KIFC2 enhances CDK4 stability by recruiting USP9X, resulting in acceleration of the G_1_- to S-phase transition and promoting tumor growth and resistance to ET and CDK4/6 inhibitors. The USP9X inhibitor WP1130 could reverse KIFC2-driven growth-promoting and drug-resistant phenotypes in HR^+^/HER2^–^ BC. **B**: 2-tailed Student’s *t* test; **I** and **J**: Mann-Whitney *U* test; **K** and **L**: log-rank test. ub, ubiquitin. **P < 0.01; ***P < 0.001.

To validate the clinical relevance of the KIFC2-USP9X/CDK4 axis in HR^+^/HER2^–^ BC, we collected another (in addition to those shown in Supplemental Figure 1D) 15 pairs of HR^+^/HER2^–^ BC specimens and matched adjacent normal samples to determine expression levels of KIFC2, USP9X, and CDK4 by immunoblotting. The results revealed that protein expression levels of KIFC2, USP9X, and CDK4 were elevated in HR^+^/HER2^–^ BC tissues relative to matched normal controls (Figure 7, A and B) and that there was a positive correlation in protein levels between KIFC2 and CDK4 (Figure 7C) as well as USP9X and CDK4 in these samples (Figure 7D). We next assessed protein expression levels of KIFC2, USP9X, and CDK4 in 45 tumor samples from patients with HR^+^/HER2^–^ BC by IHC (Figure 7E). Consistently, there was a positive correlation in protein levels between KIFC2 and CDK4 (Figure 7F) as well as between USP9X and CDK4 (Figure 7G). Additionally, mRNA levels of *KIFC2* were positively correlated with expression levels of Ki-67 as determined by IHC staining (Figure 7, H and I) and with tumor size (Figure 7J) in patients with HR^+^/HER2^–^ BC in the FUSCC data set (27).

We next explored whether the expression levels of *KIFC2* were associated with the survival rate of patients with HR^+^/HER2^–^ BC who received adjuvant ET alone or in combination with CDK4/6 inhibitors in the FUSCC data set (27). The results showed that high expression of *KIFC2* was associated with a lower OS rate for patients who received adjuvant ET alone (excluding all chemotherapy) (Figure 7K) or in combination with the CDK4/6 inhibitor Palbo after ET resistance in the FUSCC data set (27) (Figure 7L).

Collectively, these results suggest that *KIFC2* is highly amplified in HR^+^/HER2^–^ BC and that its expression promotes growth and resistance to ET and CDK4/6 inhibitors in HR^+^/HER2^–^ BC by USP9X-mediated stabilization of CDK4 (Figure 7M).

## Discussion

Long-term recurrence and metastasis, as well as development of resistance to ET and/or CDK4/6 inhibitors, are the main problems in clinical management of HR^+^/HER2^–^ BC (27). Thus, it is imperative to identify molecular markers for predicting the responsiveness of patients to these therapies and to discover targets for improving therapeutic efficacy for patients with HR^+^/HER2^–^ BC (11). In this study, we report several interesting findings concerning the KIFC2-USP9X/CDK4 signaling axis in HR^+^/HER2^–^ BC growth and resistance to ET and CDK4/6 inhibitors.

First, *KIFC2* was highly amplified in HR^+^/HER2^–^ BC, and its high expression was associated with poor patient outcome and increased *TP53* mutation and pyrimidine metabolism. Chromosomal CNAs are frequently observed in various types of human cancers and can lead to deletion of tumor suppressors or amplification of oncogenes, thereby contributing to cancer initiation and progression (40, 41). In addition, some genes (such as *HER2*) affected by CNAs can serve as potential biomarkers and therapeutic targets for BC (42, 43). Thus, it is important to identify CNA-associated genes in BC progression. In this study, we report that *KIFC2* was amplified in approximately 50% of HR^+^/HER2^–^ BC samples, and its high expression was associated with poor patient prognosis (Figure 1 and Supplemental Figures 1 and 2). Interestingly, the *KIFC2* gene is located on chromosome 8q24.3 (21), a site associated with chromosomal gains and amplifications in a variety of cancers, including BC (44–46). Moreover, chromosomal 8q24.3–located genes are mainly overexpressed in human cancers due to increased copy number and are associated with unfavorable prognosis (44–46). Despite these facts, we cannot rule out the possibility that transcriptional or posttranscriptional mechanisms may also contribute to high expression of KIFC2 in HR^+^/HER2^–^ BC.

Second, the amplification of *KIFC2* was associated with increased *TP53* somatic mutation and pyrimidine metabolism (Supplemental Figures 5–8). Accumulating evidence shows that the interaction of genetic alterations is associated with distinct biological phenotypes and therapeutic responsiveness in BC (47). For instance, *TP53* mutations occur with *MYC* amplification in BC (48), and cooccurrence of *TP53* mutation and aurora kinase A (*AURKA*) amplification is associated with ET resistance in BC (47). Like *KIFC2* amplification (the present study), *TP53* mutations are also associated with resistance to ET (49) and CDK4/6 inhibitors (50) in HR^+^/HER2^–^ BC. Thus, whether there is a synergistic interplay between *KIFC2* amplification and *TP53* mutation in driving the growth and therapeutic resistance of HR^+^/HER2^–^ BC warrants further investigation. Additionally, it has been shown that activation of the pyrimidine metabolism pathway is required for cell proliferation and is intimately linked to progression and development of drug resistance in several kinds of human cancer (33, 34). In this study, we found that *KIFC2* amplification was associated with activation of the pyrimidine metabolism pathway (Supplemental Figure 7). Analogous to *KIFC2*, the proto-oncogene *MYCN* is amplified and promotes pyrimidine nucleotide biosynthesis in neuroblastoma cells (51). Blocking the pyrimidine metabolism pathway using antimetabolic chemotherapy drugs (such as capecitabine) may have therapeutic benefits for patients with *KIFC2*-amplified HR^+^/HER2^–^ BC.

Third, KIFC2 promoted growth and conferred resistance to ET and CDK4/6 inhibitors in HR^+^/HER2^–^ BC. Although dysregulation of KIFs has been linked to human cancers and certain KIFs are currently being validated as anticancer drug targets (12), information on the structure and function of KIFC2 remains relatively limited. Interestingly, it was recently documented that KIFC2 promotes progression and chemoresistance in prostate cancer by activating the NF-κB signaling pathway (26) and is a potential prognostic biomarker in colon and prostate cancers (24, 25). However, the functional and mechanistic role of KIFC2 in BC has not yet been investigated. In this study, we provide evidence that KIFC2 may act as a potential oncogene to accelerate growth and confer resistance to ET and CDK4/6 inhibitors in HR^+^/HER2^–^ BC (Figures 2 and 3 and Supplemental Figures 3 and 4). In clinical settings, it was also found that high *KIFC2* mRNA expression was associated with tumor growth and poor survival of patients with HR^+^/HER2^–^ BC who received ET alone or in combination with CDK4/6 inhibitors (Figure 7). Consistent with our screening results (Figure 1), *KIF14* has been shown to be amplified in BC and contribute to disease progression, chemoresistance, and poor patient prognosis (31, 52, 53). In addition, KIFC2 is a member of the kinesin 14 family, which includes other KIF proteins, such as kinesin family member C1 and kinesin family member C3 (54). Thus, whether some functions of KIFC2 overlap with those of other KIF14 family members needs to be explored.

Fourth, KIFC2 exerted its tumor-promoting and therapy-resistant functions in HR^+^/HER2^–^ BC by recruiting USP9X to stabilize CDK4. CDK4 is frequently overexpressed in BC and plays a crucial role in breast tumorigenesis (9, 11, 55). Moreover, its overexpression is associated with resistance to ET and CDK4/6 inhibitors (3, 56–59). Thus, it is essential to comprehend the mechanisms underlying regulation of CDK4 overexpression in human cancer. *CDK4* amplification is detected in about 15% of cases of sporadic BC (60). Additionally, recent studies demonstrated that CDK4 is targeted for proteasomal degradation by E3 ubiquitin-protein ligases, such as itchy E3 ubiquitin protein ligase (61), F-box only protein 8 (62), and S-phase kinase-associated protein 2 (63). Although several DUBs — such as ubiquitin-specific protease 51 (64) and DUB3 (65) — have been identified as targets of CDK4, the DUBs responsible for deubiquitination and stabilization of CDK4 are still unclear. In this study, we present evidence that USP9X acts as a DUB for CDK4, which interacts with and stabilizes CDK4. Moreover, KIFC2 stabilizes CDK4 by enhancing the interaction of CDK4 with USP9X (Figures 4 and 5 and Supplemental Figures 11 and 12). Notably, upregulation of KIFC2, USP9X, and CDK4 and a positive correlation in protein expression levels between KIFC2 and CDK4 as well as USP9X and CDK4 were noted in HR^+^/HER2^–^ BC tissues (Figure 7). Notably, reexpression of CDK4 in KIFC2-depleted cells partially rescued the decreased growth and increased sensitivity to Tam and CDK4/6 inhibitors caused by KIFC2 depletion both in vitro and in vivo (Figure 6 and Supplemental Figure 13).

It has shown that USP9X promotes survival, carcinogenesis, metastasis, and chemoresistance of BC by stabilizing its substrates, such as cell division cycle 123 (66), centriolar satellite protein CEP131 (67), and yes-associated protein 1 (68). Consequently, pharmacological inhibition of USP9X suppresses progression and enhances chemotherapy sensitivity in BC (69, 70). Interestingly, we discovered that depletion or pharmacological inhibition of USP9X enhanced cellular sensitivity to Tam and CDK4/6 inhibitors (Abema and Palbo) as single agents or in combination (Supplemental Figures 14 and 15). These findings indicate that targeting USP9X could be an effective strategy to overcome KIFC2-mediated resistance to ET and CDK4/6 inhibitors.

Collectively, the findings presented in this study elucidate functional and mechanistic roles for the KIFC2-USP9X/CDK4 signaling axis in promoting growth and resistance to ET and CDK4/6 inhibitors in HR^+^/HER2^–^ BC and highlight KIFC2 as a potential therapeutic target and predictive biomarker for therapeutic responsiveness in these patients.

## Methods

### Sex as a biological variable.

All mice used in this study were female because BC is primarily relevant in females.

### Study cohorts.

Our study used data from 3 patient cohorts. The FUSCC cohort included 318 participants who had undergone surgery at the Cancer Center (99.7% female, 0.3% male). Tissue samples collected from these participants were analyzed for CNAs, RNA-Seq, somatic mutations, and metabolomics. Data for the TCGA and METABRIC cohorts were obtained from publicly available databases and analyzed for external validation. The TCGA cohort consisted of 610 patients with RNA-Seq and CNA data, of which 99.2% were female and 0.8% male. The METABRIC cohort included 1,217 patients, all of whom were female, with available RNA-Seq and CNA data.

### Cell culture and chemicals.

The human HR^+^/HER2^–^ BC cell lines (MCF7 and T47D) (32) and HEK293T cells were sourced from the Cell Bank of Chinese Academy of Sciences. Cells were cultured in DMEM medium (Sangon Biotech, E600003) with 10% FBS (Gibco, 10270-106). The TamR and its parental MCF7 cell lines (71) were provided by Cuixia Yang (Shanghai Jiao Tong University School of Medicine, Shanghai, China). The PalboR and its parental MCF7 cell lines were purchased from MEISEN CELL. MCF7-TamR and MCF7-PalboR cell lines were cultured in DMEM in the presence of 1 μM Tam and 1 μM Palbo, respectively. All cells were treated with mycoplasma elimination reagent (Yeasen Biotech, 40607ES01) and authenticated via short tandem repeat (STR) profiling analysis. Information concerning the chemicals used in this study is provided in Supplemental Table 2.

### Construction of expression vectors.

cDNAs of *KIFC2*, *CDK4*, and *TP53* were amplified by PCR with the indicated primers and ligated into pLVX-yu-2*Flag-3C or pLVX-IRES-NEO vector to generate Flag-KIFC2, HA-CDK4, Flag-CDK4, and HA-p53 expression vectors, respectively. Point mutations of *TP53* were generated by PCR-based mutagenesis. The pCMV-3×FLAG-USP9X (human)–Neo and pCMV-3×FLAG-USP9X (human)–C1566S-Neo expression vectors were synthesized by MIAOLING Bio. The pEF-DEST51-V5-USP9X was provided by Stephen A. Wood (Griffith University, Brisbane, Queensland, Australia) (70). The shRNAs targeting *KIFC2*, *CDK4*, and *USP9X* were designed using Block-iT RNAi Designer from Invitrogen and then cloned into a pLKO.1-TRC vector. The shRNA construct targeting *TP53* was provided by Xiang Zhou (FUSCC). The primers are listed in Supplemental Table 3, and the shRNA sequences are listed in Supplemental Table 4.

### Plasmid transfection and lentiviral infection.

Plasmid transient transfection was performed using Tenfect DNA transfection reagents (TEYE Biotech, FT19301) following the protocol provided by the manufacturer. For generation of stable cell lines, the specified lentiviral vectors were cotransfected with packaging plasmids into HEK293T cells. Viral supernatants were collected and used to infect the indicated cells with polybrene (MilliporeSigma, H9268). Two days later, puromycin or G418 was used for drug selection for 2 weeks. Immunoblotting confirmed overexpression or knockdown efficiency.

### Immunoblotting and IP assays.

Cell lysates were prepared using RIPA buffer with protease/phosphatase inhibitors (Bimake, B14002 and B15003) and analyzed by SDS-PAGE and immunoblotting. PVDF membranes (MilliporeSigma, IPVH00010) were blocked with 5% BSA (Yeasen, 36101ES80) and incubated with primary antibodies at 4°C overnight, followed by HRP-conjugated secondary antibodies. A chemiluminescence substrate kit (Tanon, 180-5001E) was used for detection of protein signals. For IP assays, cells were collected using NP-40 lysis buffer. Cellular lysates were then incubated with anti-HA beads (Shanghai Genomics Tech, GNI4510-HA) or anti-Flag beads (Bimake, B23102) for at least 3 hours. In other cases, cellar lysates were incubated overnight at 4°C with the indicated primary antibodies on a rotating platform, then incubated with protein A/G magnetic beads (Bimake, B23202) for 3 hours. The beads were washed 3 times and analyzed with immunoblotting. Detailed information on antibodies is presented in Supplemental Table 5.

### RNA isolation and RT-qPCR.

Total RNA isolation was performed with Trizol reagent (Takara, 9109), followed by cDNA synthesis using PrimeScript RT Master Mix (Vazyme, R323-01). Quantitative PCR analysis was conducted on the Eppendorf Realplex system with ChamQ SYBR Master Mix (Vazyme, Q711), employing custom-designed primers (sequences in Supplemental Table 6).

### IF staining.

Cells were fixed, permeabilized, and blocked with BSA, followed by overnight incubation with primary antibodies at 4°C. After washing, Alexa 488/555–conjugated secondary antibodies and DAPI (Abcam, ab104139) were used for staining. Images were acquired using a Leica fluorescence microscope.

### Cell-cycle analysis.

After fixation with 70% ethanol for 2 hours, cells were washed with PBS, stained with a cell-cycle analysis kit (Yeasen Biotech, 40301ES50), and then analyzed by flow cytometry.

### CCK-8 assays.

Cells were seeded into 96-well plates. After appropriate incubation, cells were treated with CCK-8 solution (Yeasen, 40203ES92). After incubation at 37°C for 1–4 hours, OD_450_ was measured.

### Cell viability and drug sensitivity assays.

For cell viability assays, cells were cultured in 96-well plates and treated with vehicle or with Tam, Abema, Palbo, and capecitabine. OD_450_ was detected with the CCK-8 kit. For colony formation assays, cells were plated into 6- or 12-well microplates for 2 days and treated as indicated. Surviving colonies were fixed with methanol and stained with crystal violet for 30 minutes.

### IC_50_ calculations.

As described previously (72, 73), IC_50_ values were determined by GraphPad Prism 9 using a 3-parameter nonlinear regression model (log[inhibitor] versus response). IC_50_ was calculated from the logIC_50_ value by exponentiation. The 3 fitting parameters, including bottom, top, and logIC_50_, were used to calculate the potential differences between 2 groups in baseline response, maximal drug effect, and potency. The extra-sum-of-squares *F* test was used to assess the statistical significance of the differences between the groups (74, 75). To provide a more comprehensive statistical analysis, we also analyzed 95% CIs for the IC_50_ values.

### LC-MS/MS analysis.

HEK293T cells expressing pLVX or Flag-KIFC2 were lysed using NP-40 buffer and immunoprecipitated with anti-Flag beads. SDS-PAGE gels were stained with Coomassie brilliant blue solution and then subjected to LC-MS/MS analysis at the Center for Proteomics (Institutes of Biomedical Sciences, Fudan University). MCF7 cells stably expressing shNC or sh*KIFC2* were digested with trypsin (BasalMedia, S310KJ) and counted. The same number of cells were collected for LC-MS/MS–based metabolomics at Biotree Biotech to identify differentially expressed metabolites.

### Ubiquitination assays.

HEK293T cells were transfected with the specified expression plasmids for 48 hours and cultured with 10 μM MG-132 for 6 hours, followed by ubiquitination analysis according to the protocol as described previously (76).

### PDOs.

The PDOs were derived from patients with HR^+^/HER2^–^ BC in FUSCC using previously described methods (77). Briefly, fresh tissues were minced and digested with collagenase (MilliporeSigma, C9407) and hyaluronidase (MilliporeSigma, 37326-33-3) at 37°C for 1–3 hours. After being filtered with a 100 μm filter membrane, the digested tissue was centrifuged. Red blood cells were removed using lysis buffer. Centrifuged organoid pellets were resuspended in 1 mL basement membrane extract type 2 (Trevigen, 3533-010-02) and plated into 384-well plates (Greiner, GN781900), followed by drug treatment for 5–7 days. Morphological changes were documented by microscopy, and cell viability was quantified using the CellTiter-Glo 3D assays (Promega, G9683).

### IHC staining.

IHC staining was carried out as described previously (78) using an anti-CDK4, anti–Ki-67, anti-KIFC2, or anti-USP9X antibody. Expression levels of CDK4, Ki-67, KIFC2, and USP9X were quantified using the histochemistry score (H-score), which was calculated by multiplying the percentage of cells at each staining intensity level by a corresponding factor, including weak intensity (×1), moderate intensity (×2), and strong intensity (×3). The H-score ranged from 0 to 300, with higher values indicating stronger staining intensity and greater overall positivity.

### Xenograft tumor models.

To assess tumorigenic potential, 5 × 10^6^ MCF7 cells were diluted in PBS and mixed with an equal volume of Matrigel (Corning Falcon, 356334), then injected into the mammary fat pad of female BALB/c nude mice. Estradiol-17β pellets (Innovative Research of America, SE-121) were implanted subcutaneously. Therapeutic interventions were initiated when tumor volumes reached 50–100 mm³: Tam (50 mg/kg, dissolved in corn oil, daily intraperitoneal injection) and Abema (25 mg/kg, dissolved in corn oil, every-other-day oral gavage). Tumor diameters were measured and tumor volumes were calculated using the formula 0.5 × (length) × (width)². Mice were sacrificed once they developed cachexia or lost 10% of their body weight.

### CNA annotation and DEG identification.

CNA events were defined by GISTIC 2.0 (79) based on discrete copy number calls. The values –2, –1, 0, 1, and 2 correspond to homozygous deletion, single copy deletion, diploid normal copy, low-level copy number amplification, and high-level copy number amplification, respectively (28, 29). The genes differentially expressed (KIFs) between HR^+^/HER2^–^ BC tissues and normal breast specimens were defined with DESeq2 (80) according to the following criteria: absolute value of log_2_FC (tumor/normal) > 0.58 and *P* < 0.05 (81).

### Statistics.

Triplicate biological replicates were performed for all experiments. Data analysis was conducted using R, GraphPad Prism 9, and ImageJ (NIH). Statistical tests included 2-tailed Student’s *t* tests (paired or unpaired) or Mann-Whitney *U* tests for comparisons between 2 groups and 1-way ANOVA for comparisons among multiple groups. For growth curves and dose-dependent colony formation assays, statistical comparisons between groups were conducted only at the final time point or highest drug concentration using 2-tailed Student’s *t* tests or 1-way ANOVA, as appropriate. Kaplan-Meier curves with log-rank testing was used for survival analysis. A *P* value less than 0.05 was considered significant.

### Study approval.

All HR^+^/HER2^–^ BC specimens and their paired adjacent normal samples were collected from patients who underwent surgery at FUSCC. The study was conducted using procedures approved by the Medical Ethics Committee of FUSCC, and written informed consent was received from all participants. Clinical and multi-omics data for patients with HR^+^/HER2^–^ BC from the FUSCC cohort have been described previously (27). Additionally, all animals used in this study were centrally purchased through the Animal Experimental Center of FUSCC. All animal procedures were approved by the Institutional Animal Care and Use Committee of FUSCC.

### Data availability.

The data presented in this study are available within the article text and figures. Values for all data points in graphs are reported in the Supporting Data Values file. The clinical information of this study is available upon reasonable request.

## Author contributions

FLZ, XJ, AYC, and DQL designed and supervised the project. SYY performed the experiments. SYY, MLJ, LA, QZ, YXL, and CJL contributed to data analysis. MYH, JYC, and YLZ performed the software analysis. SYY and MLJ curated the data. ZMS and XH provided resources. SYY and MLJ wrote the original draft. FLZ, XJ, AYC, and DQL reviewed and edited the manuscript. SYY and MLJ contributed equally to this work and share first authorship. The order of authorship was determined based on their overall contributions to the project.

## Supplementary Material

Supplemental data

Unedited blot and gel images

Supporting data values

## Version 1 04/29/2025

In-Press Preview

## Version 2 06/16/2025

Electronic publication
