# Supplementary material for: Kinesin-like protein KIFC2 stabilizes CDK4 to accelerate growth and confer resistance in HR+/HER2– breast cancer
Source: J Clin Invest. 2025 Apr 29;135(12):e183531. doi: 10.1172/JCI183531 (PMC12165803; doi:10.1172/JCI183531)
Supplement: Supplemental data [file jci-135-183531-s138.pdf]

Supplemental information for

Yang SY, et al. Kinesin-like protein KIFC2 stabilizes CDK4 to accelerate growth and confer resistance in HR+/HER2- breast cancer.

This Supplemental information contains:

Supplemental Figures: 16

Supplemental Tables: 6

Supplemental figures and figure legends

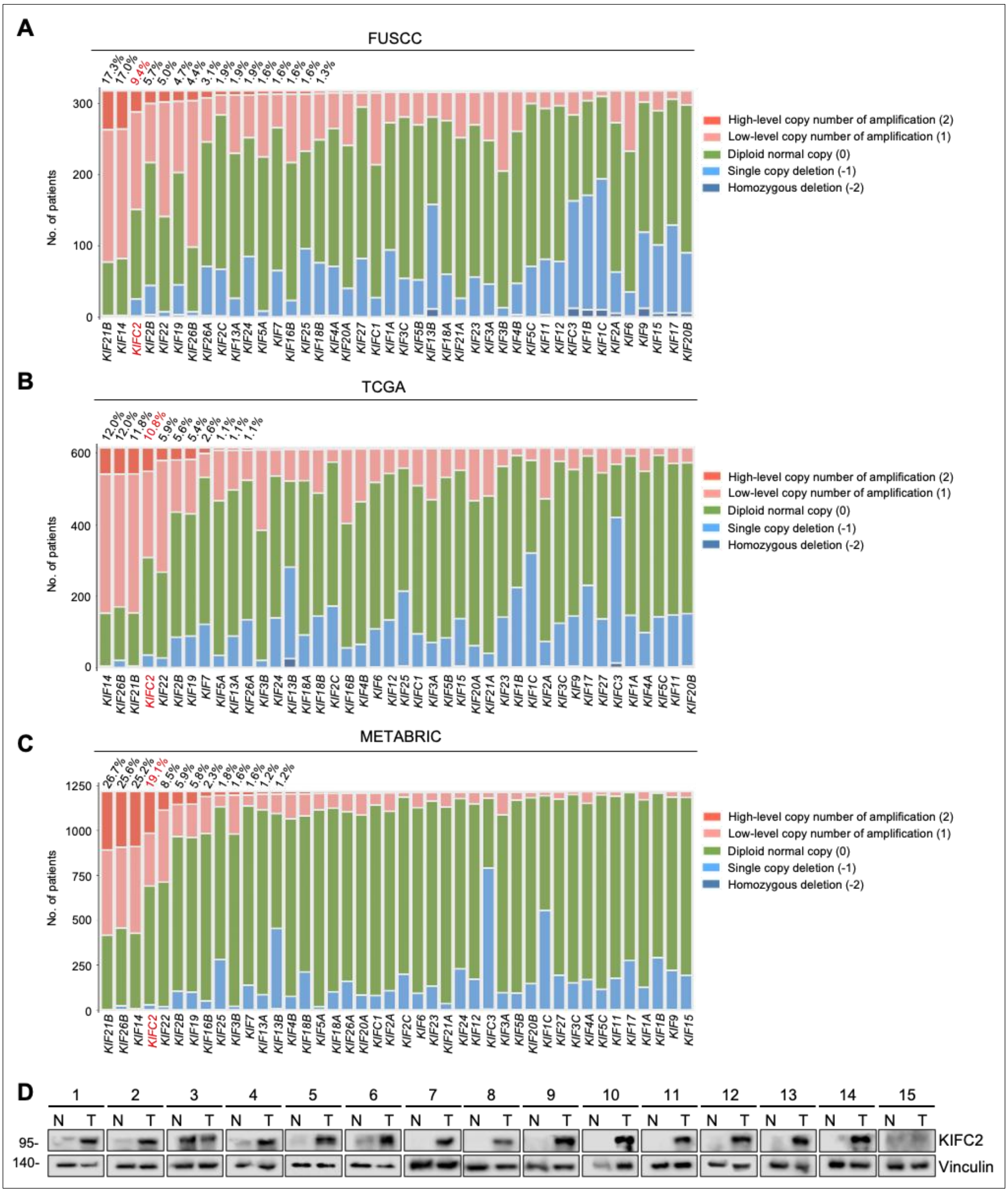

Supplemental Figure 1. Overview of the CNA status of the KIF family members in HR+/HER2- BC from the FUSCC, TCGA, and METABRIC datasets.

(**A-C**) The CNA status of the KIF family members in HR+/HER2- BC from the FUSCC (A), TCGA (B), and METABRIC (C) datasets. The CNA events were defined by GISTIC 2.0 based on discrete copy number calls. The values of -2, -1, 0, 1, and 2 represent homozygous deletion, single copy deletion, diploid normal copy, low-level copy number amplification, and high-level

copy number amplification, respectively. The HR+/HER2- BC cohorts from the FUSCC, TCGA, and METABRIC include 318, 610, and 1217 patients, respectively.

(**D**) Immunoblotting analysis of KIFC2 protein levels in 15 pairs of HR+/HER2- BC tissues and matched non-cancerous samples. The corresponding quantitative results are shown in Figure 1H.

No. of patients, number of patients; KIF, Kinesin family; HR, hormone receptor; HER2, human epidermal growth factor receptor 2; CNA, copy number alternation.

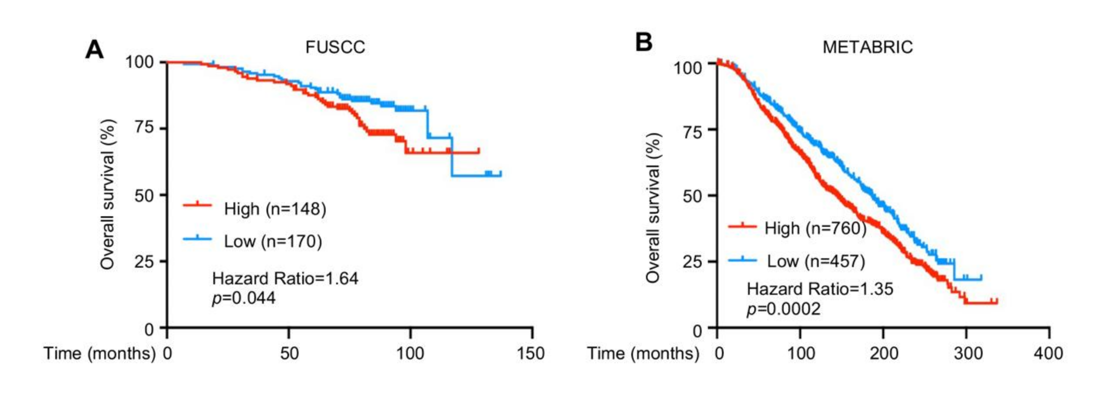

Supplemental Figure 2. High expression of *KIFC2* is associated with poor prognosis of HR+/HER2- BC patients.

(**A-B**) Kaplan-Meier analysis of overall survival (OS) in the HR+/HER2- BC patients from the FUSCC (A) and METABRIC (B) datasets, stratified by KIFC2 expression levels. Log-rank test.

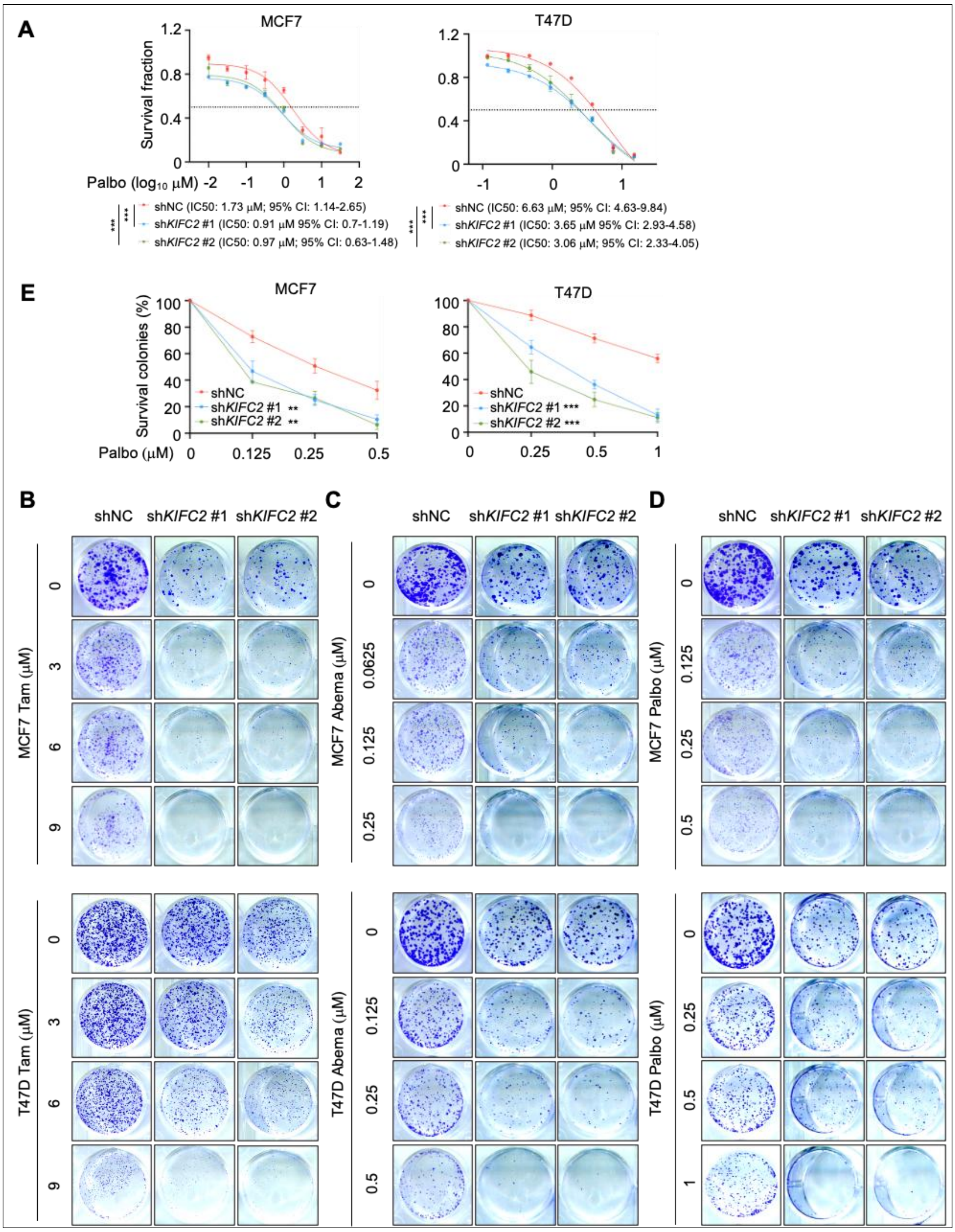

Supplemental Figure 3. Knockdown of KIFC2 enhances the sensitivity of HR+/HER2- BC cells to tamoxifen and CDK4/6 inhibitors in vitro.

(**A**) MCF7 and T47D cells stably expressing shNC or sh*KIFC2* (#1 and #2) were treated with or without increasing concentrations of Palbo for 72 h and subjected to CCK-8 assays to evaluate IC50 values.

(**B and C**) MCF7 and T47D cells stably expressing shNC or sh*KIFC2* (#1 and #2) were subjected to clonogenic survival assays in the presence or absence of increasing concentrations of Tam (B) or Abema (C) for 7-9 days. The representative images of the survival colonies are shown. The corresponding quantitative results are shown in Figure 3, C and D, respectively.

(**D and E**) MCF7 and T47D cells stably expressing shNC or sh*KIFC2* (#1 and #2) were subjected to clonogenic survival assays in the presence or absence of increasing concentrations of Palbo for 7-9 days. The representative images of the survival colonies (D) and the corresponding quantitative results (E) are shown.

Data are mean ± SD (**A** and **E**) (n = 3 per group).

Statistical analysis: (**A**) extra-sum-of-squares F test; (**E**) one-way ANOVA.

Tam, tamoxifen; Abema, abemaciclib; Palbo, palbociclib.

**, p<0 .01; ***, p<0 .001.

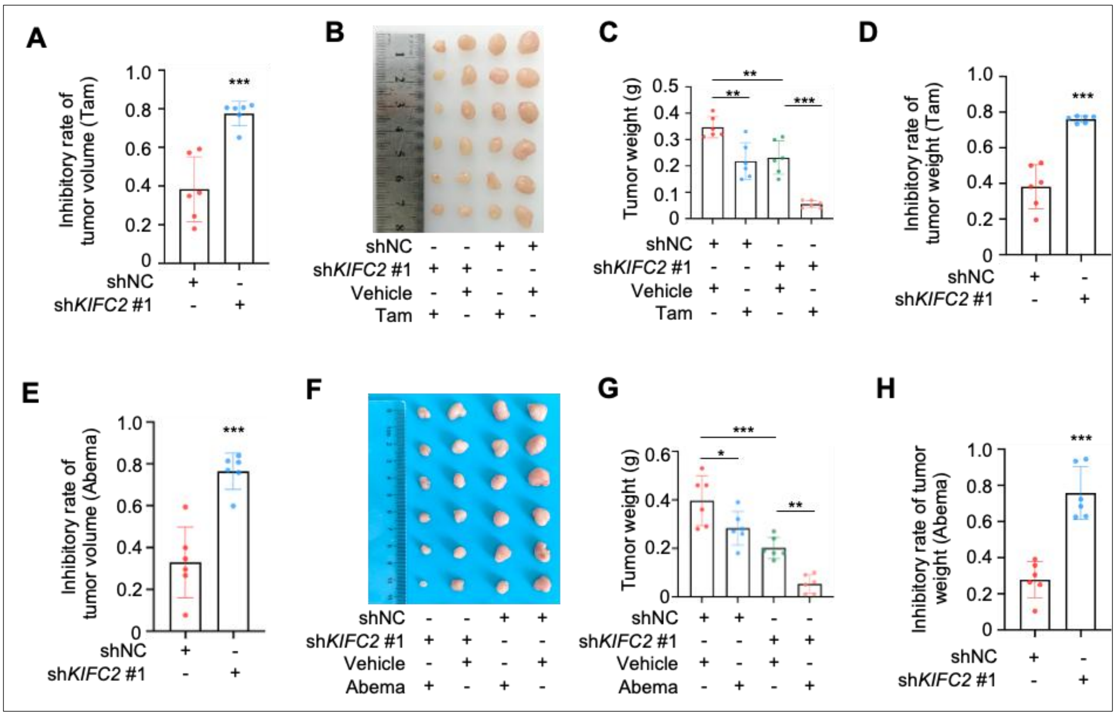

Supplemental Figure 4. Knockdown of KIFC2 enhances the sensitivity of HR+/HER2- BC cells to tamoxifen and CDK4/6 inhibitors in vivo.

(**A-D**) MCF7 cells stably expressing shNC or sh*KIFC2* #1 were injected into the mammary fat pad of BALB/c female nude mice. After 18 days of injection, mice were treated with vehicle or Tam. The inhibitory rate of tumor volume (A), xenograft tumors (B), tumor weight (C), and inhibition rate of tumor weight (D) are shown.

(**E-H**) MCF7 cells stably expressing shNC or sh*KIFC2* #1 were injected into the mammary fat pad of BALB/c female nude mice. After 18 days of injection, mice were administered with vehicle or Abema. The inhibitory rate of tumor volume (E), xenograft tumors (F), tumor weight (G), and inhibition rate of tumor weight (H) are shown.

Data are mean ± SD (**A, C, D, E, G,** and **H**) (n = 6 per group).

Statistical analysis: (**A, D, E,** and **H**) two-tailed Student's *t* test; (**C** and **G**) one-way ANOVA.

Tam, tamoxifen; Abema, abemaciclib.

*, *p*<0 .05; **, *p*<0 .01; ***, *p*<0 .001.

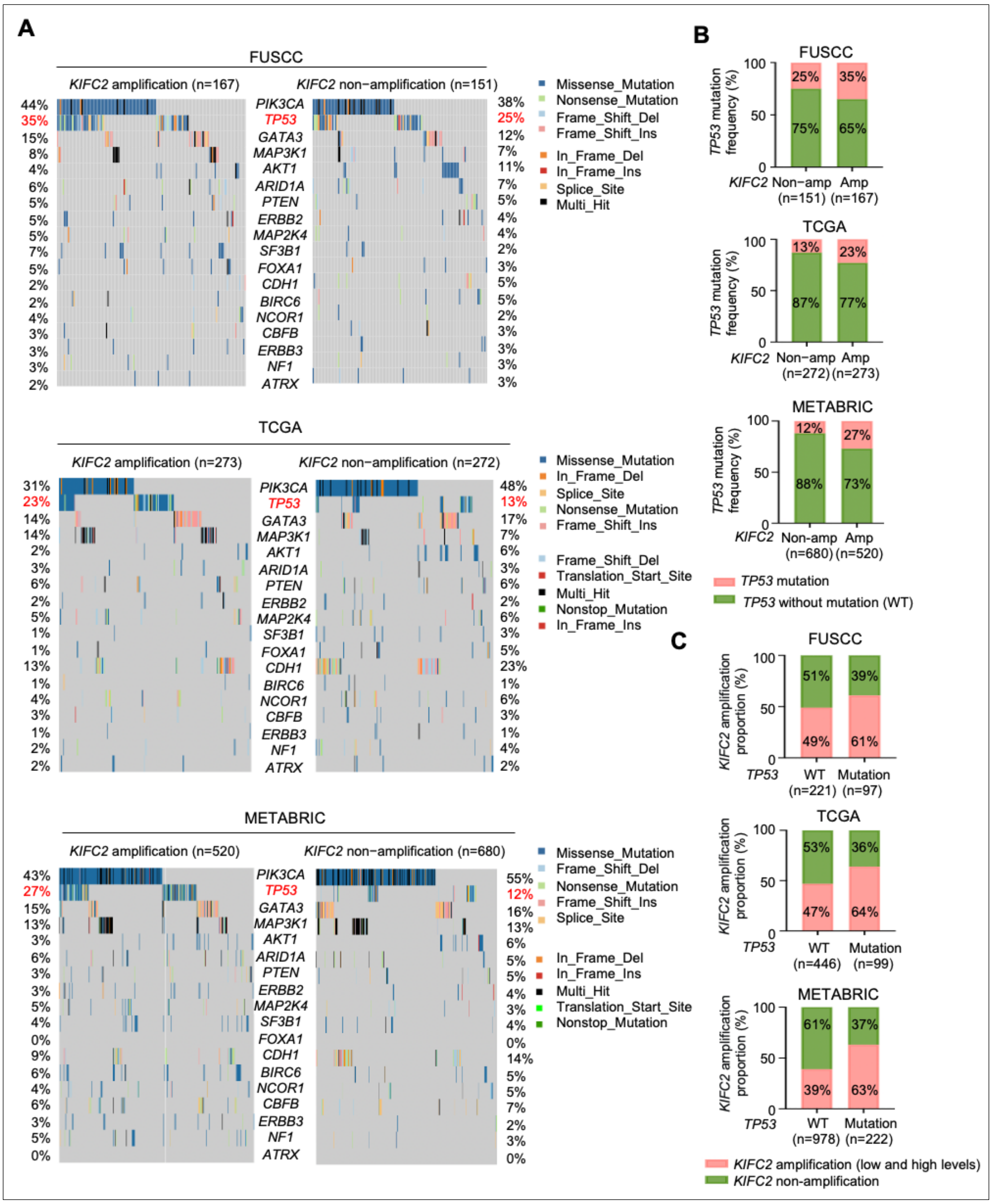

Supplemental Figure 5. *KIFC2* amplification is associated with increased *TP53* somatic mutations.

(**A**) The differences in the somatic mutational profiles of HR+/HER2- BC samples with or without *KIFC2* amplification in the FUSCC, TCGA, and METABRIC datasets.

(**B**) *TP53* mutation frequency (%) in HR+/HER2- BC samples with or without *KIFC2* amplification in the FUSCC, TCGA, and METABRIC datasets.

(**C**) The proportion of *KIFC2* amplification (%) in HR+/HER2- BC samples with or without *TP53* mutations in the FUSCC, TCGA, and METABRIC datasets.

HR, hormone receptor; HER2, human epidermal growth factor receptor 2; CNA, copy number alternation; Non-amp, non-amplification; Amp, amplification; *TP53*, tumor protein p53.

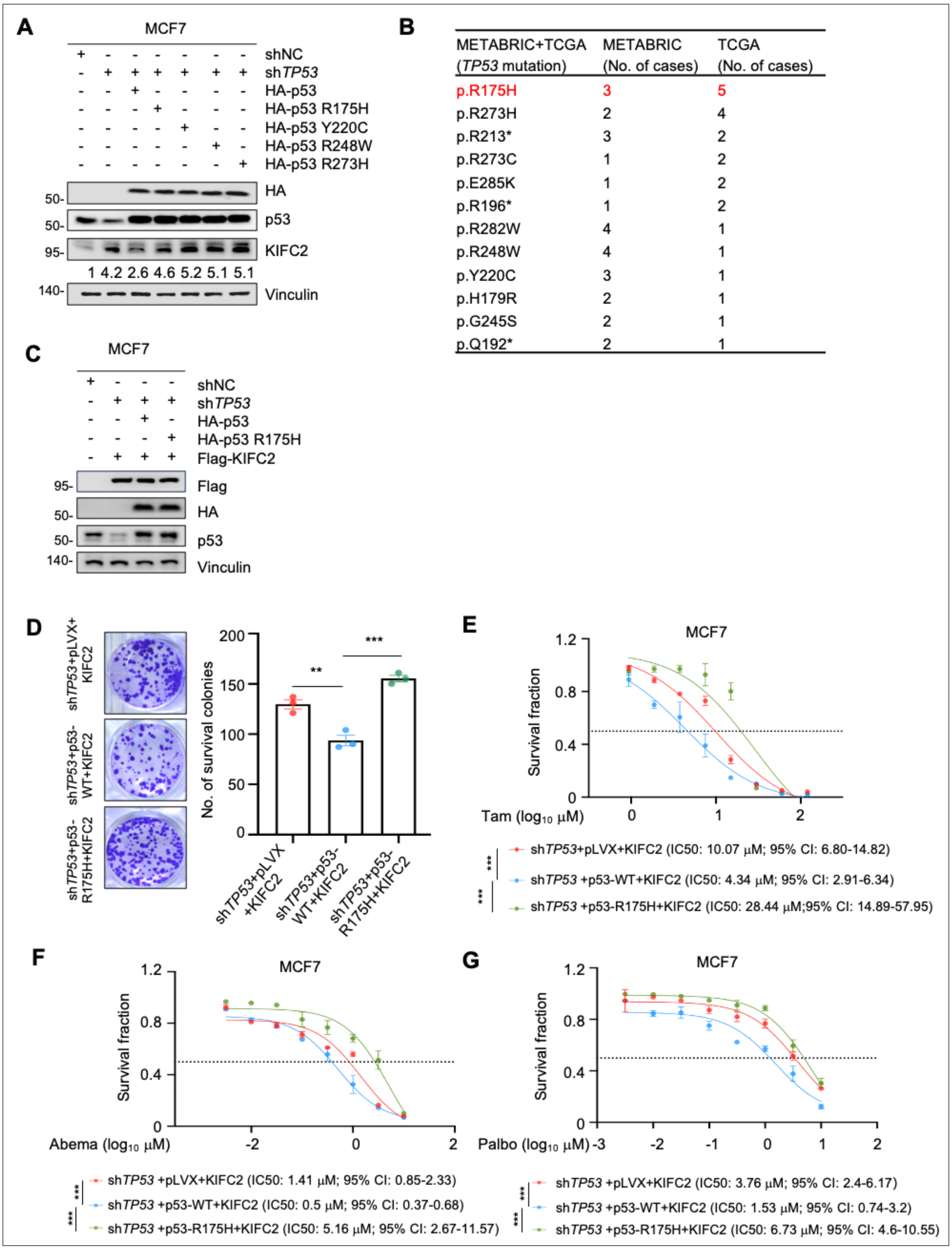

Supplemental Figure 6. KIFC2 has a differential role in promoting HR+/HER2- BC progression and therapeutic resistance under different genetic backgrounds of p53.

(**A**) Immunoblotting analysis of KIFC2 protein levels in p53-depleted MCF7 cells with re-expression with wild-type (WT) p53 or several most reported mutant p53 variants (R175H, Y220C, R248W, and R273H).

(**B**) Analysis of the most common *TP53* mutations in HR+/HER2- BC patients in the METABRIC and TCGA databases.

(**C**) p53-depleted MCF7 cells were infected with Flag-KIFC2 alone or in combination with wild-type p53 or p53-mutant R175H. Immunoblotting assays were performed to assess the expression levels of p53 and KIFC2.

(**D**) Cells shown in panel C were subjected to colony formation assays.

(**E-G**) Cells shown in panel C were treated with or without increasing concentrations of Tam (E), Abema (F), and Palbo (G) for 72 h and subjected to CCK-8 assays to evaluate IC50 values.

Data are mean ± SD (**D-G**) (n = 3 per group).

Statistical analysis: (**D**) one-way ANOVA; (**E-G**) extra-sum-of-squares F test.

No. of cases, number of cases; *TP53,* tumor protein p53; Tam, tamoxifen; Abema, abemaciclib; Palbo, palbociclib.

, *p*<0 .01; *, *p*<0 .001.

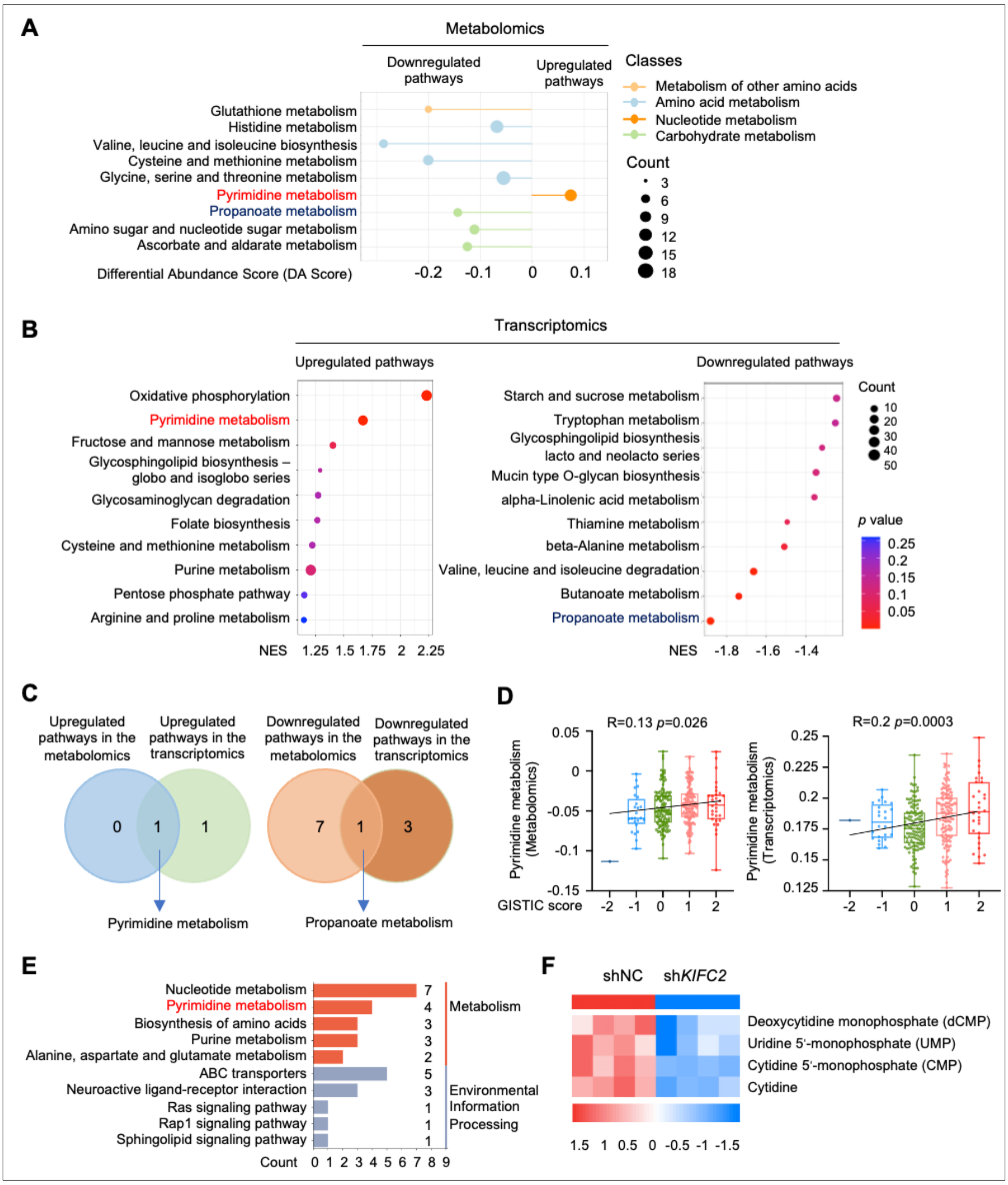

Supplemental Figure 7. *KIFC2* amplification is associated with active pyrimidine metabolism.

(**A**) Analysis of the impact of *KIFC2* amplification on the upregulated and downregulated metabolic pathways using metabolomics datasets from the FUSCC cohort.

(**B**) Analysis of the impact of *KIFC2* amplification on the upregulated and downregulated metabolic pathways using transcriptomics datasets from the FUSCC cohort.

(**C**) Cross-analysis of the altered metabolic pathways in both metabolomics (A) and transcriptomics (B) datasets from the FUSCC cohort.

(**D**) Spearman correlation analysis of the relationship between pyrimidine metabolism and *KIFC2* amplification. The center line represents the median.

(**E**) The KEGG pathway enrichment analysis of the metabolites that were downregulated upon KIFC2 knockdown.

(**F**) Heatmap showing the downregulation of pyrimidine metabolites in KIFC2-knockdown versus control cells.

NES, normalized enrichment score; KEGG, Kyoto Encyclopedia of Genes and Genomes.

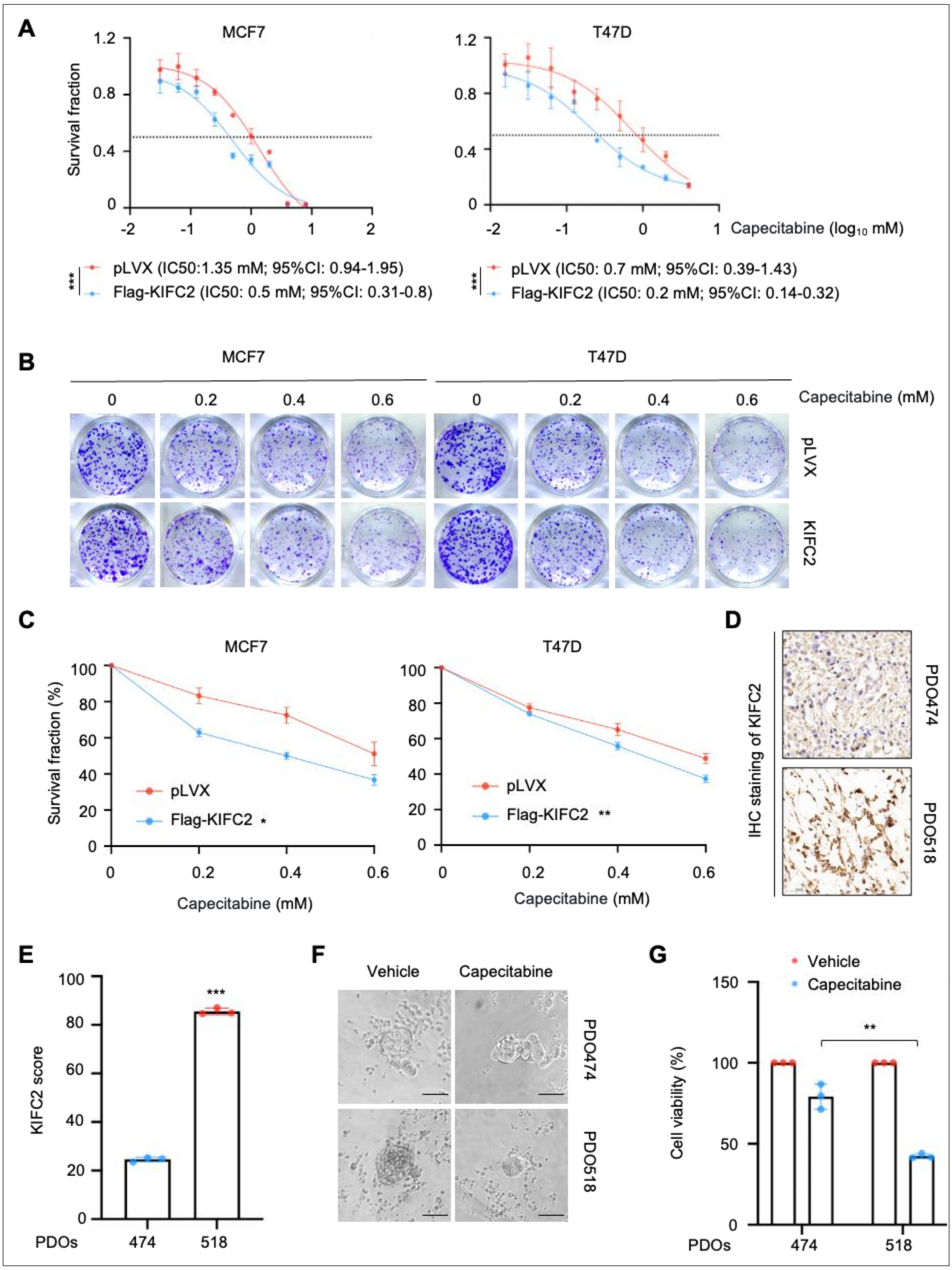

Supplemental Figure 8. KIFC2-overexpressing cells increase sensitivity to the antimetabolite chemotherapy agent capecitabine.

(**A**) MCF7 and T47D cells stably expressing pLVX or Flag-KIFC2 were treated with or without increasing concentrations of capecitabine for 72 h and then subjected to CCK-8 assays to evaluate IC50 values.

(B and C) MCF7 and T47D cells stably expressing pLVX or Flag-KIFC2 were subjected to clonogenic survival assays in the presence or absence of increasing concentrations of capecitabine for 7-9 days. The representative images of the survival colonies are shown in B and the corresponding quantitative results are shown in C.

(**D and E**) The expression status of KIFC2 in two HR+/HER2- BC PDOs was assessed by IHC staining of postoperative pathological tissue slices from the same patients. Representative images (D) and the corresponding quantitative results (E) are shown. Scale bar: 50 μm.

(**F and G**) CellTiter-Glo 3D cell viability assays were performed in two HR+/HER2- BC PDOs treated without or with capecitabine (20 μM). Representative images of the organoids after drug treatment (F) and the corresponding quantitative results (G) are shown. Scale bar: 100 μm.

Data are mean ± SD (**A, C, E,** and **G**) (n = 3 per group).

Statistical analysis: (**A**) extra-sum-of-squares F test; (**C, E,** and **G**) two-tailed Student's *t* test.

PDO, patient-derived organoid.

*, p<0 .05; **, p<0 .01; ***, p<0 .001.

A

Proteins	No. of identified unique peptides	Peptide sequences
PPP2CB<br>(Protein phosphatase 2 catalytic subunit beta)	7	GAGYTFGQDISETFNHANGLTLVSR<br>GGWGISPR<br>QLNENQVR<br>RGEPHVTRR<br>SPDTNYLFMGDYVDR<br>YGNANVWK<br>YSFLQFDPAPR
E2F5<br>(E2F transcription factor 5)	5	AAADTLAVR<br>ESSSSKPVVFPVPPPDDLTQPSSQSLTPVTPQK<br>NVMDDSINNR<br>SHSGPIHVLLINK<br>SLGLLTTK
CDK4<br>(Cyclin-dependent kinase 4)	4	ALQHSYLHKDEGNPE<br>GPRPVQSVVPEMEESGAQLLLEMLTFNPHKR<br>RLEAFEHPNVVR<br>VTLVFEHVDQDLR
YWHAG<br>(Tyrosine 3-monooxygenase/tryptophan 5-monooxygenase activation protein gamma)	3	NVTELNEPLSNEER<br>TAFDDAIAELDTLNEDSYKDSTLIMQLLR<br>YLAEVATGEK
TFDP1<br>(Transcription factor Dp-1)	3	GVVSLVAVHPSTVNPLGK<br>QSQLQELILQQIAFK<br>VFIDQNLSPGK

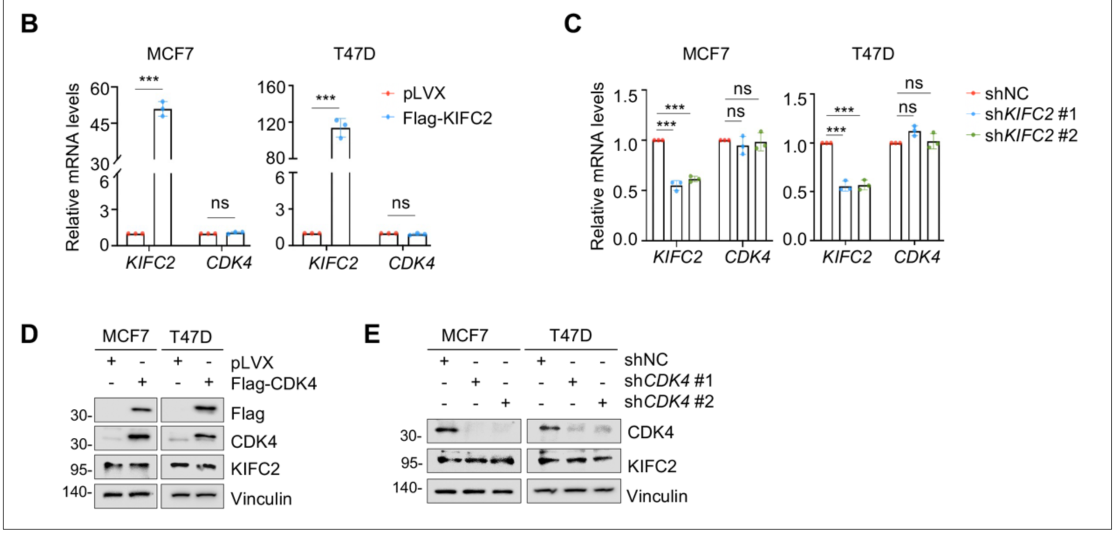

Supplemental Figure 9. KIFC2 regulates CDK4 at the protein but not mRNA levels.

(**A**) The 5 cell-cycle related proteins among 165 KIFC2-interacting proteins identified by IP coupled with LC-MS/MS assays.

(**B-C**) RT-qPCR analysis of the mRNA levels of *CDK4* and *KIFC2* in MCF7 and T47D cells with ectopic expression (B) or knockdown (C) of KIFC2.

(**D and E**) Immunoblotting analysis of KIFC2 protein levels in MCF7 and T47D cells with ectopic expression (D) or knockdown (E) of CDK4.

Data are mean ± SD (**B** and **C**) (n = 3 per group).

Statistical analysis: (**B**) two-tailed Student's *t* test; (**C**) one-way ANOVA.

No. of identified unique peptides, number of identified unique peptides.

***, p<0 .001; ns, non-significant.

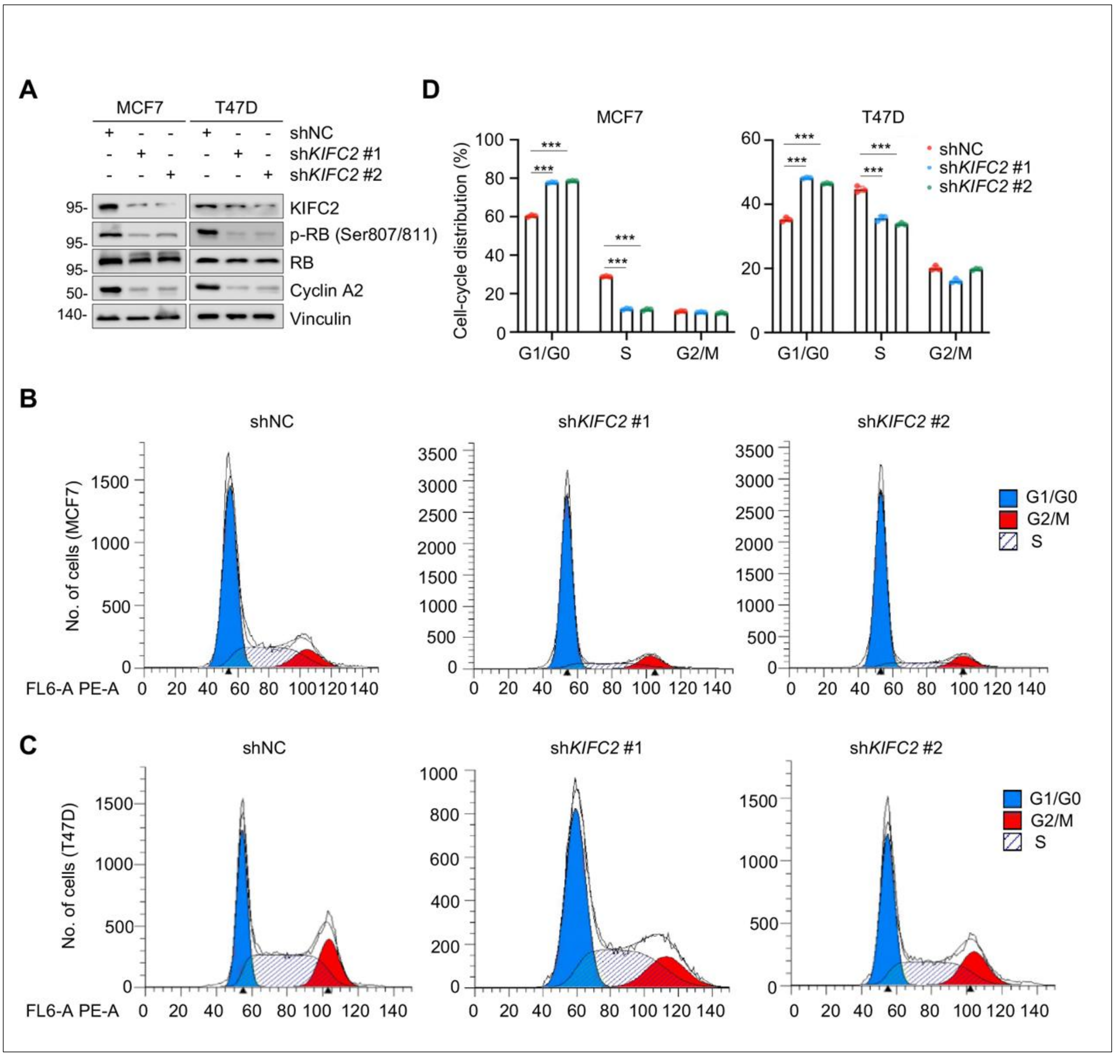

Supplemental Figure 10. KIFC2 promotes the transition from the G1 to S phase of the cell cycle.

(**A**) Immunoblotting analysis of phosphorylated RB at serines 807/811 (p-RB Ser807/811), total RB, and CyclinA2 in MCF7 and T47D cells stably expressing shNC or sh*KIFC2* (#1 and #2).

(**B-D**) FACS analysis of cell-cycle distributions in MCF7 and T47D cells stably expressing shNC or sh*KIFC2* (#1 and #2). The representative FCAS images (B and C) and the corresponding quantitative analysis (D) are shown. one-way ANOVA test.

Data are mean ± SD (D) (n = 3 per group).

***, p<0 .001.

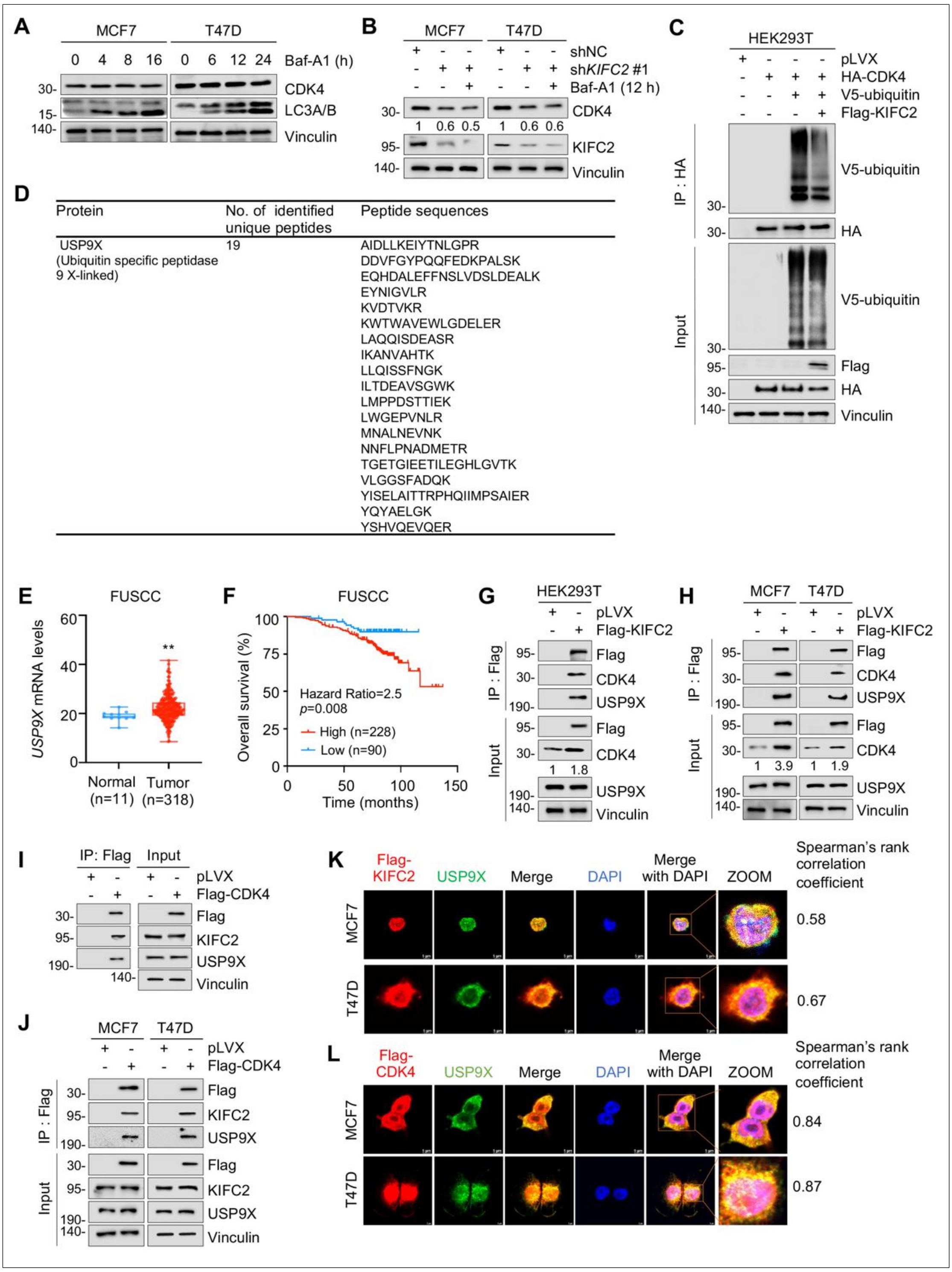

Supplemental Figure 11. Identification of USP9X as a DUB for CDK4 protein stability.

(**A**) MCF7 and T47D cells were treated with or without 200 nM Baf-A1 for the indicated times, followed by immunoblotting assays with the indicated antibodies. The quantitation of the relative expression levels of CDK4 was performed using the ImageJ software.

(**B**) MCF7 and T47D cells stably expressing shNC or sh*KIFC2* #1 were treated with or without 200 nM Baf-A1 for 12 h, followed by immunoblotting assays with the indicated antibodies.

(**C**) HEK293T cells were transfected with pLVX, Flag-KIFC2, HA-CDK4, or V5-ubiquitin alone or in combination. Cells were then treated with or without MG-132 for 6 h, followed by sequential IP and immunoblotting assays.

(**D**) The identified peptide sequences for USP9X by LC-MS/MS assays.

(**E**) Analysis of the mRNA levels of *USP9X* in HR+/HER2- BC dataset from the FUSCC cohort. The center line represents the median.

(**F**) Kaplan-Meier analysis of overall survival of HR+/HER2- BC patients with high or low expression levels of *USP9X* in the FUSCC dataset.

(**G-J**) HEK293T (G and I) cells were transfected with the indicated expression vectors for 48 h, while MCF7 and T47D (H and J) cells were infected with Flag-KIFC2, followed by sequential IP and immunoblotting analyses.

(**K and L**) Immunofluorescent staining was carried out to examine the colocalization of Flag-KIFC2 (red) and USP9X (green) (K) as well as Flag-CDK4 (red) and USP9X (green) (L) in MCF7 and T47D cells. Scale bar: 5 μm.

Statistical analysis: (**E**) Mann-Whitney U tests; (**F**) Log-rank test.

Baf-A1, bafilomycin A1; No. of identified unique peptides, number of identified unique peptides.

**, p<0 .01.

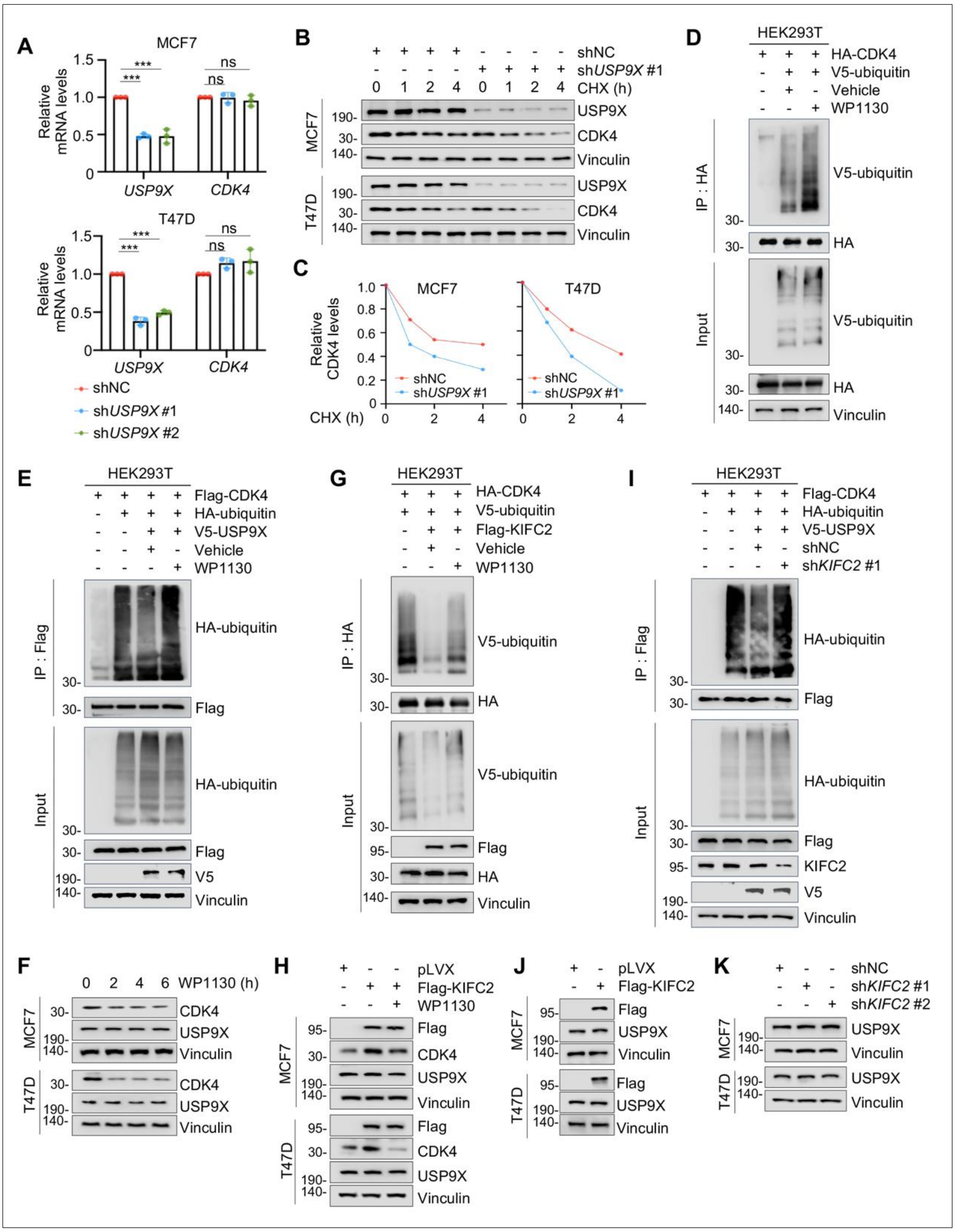

Supplemental Figure 12. KIFC2 recruits USP9X to stabilize CDK4.

(**A**) RT-qPCR analysis of the mRNA levels of *CDK4* and *USP9X* in MCF7 and T47D cells expressing shNC or sh*USP9X* (#1 and #2). One-way ANOVA test.

(**B and C)** Cells with stable shNC or sh*USP9X* #1 expression were treated with or without CHX, followed by immunoblotting analysis.

(**D and E**) HEK293T cells were transfected with the indicated expression vectors for 40 h, followed by treatment with or without 1 μM WP1130 for 6 h. Cells were then incubated with 10 μM MG-132 for an additional 6 h. Cellular lysates were subjected to sequential IP and immunoblotting assays with the indicated antibodies.

(**F**) MCF7 and T47D cells were treated with or without 1 μM WP1130 for the indicated times, followed by immunoblotting assays.

(**G**) HEK293T cells were transfected with the indicated expression vectors for 40 h, followed by treatment with or without 1 μM WP1130 for 6 h. Cells were then incubated with 10 μM MG-132 for an additional 6 h. Cellular lysates were subjected to sequential IP and immunoblotting assays with the indicated antibodies.

(**H**) MCF7 and T47D cells stably expressing pLVX or Flag-KIFC2 were treated with or without 1 μM WP1130 for 6 h, followed by immunoblotting assays.

(**I**) HEK293T cells were transfected with indicated plasmids for 24 h, followed by passaging. Cells were then either infected with shNC or sh*KIFC2* #1 viruses for 48 h, and subsequently incubated with MG-132 for additional 6 h. Then, cells were collected for IP and immunoblotting assays.

(**J and K**) Immunoblotting analysis of USP9X protein levels in KIFC2-overexpressing (J) and -depleted (K) MCF7 and T47D cells.

Data are mean ± SD (**A**) (n = 3 per group).

WP1130, USP9X inhibitor.

***, p<0 .001; ns, non-significant.

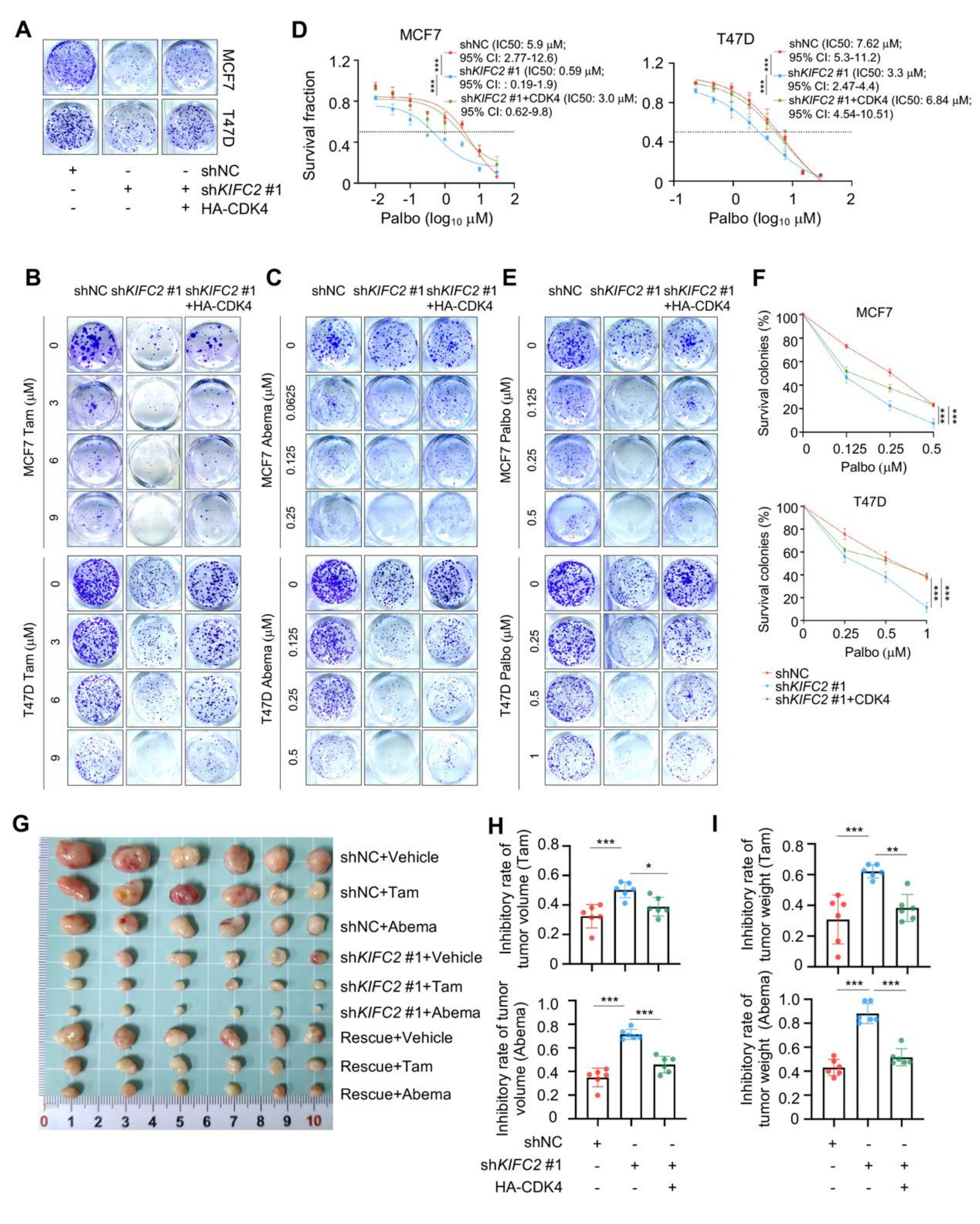

Supplemental Figure 13. Re-expression CDK4 in KIFC2-depleted cells reverses the enhanced sensitivity of HR+/HER2- BC cells to Tamoxifen and CDK4/6 inhibitor caused by KIFC2 knockdown.

(**A**) Cells stably expressing shNC, sh*KIFC2* #1 alone or in combination with HA-CDK4 were subjected to colony formation assays. Images of colonies are shown.

(**B and C**) Cells stably expressing shNC, sh*KIFC2* #1, or in combination with HA-CDK4 were subjected to clonogenic survival assays in the presence or absence of Tam (B) or Abema (C) for 7-9 days. Images of surviving colonies are shown.

(**D**) Cells stably expressing shNC, sh*KIFC2* #1 alone or in combination with HA-CDK4 were treated with or without increasing concentrations of Palbo for 72 h, and then subjected to CCK-8 assays to evaluate the IC50 values.

(**E and F**) Cells stably expressing shNC, sh*KIFC2* #1 alone or in combination with HA-CDK4 were subjected to clonogenic survival assays in the presence or absence of increasing concentrations of Palbo for 7-9 days. The representative images of the survival colonies (E) and the corresponding quantitative analysis (F) are shown.

(**G-I**) MCF7 cells stably expressing shNC, sh*KIFC2* #1 or sh*KIFC2* #1+HA-CDK4 were injected into mammary fat pad of BALB/c female nude mice. After 18 days of injection, mice were administered with vehicle, Tam or Abema. The xenograft tumors (G), inhibitory rate of tumor volume (H), and inhibitory rate of tumor weight (I) are shown. Rescue refers to KIFC2-depleted cells with re-expression of CDK4.

Data are mean ± SD (**D** and **F**) (n = 3 per group); (**H** and **I**) (n = 6 per group).

Statistical analysis: (**D**) extra-sum-of-squares F test; (**F, H, and I**) one-way ANOVA.

Tam, tamoxifen; Abema, abemaciclib; Palbo, palbociclib.

*, p<0 .05; **, p<0 .01; ***, p<0 .001.

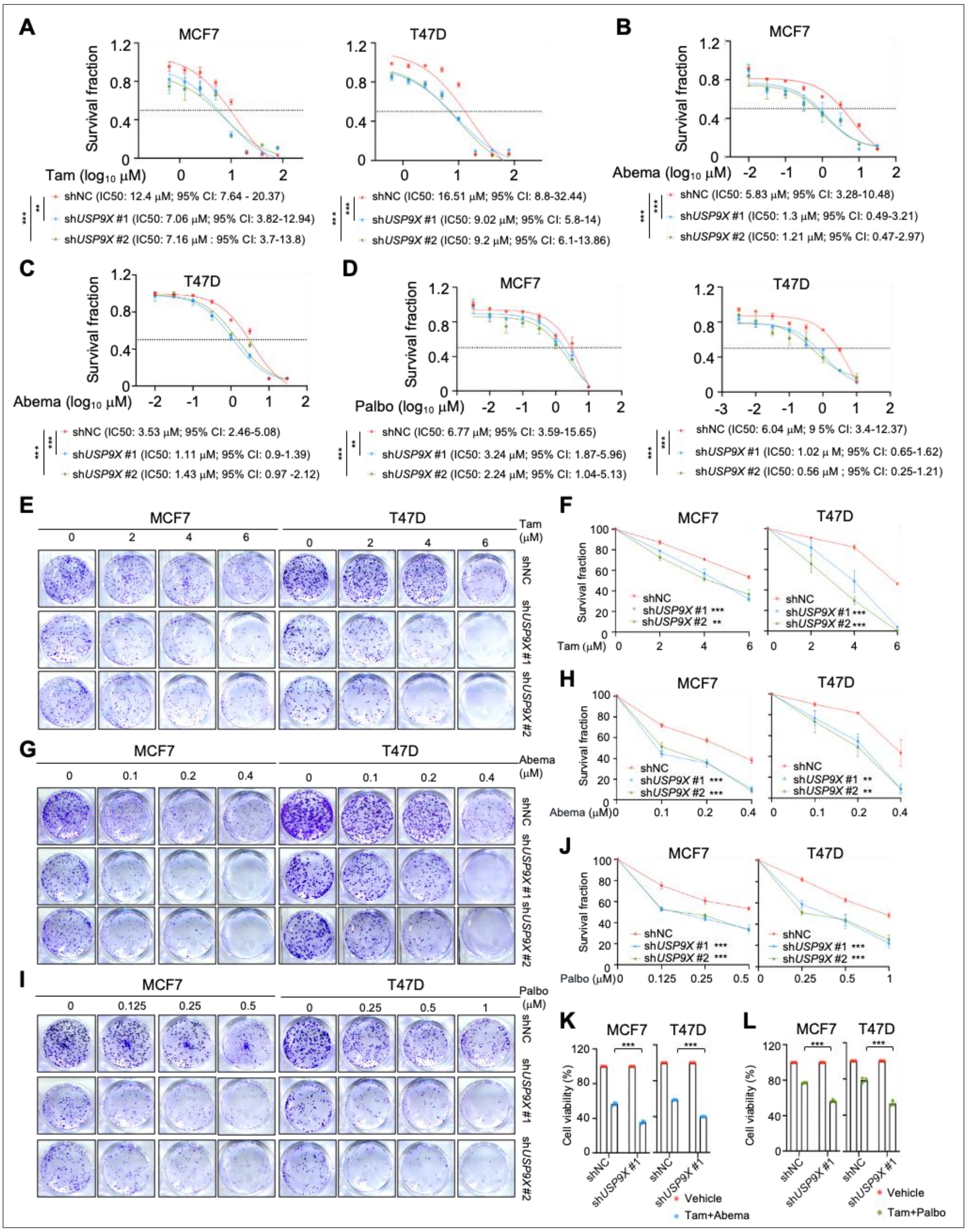

Supplemental Figure 14. Knockdown of USP9X enhances the sensitivity of HR+/HER2- BC cells to Tam and CDK4/6 inhibitors.

(**A-D**) MCF7 and T47D cells stably expressing shNC or sh*USP9X* (#1 and #2) were treated with or without increasing concentrations of Tam (A), Abema (B and C), and Palbo (D) for 72 h and subjected to CCK-8 assays to evaluate IC50 values.

(E-J) MCF7 and T47D cells stably expressing shNC or sh*USP9X* (#1 and #2) were subjected to clonogenic survival assays in the presence or absence of increasing concentrations of Tam

(E and F), Abema (G and H), or Palbo (I and J) for 7-9 days. The representative images of the survival colonies are shown in E, G, and I. The corresponding quantitative results are shown F, H, and J.

(**K and L**) MCF7 and T47D cells stably expressing shNC or sh*USP9X* #1were treated with or without Tam plus Abema (K) or Tam plus Palbo (L) for 72 h and then subjected to CCK-8 assays.

Data are mean ± SD (**A-D, F, H, J, K,** and **L**) (n = 3 per group).

Statistical analysis: (**A**-**D**) extra-sum-of-squares F test; (**F, H,** and **J**) one-way ANOVA; (**K** and **L**) two-tailed Student’s *t* test.

Tam, tamoxifen; Abema, abemaciclib; Palbo, palbociclib.

, *p*<0 .01; *, *p*<0 .001.

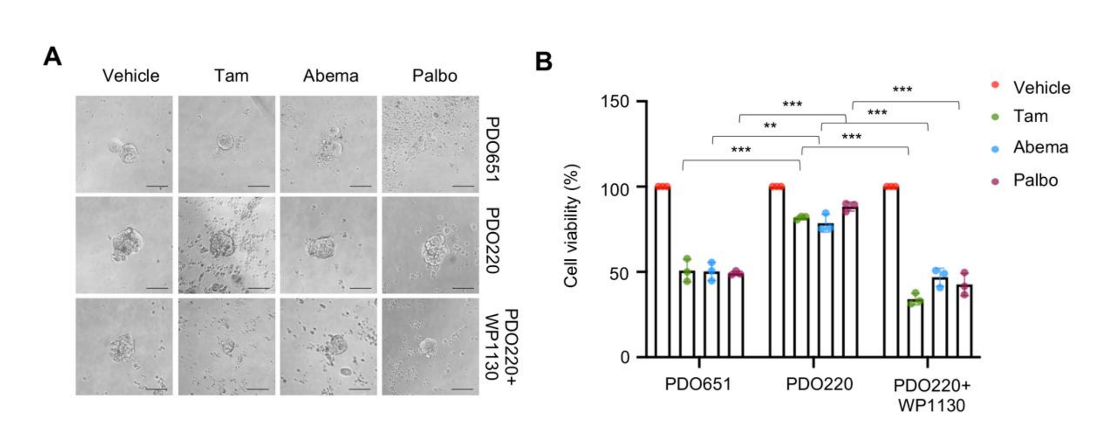

Supplemental Figure 15. WP1130 enhances the sensitivity of resistant PDO220 to Tam, Abema, and Palbo.

(**A-B**) CellTiter-Glo 3D cell viability assays were performed in two HR+/HER2- BC PDOs treated without or with WP1130, and further treated with Tam (2 μM), Abema (0.5 μM), and Palbo (1 μM). Representative images of the organoids after drug treatment (A) and the corresponding quantitative results (B) are shown. One-way ANOVA test. Scale bar: 100 μm.

Data are mean ± SD (**B**) (n = 3 per group).

Tam, tamoxifen; Abema, abemaciclib; Palbo, palbociclib; PDO, patient-derived organoid; WP1130, USP9X inhibitor.

**, $p<0.01$; ***, $p<0.001$.

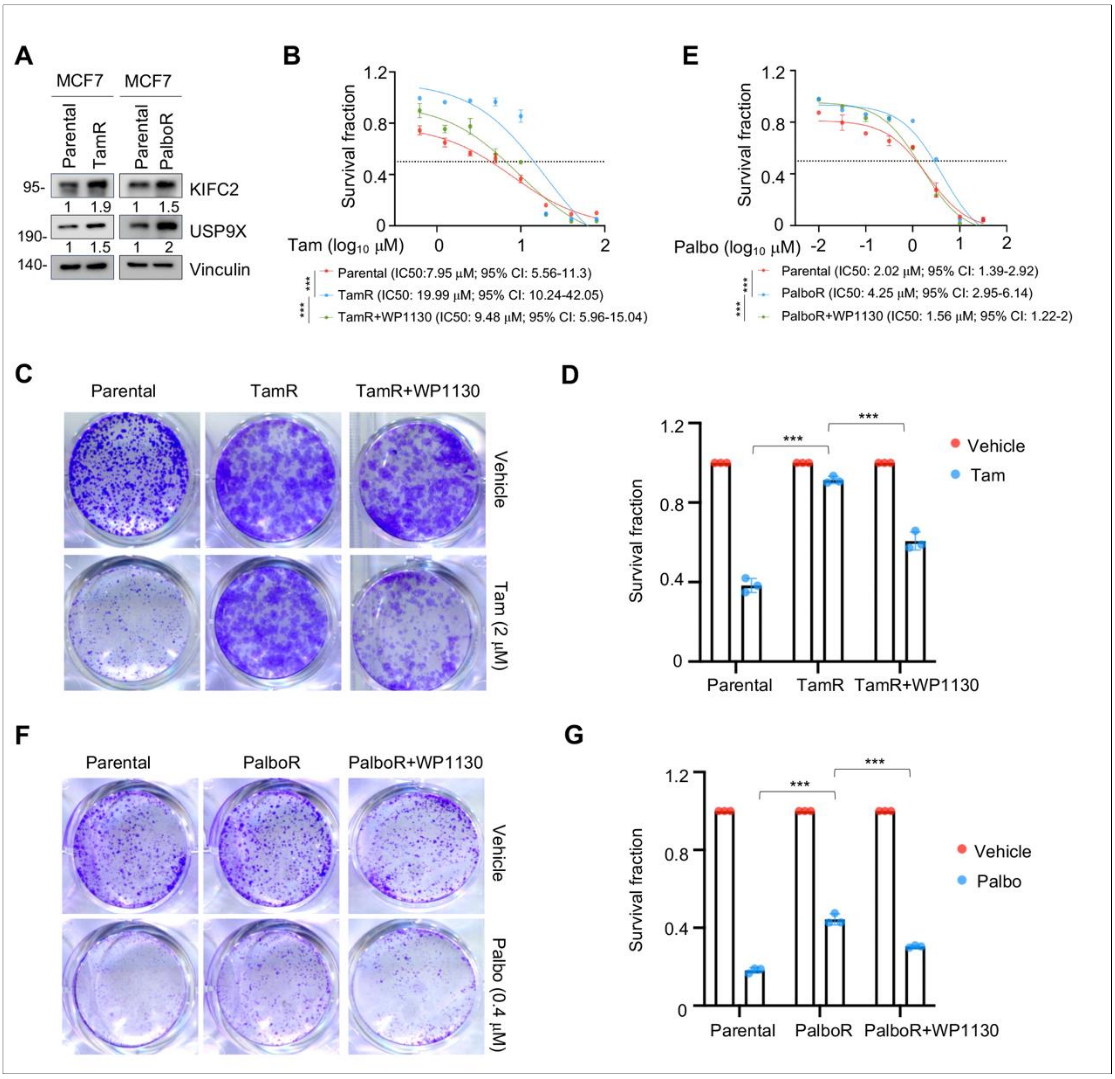

Supplemental Figure 16. WP1130 enhances cellular sensitivity of tamoxifen-resistant (TamR) and palbociclib-resistant (PalboR) MCF7 cell lines to Tam and Palbo, respectively.

(**A**) Immunoblotting analysis of KIFC2 and USP9X protein levels in parental, MCF7-TamR, and MCF7-PalboR cell lines.

(**B**) Parental MCF7 and MCF7-TamR cells were treated with or without 1 μM WP1130 for 24 h, followed by treatment with increasing concentrations of Tam for 72 h, and then subjected to CCK-8 assays to evaluate the IC50 values.

(**C-D**) Parental MCF7 and MCF7-TamR cells were treated with or without 1 μM WP1130 for 24 h, and then subjected to clonogenic survival assays in Tam for 7-9 days. The representative images of the survival colonies (C) and the corresponding quantitative analysis (D) are shown.

(**E**) Parental MCF7 and MCF7-PalboR cells were treated with or without 1 μM WP1130 for 24 h, followed by treatment with increasing concentrations of Palbo for 72 h, and then subjected to CCK-8 assays to evaluate the IC50 values.

(**F-G**) Parental MCF7 and MCF7-PalboR cells were treated with or without 1 μM WP1130 for 24 h, and then subjected to clonogenic survival assays in Palbo for 7-9 days. The representative images of the survival colonies (F) and the corresponding quantitative analysis (G) are shown.

Data are mean ± SD (**B, D, E,** and **G**) (n = 3 per group).

Statistical analysis: (**B** and **E**) extra-sum-of-squares F test; (**D** and **G**) one-way ANOVA.

Tam, tamoxifen; Palbo, palbociclib; WP1130, USP9X inhibitor.

***, p<0 .001.

Supplemental Tables

Supplemental Table 1. The members of the KIF superfamily

Genes	Kinesin Families
KIF5A	Kinesin-1
KIF5B	Kinesin-1
KIF5C	Kinesin-1
KIF3A	Kinesin-2
KIF3B	Kinesin-2
KIF3C	Kinesin-2
KIF17	Kinesin-2
KIF1A	Kinesin-3
KIF1B	Kinesin-3
KIF1C	Kinesin-3
KIF13A	Kinesin-3
KIF13B	Kinesin-3
KIF14	Kinesin-3
KIF16A	Kinesin-3
KIF16B	Kinesin-3
KIF4A	Kinesin-4
KIF4B	Kinesin-4
KIF7	Kinesin-4
KIF21A	Kinesin-4
KIF21B	Kinesin-4
KIF27	Kinesin-4
KIF11	Kinesin-5
KIF20A	Kinesin-6
KIF20B	Kinesin-6
KIF23	Kinesin-6
KIF10	Kinesin-7
KIF18A	Kinesin-8
KIF18B	Kinesin-8
KIF19A	Kinesin-8
KIF19B	Kinesin-8
KIF6	Kinesin-9
KIF9	Kinesin-9
KIF22	Kinesin-10
KIF26A	Kinesin-11
KIF26B	Kinesin-11
KIF12	Kinesin-12
KIF15	Kinesin-12
KIF2A	Kinesin-13
KIF2B	Kinesin-13

KIF2C	Kinesin-13
KIF24	Kinesin-13
KIF25	Kinesin-14
KIFC1	Kinesin-14
KIFC2	Kinesin-14
KIFC3	Kinesin-14

Supplemental Table 2. Chemicals used in this study

Chemicals	Vendors	Catalog number
Cycloheximide	Cell signaling technology	2112S
Capecitabine	MedChemExpress	HY-B0016
WP1130	MedChemExpress	HY-13264
Tamoxifen (in vivo)	Selleck	S1238
Abemaciclib	Selleck	S5716
Palbociclib	Selleck	S1579
MG-132	Selleck	S2619
Bafilomycin A1	Selleck	S1413
4-hydroxytamoxifen (in vitro)	Sigma	H6278

Supplemental Table 3. Primers used for molecular cloning

Plasmids	Primers	Sequences
pLVX-Flag-KIFC2	Forward	GTCGTCGTCGTCGTCGAATTCGCCACCATGTACGCCTTTTACTCGTTGCTCATC
	Reverse	GGAGGGAGAGGGGCGGGATCCTCACCGCCTCTGCCAGCAG
pLVX-Flag-CDK4	Forward	GGATCTATTTCCGGTGAATTCGCCACCATGGCTACCTCTCGATATGAG
	Reverse	GGGATCCGCGGCCGCTCTAGATTACTTGTCATCGTCGTCCTTGTAATCCTCCGGATTACCTTCATCCTT
pLVX-HA-CDK4	Forward	GGATCTATTTCCGGTGAATTCGCCACCATGGCTACCTCTCGATATGAG
	Reverse	GGGATCCGCGGCCGCTCTAGATTAAGCGTAGTCTGGGACGTCGTATGGGTACTCCGGATTACCTTCATCCTT
pLVX-HA-p53	Forward	GGATCTATTTCCGGTGAATTCGCCACCATGGAGGAGCCGCAGTCAGAT
	Reverse	GGGATCCGCGGCCGCTCTAGATTAAGCGTAGTCTGGGACGTCGTATGGGTAGTCTGAGTCAGGCCCTTCTGTCTT
pLVX-HA-p53-R175H	Forward	GAGGTTGTGAGGCACTGCCCCCACCATG
	Reverse	CATGGTGGGGGCAGTGCCTCACAACCTC
pLVX-HA-p53-Y220C	Forward	GTGTGGTGGTGCCCTGCGAGCCGCCTGAGGTTG
	Reverse	CAACCTCAGGCGGCTCGCAGGGCACCACCACAC
pLVX-HA-p53-R248W	Forward	GGGCGGCATGAACTGGAGGCCCATCC
	Reverse	GGATGGGCCTCCAGTTCATGCCGCCC
pLVX-HA-p53-R273H	Forward	GAACAGCTTTGAGGTGCACGTTTGTGCCTGTCCTGG
	Reverse	CCAGGACAGGCACAAACGTGCACCTCAAAGCTGTTC

Supplemental Table 4. shRNA targeting sequences

**shRNAs**	**Primers**	**Sequences**
sh*KIFC2* #1	Forward	CCGGGCTCATCTACATCTTCTACAGCTCGAGCTGTAGAAGATGTAGATGAGCTTTTTG
	Reverse	AATTCAAAAAGCTCATCTACATCTTCTACAGCTCGAGCTGTAGAAGATGTAGATGAGC
sh*KIFC2* #2	Forward	CCGGGGATCCACATCCCAAGAAGAACTCGAGTTCTTCTTGGGATGTGGATCCTTTTTG
	Reverse	AATTCAAAAAGGATCCACATCCCAAGAAGAACTCGAGTTCTTCTTGGGATGTGGATCC
sh*CDK4* #1	Forward	CCGGGCATGTAGACCAGGACCTAAGCTCGAGCTTAGGTCCTGGTCTACATGCTTTTTG
	Reverse	AATTCAAAAAGCATGTAGACCAGGACCTAAGCTCGAGCTTAGGTCCTGGTCTACATGC
sh*CDK4* #2	Forward	CCGGGGATCTGATGCGCCAGTTTCTCTCGAGAGAAACTGGCGCATCAGATCCTTTTTG
	Reverse	AATTCAAAAAGGATCTGATGCGCCAGTTTCTCTCGAGAGAAACTGGCGCATCAGATCC
sh*USP9X* #1	Forward	CCGGGAGAGTTTATTCACTGTCTTACTCGAGTAAGACAGTGAATAAACTCTCTTTTTG
	Reverse	AATTCAAAAAGAGAGTTTATTCACTGTCTTACTCGAGTAAGACAGTGAATAAACTCTC
sh*USP9X* #2	Forward	CCGGCGATTCTTCAAAGCTGTGAATCTCGAGATTCACAGCTTTGAAGAATCGTTTTTG
	Reverse	AATTCAAAAACGATTCTTCAAAGCTGTGAATCTCGAGATTCACAGCTTTGAAGAATCG

Supplemental Table 5. Antibodies used in this study

Antibodies	Vendors	Catalog number
RB	Abcam	ab181616
Ki-67	Abcam	ab15580
LC3A/B	Cell signaling technology	12741
V5	Cell signaling technology	13202S
USP9X	Cell signaling technology	14898S
Phospho-Rb (Ser807/811)	Cell signaling technology	8516T
HA	Cell signaling technology	3724S
p21	Cell signaling technology	2947S
p53	Cell signaling technology	9282
Rabbit IgG	Cell signaling technology	7074V
Anti-rabbit IgG (H+L), F(ab)2, Fragment (Alexa fluor 555 conjugate)	Cell signaling technology	4413S
Anti-mouse IgG (H+L), F(ab)2, Fragment (Alexa fluor 488 conjugate)	Cell signaling technology	4408S
Anti-mouse IgG (H+L), F(ab)2, Fragment (Alexa fluor 555 conjugate)	Cell signaling technology	4409S
Anti-rabbit IgG (H+L), F(ab)2, Fragment (Alexa fluor 488 conjugate)	Cell signaling technology	4412S
CDK4	SinoBiological	100873-T46
E2F5	SinoBiological	103032-T36
CCNA2	SinoBiological	201099-T46
Flag	Sigma-Aldrich	GNI4110-FG
Vinculin	Sigma-Aldrich	V9131-0.2ML
KIFC2	Thermofisher	PA5-32165

Supplemental Table 6. Primers for RT-qPCR analysis

Genes	Primers	Sequences
KIFC2	Forward	AAGGGAAATATCCGTGTGCTG
	Reverse	GTCTAGGCGGAATCGACGATG
CDK4	Forward	ATGGCTACCTCTCGATATGAGC
	Reverse	CATTGGGGACTCTCACACTCT
USP9X	Forward	TCGGAGGGAATGACAACCAG
	Reverse	GGAGTTGCCGGGGAATTTTCA
GAPDH	Forward	GGAGCGAGATCCCTCCAAAAT
	Reverse	GGCTGTTGTCATACTTCTCATGG
