# Supplementary material for: Kinesin-like protein KIFC2 stabilizes CDK4 to accelerate growth and confer resistance in HR+/HER2– breast cancer
Source: J Clin Invest. 2025 Apr 29;135(12):e183531. doi: 10.1172/JCI183531 (PMC12165803; doi:10.1172/JCI183531)

Figure2A

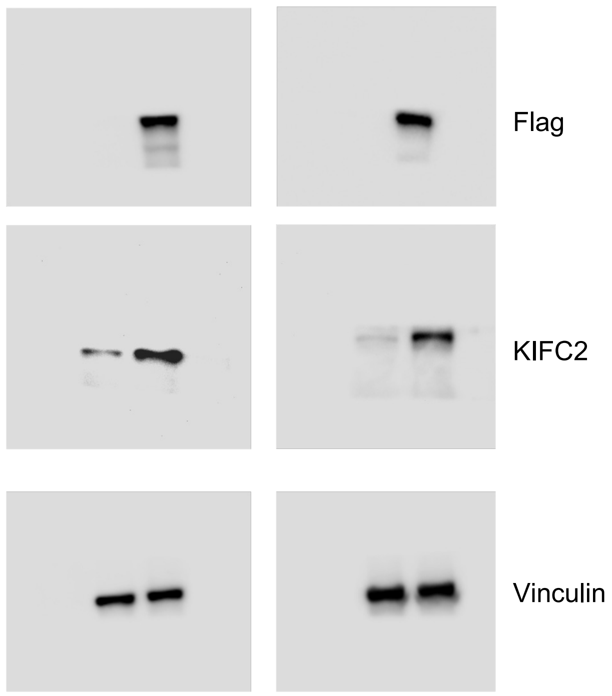

Figure2B

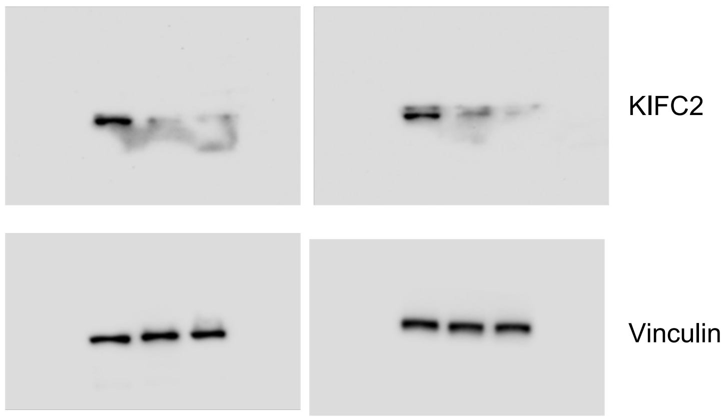

Figure4A

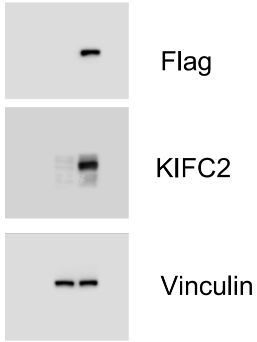

Figure4D

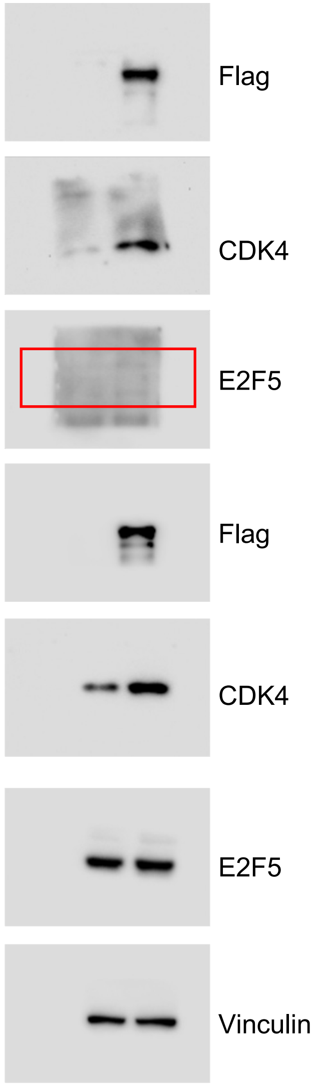

Figure4E

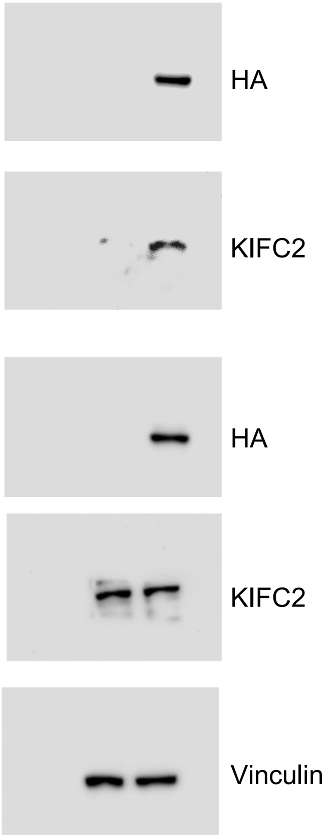

Figure4F

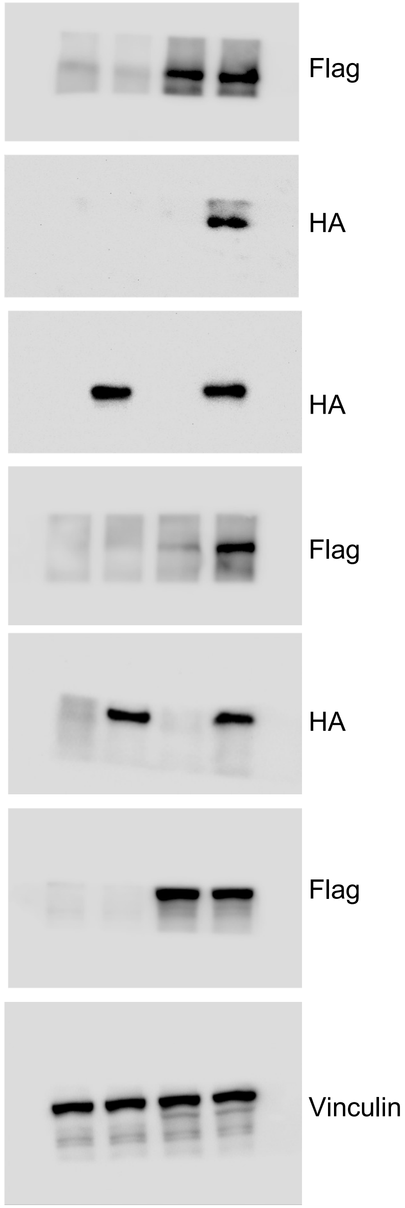

Figure4G

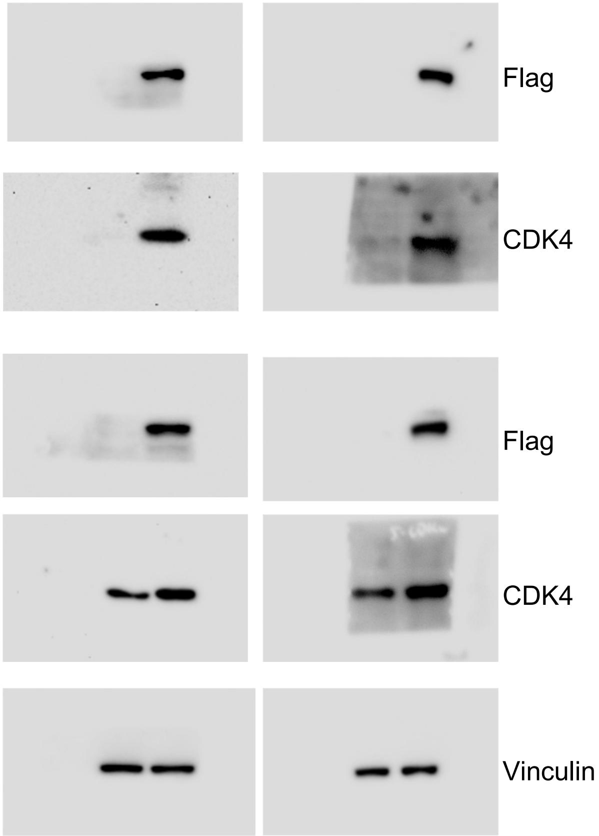

Figure4I

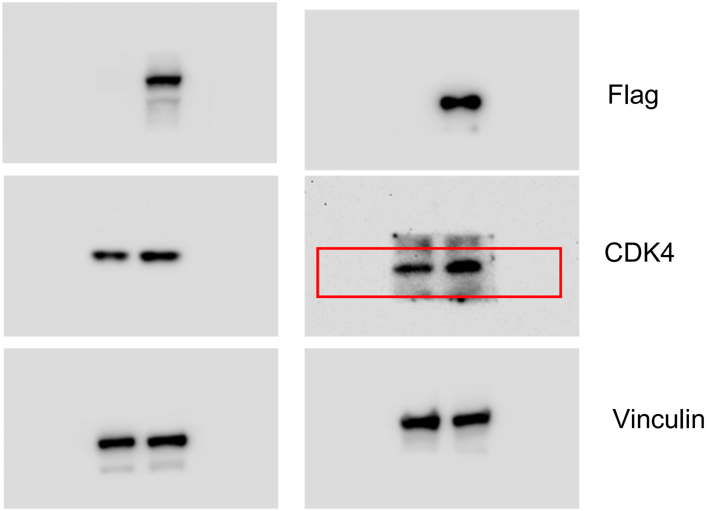

Figure4J

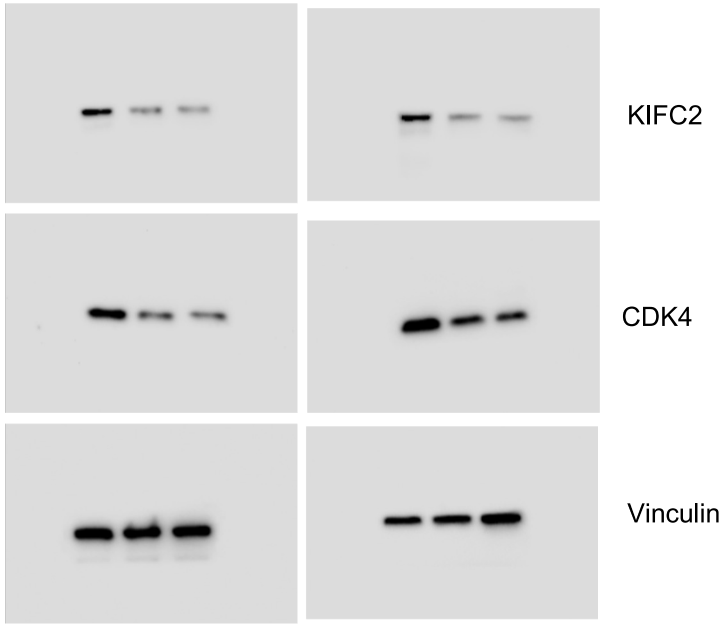

Figure4L

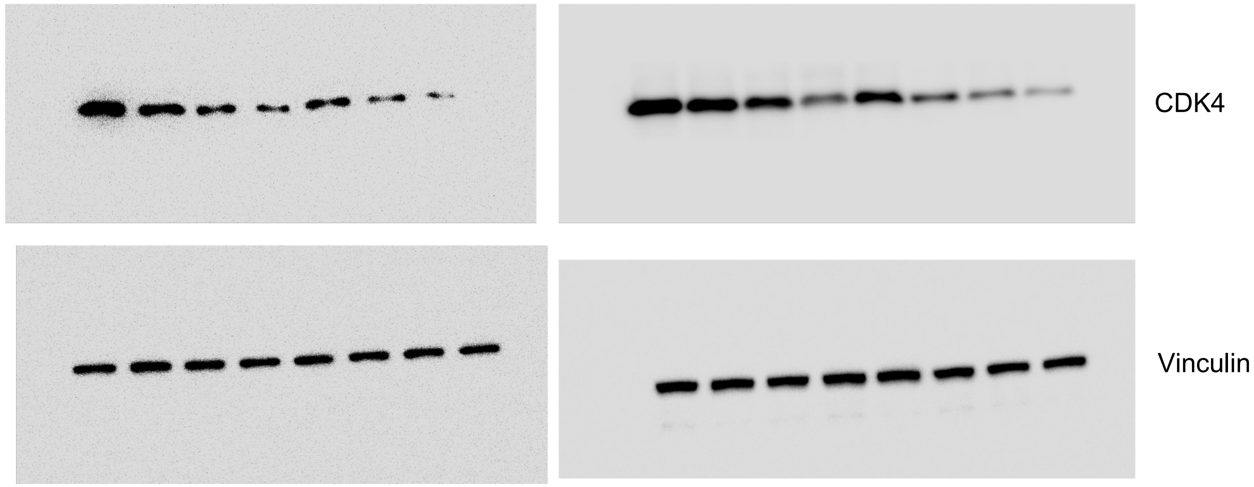

Figure 5A

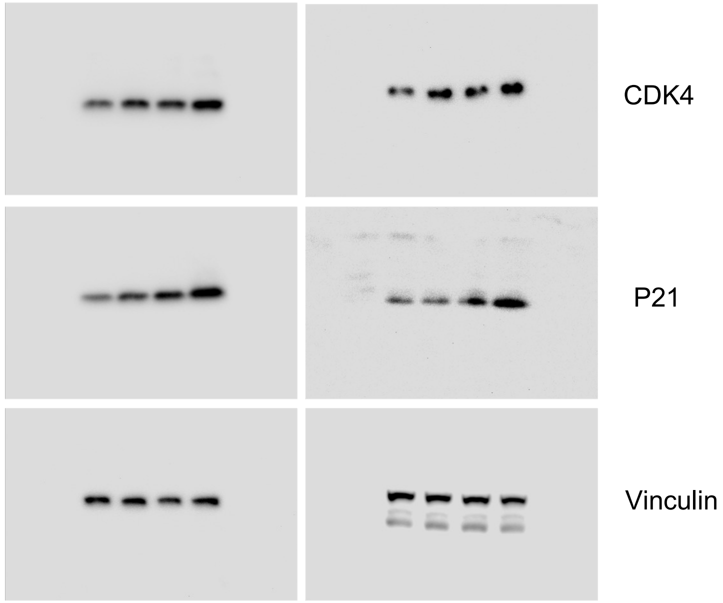

Figure 5B

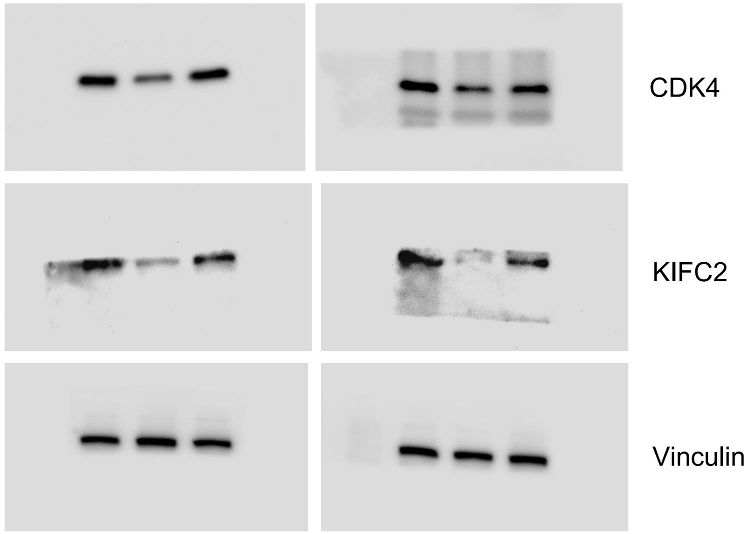

Figure 5C

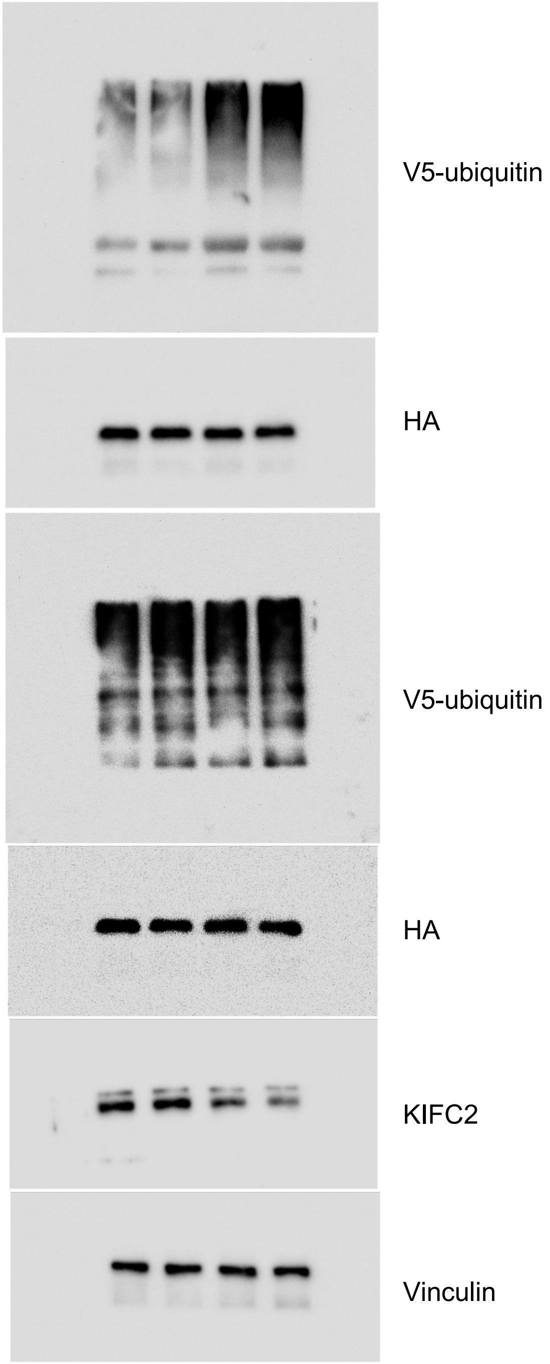

Figure 5D

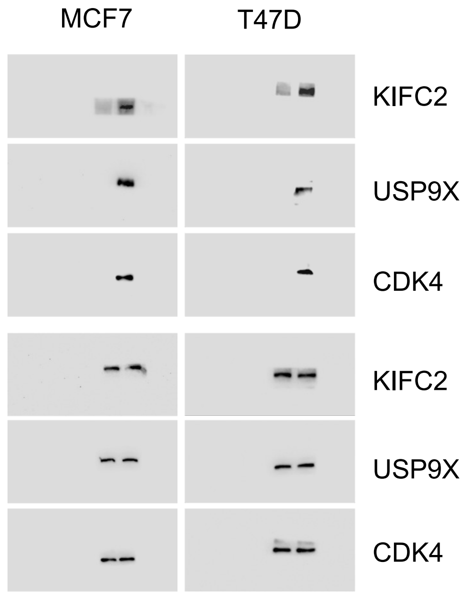

Figure 5E

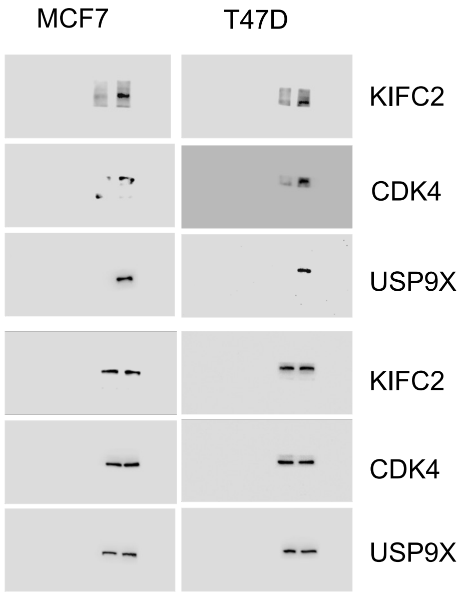

Figure 5F

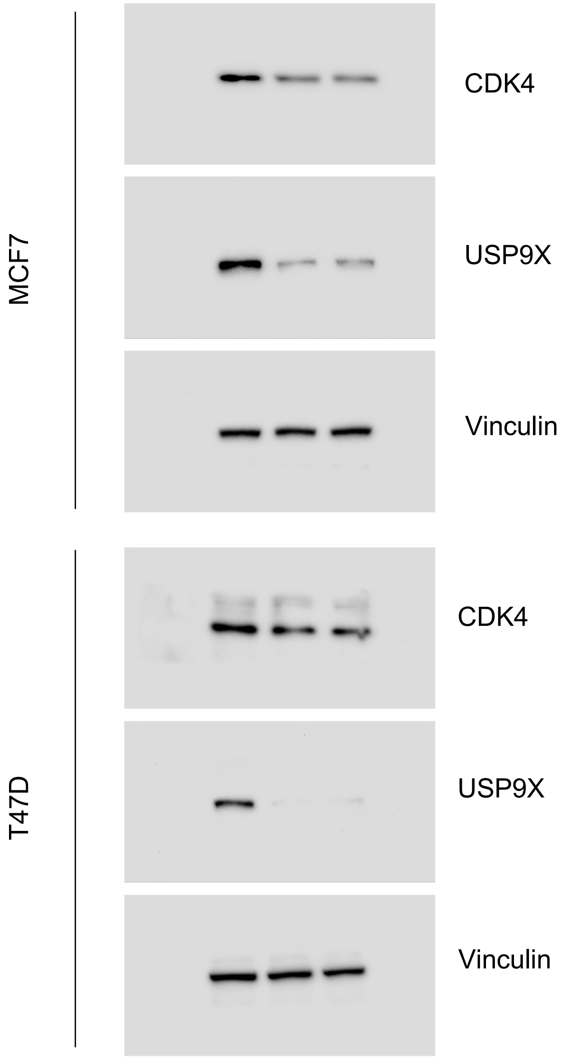

Figure 5G

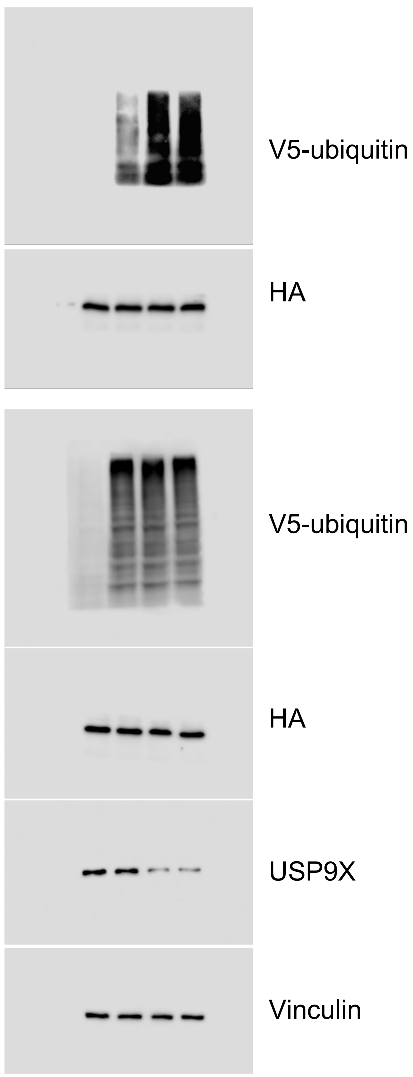

Figure 5H

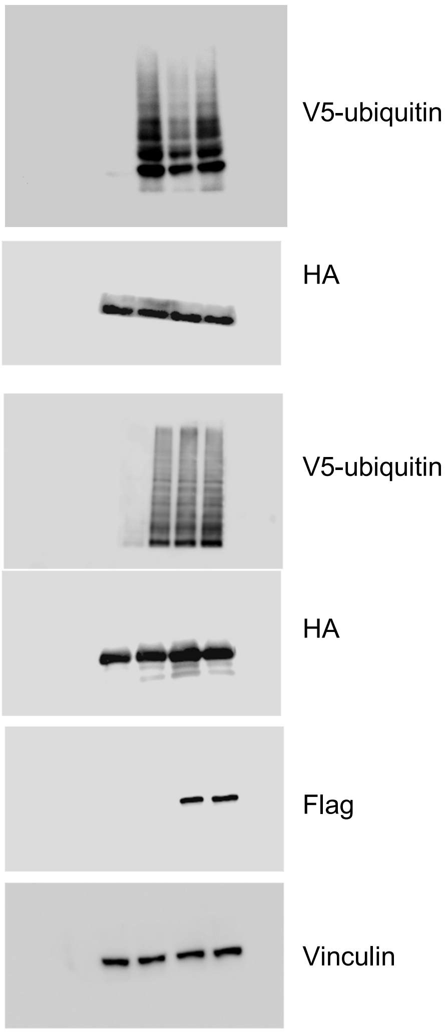

Figure 5I

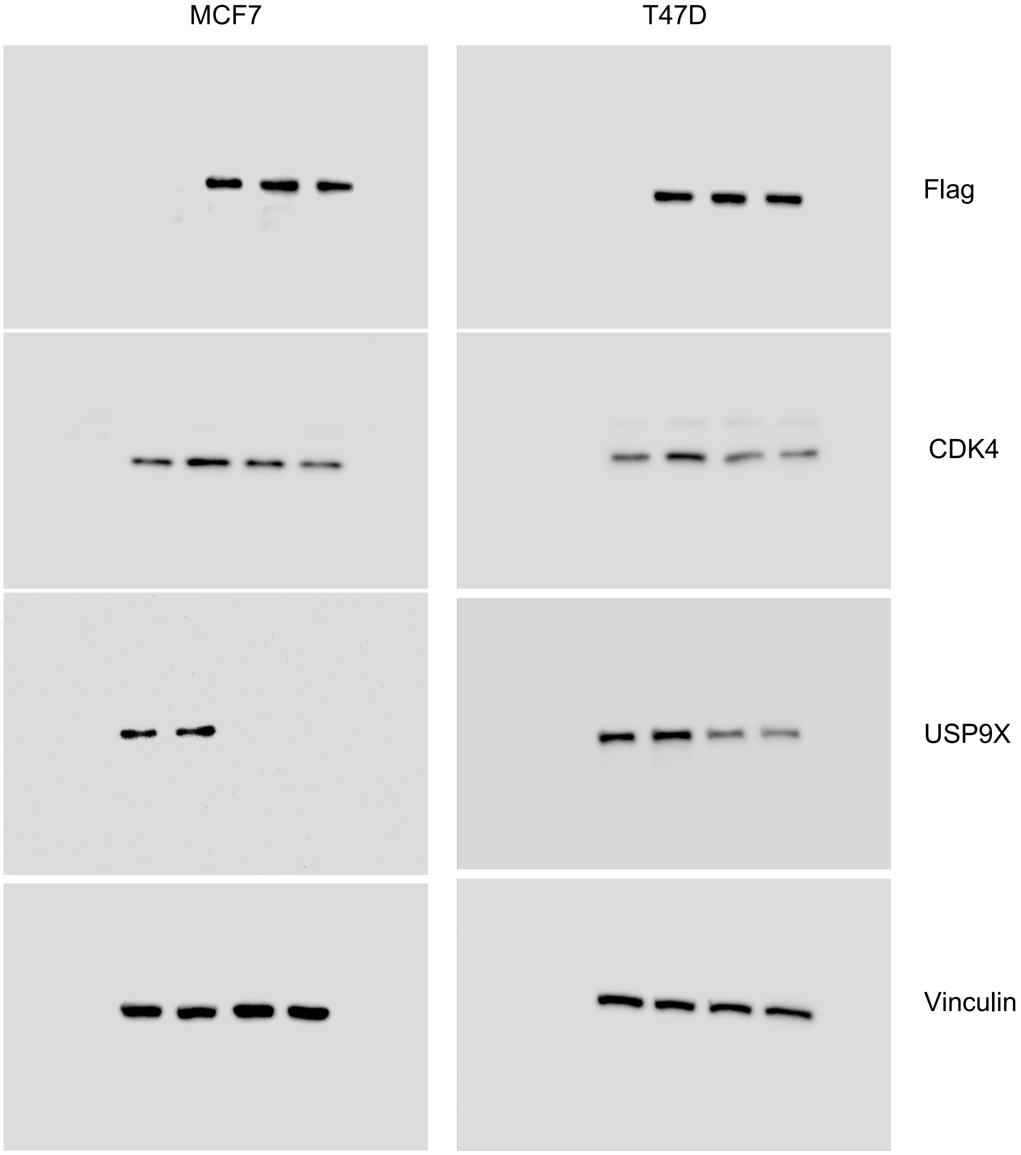

Figure 5J

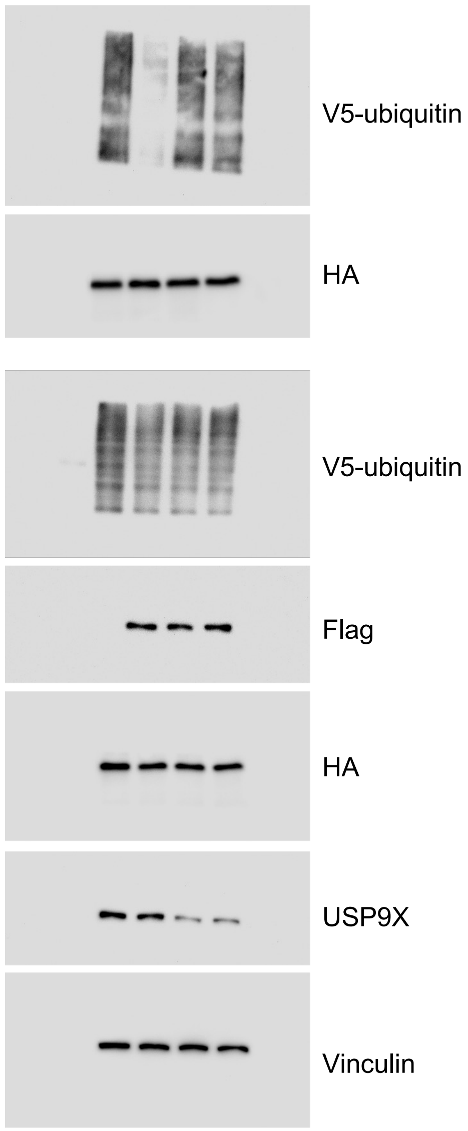

Figure 5K

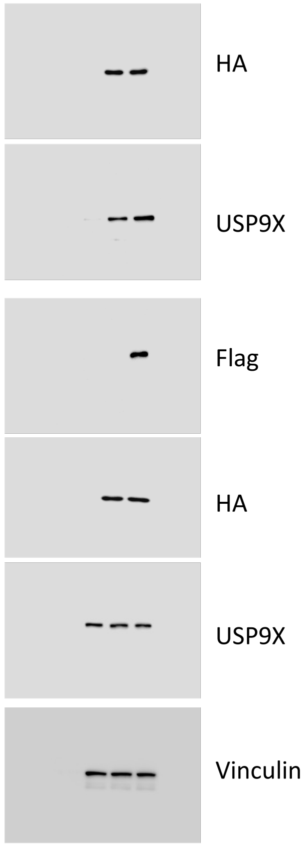

Figure 5L

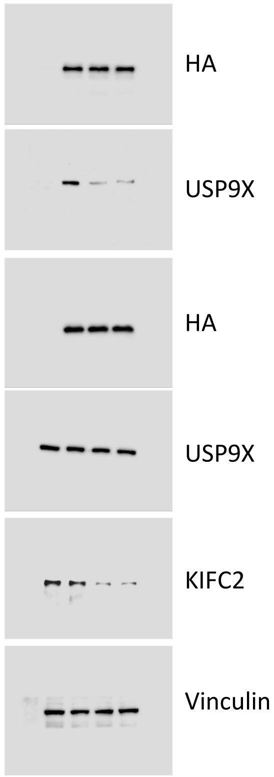

Figure 6A

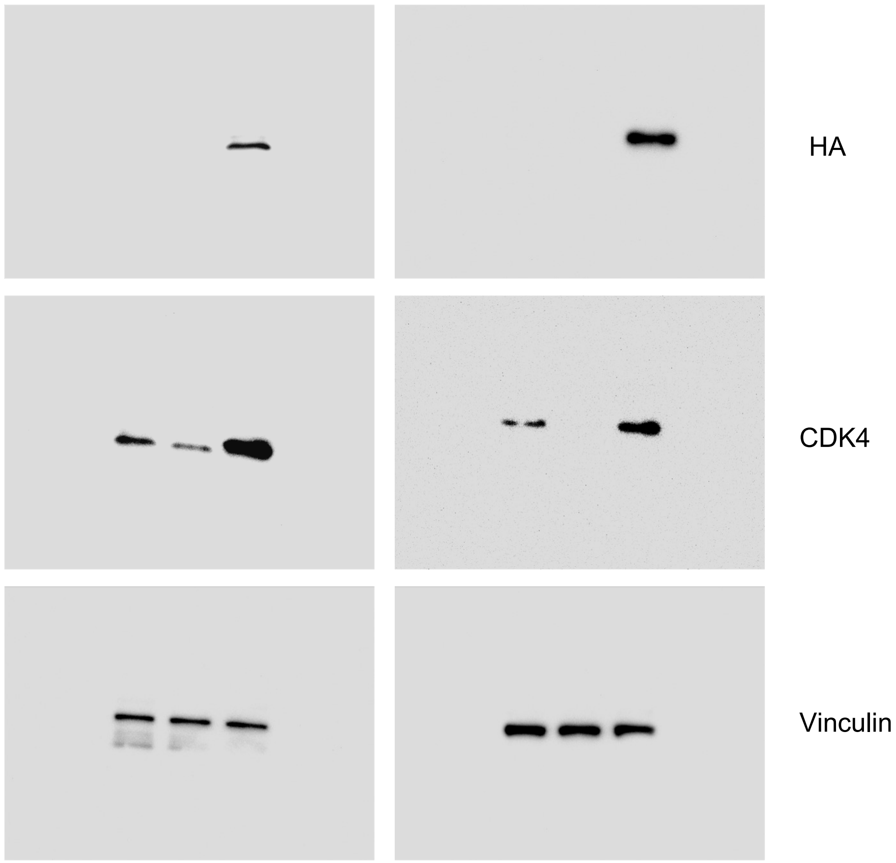

Figure7A

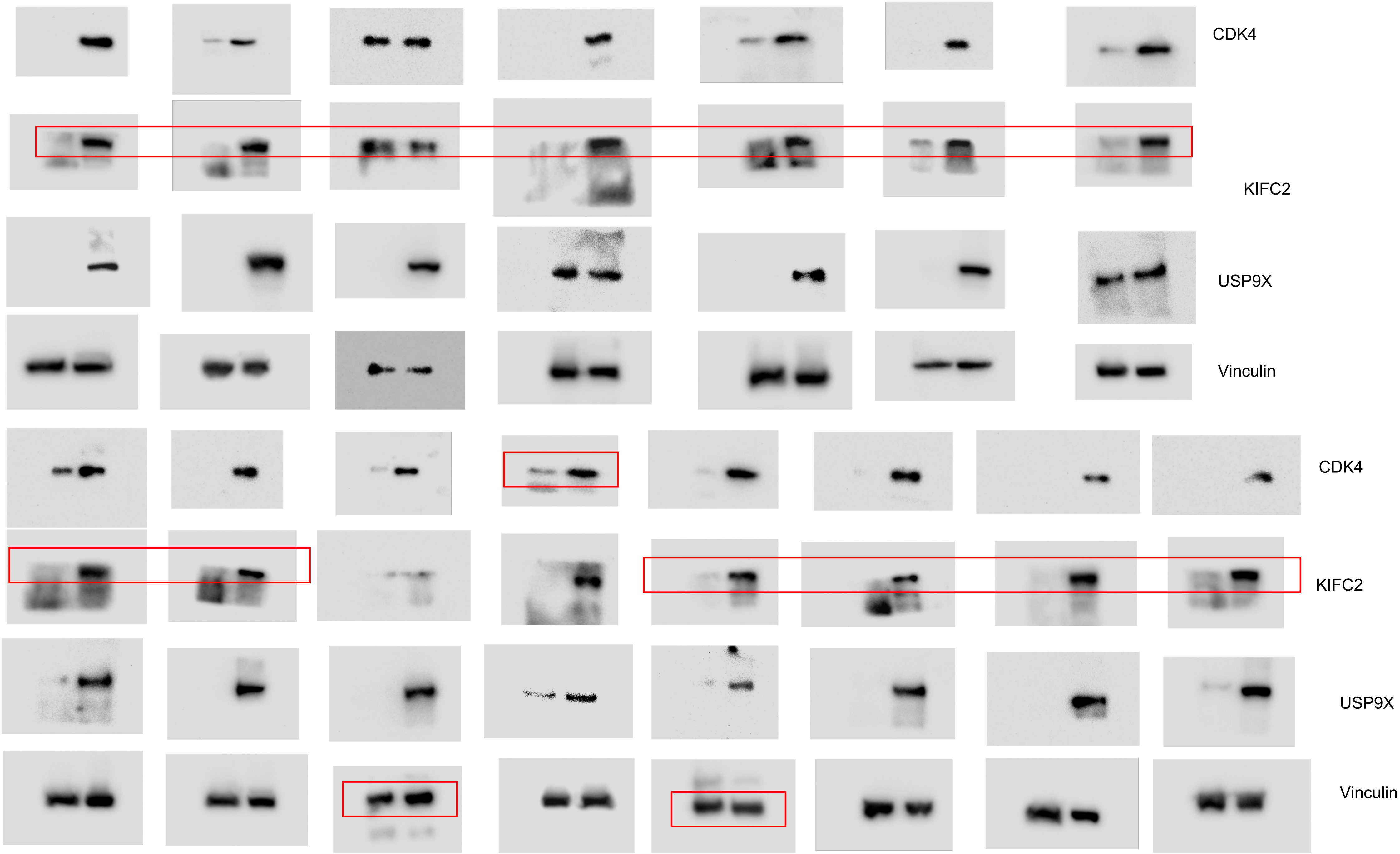

Supplemental Figure1D

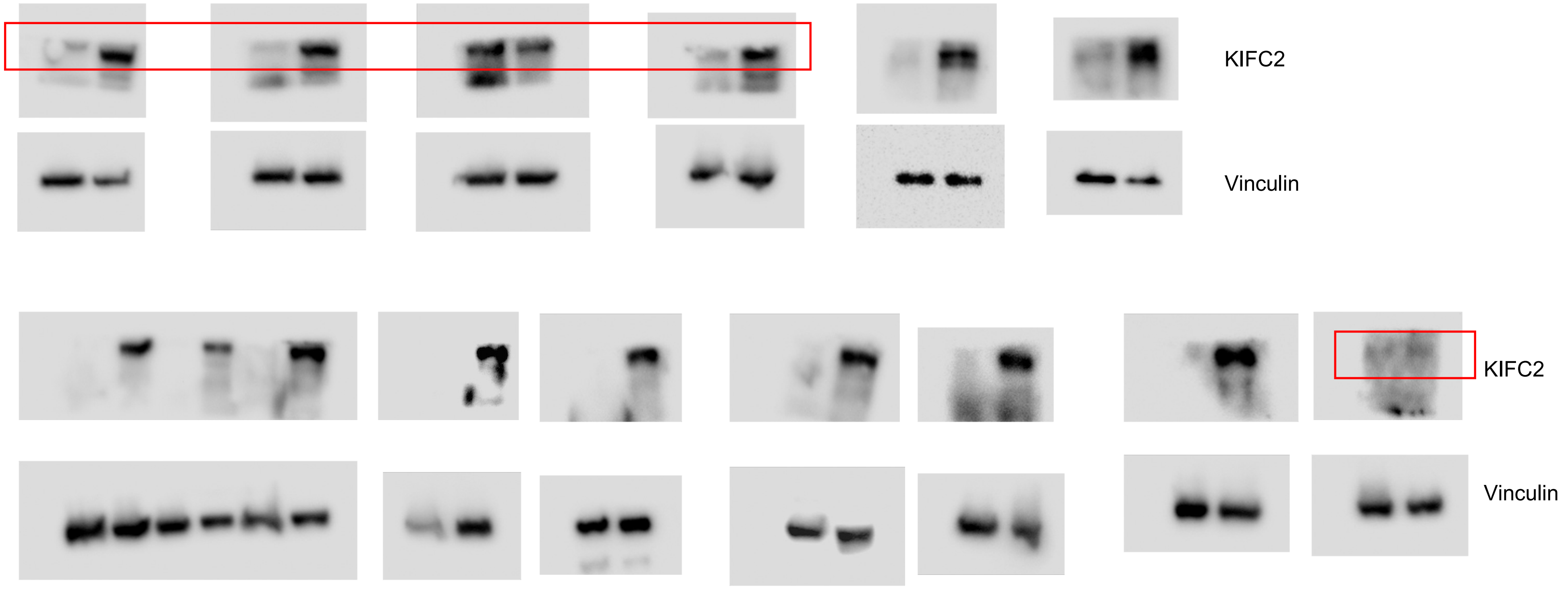

Supplemental Figure 6A

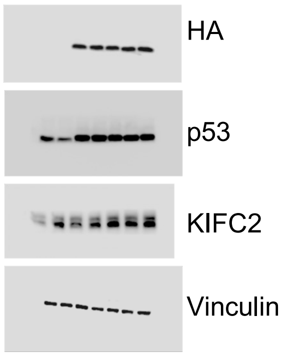

Supplemental Figure 6C

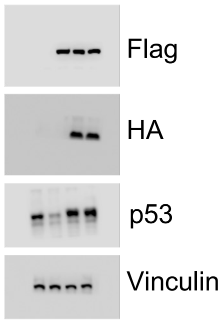

Supplemental Figure 9D

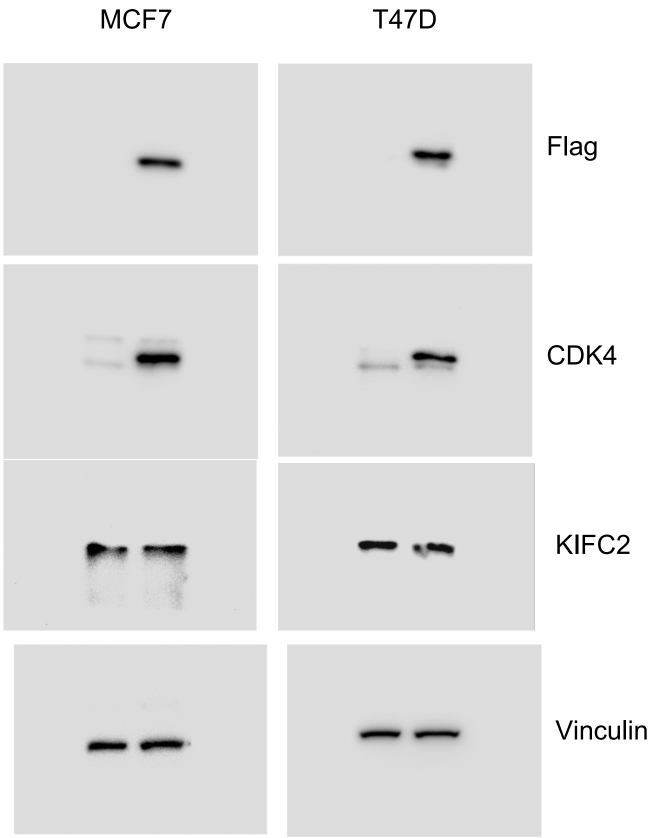

Supplemental Figure 9E

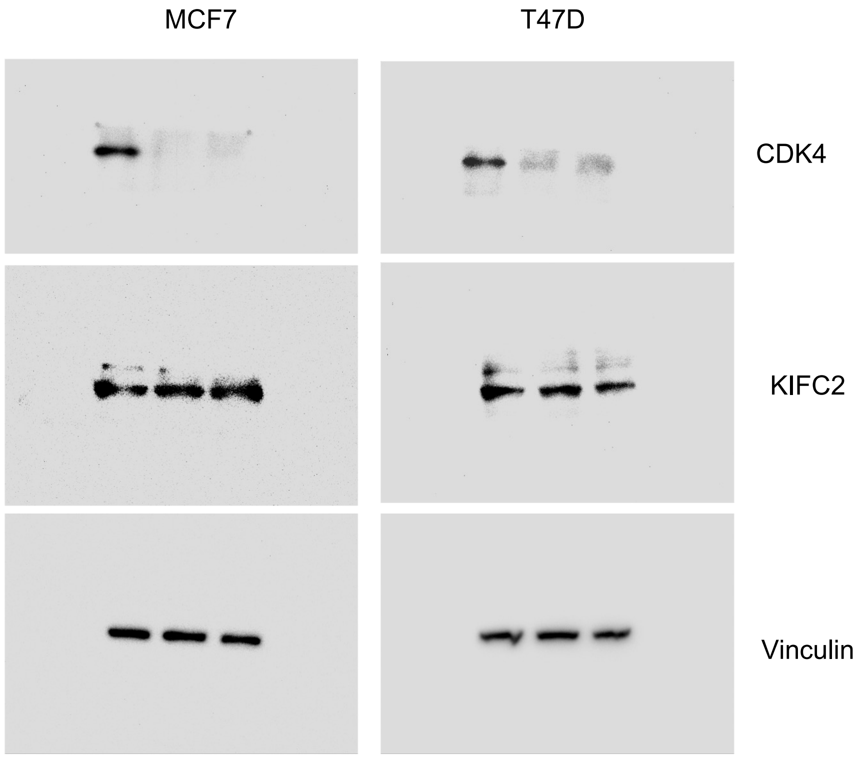

Supplemental Figure 10A

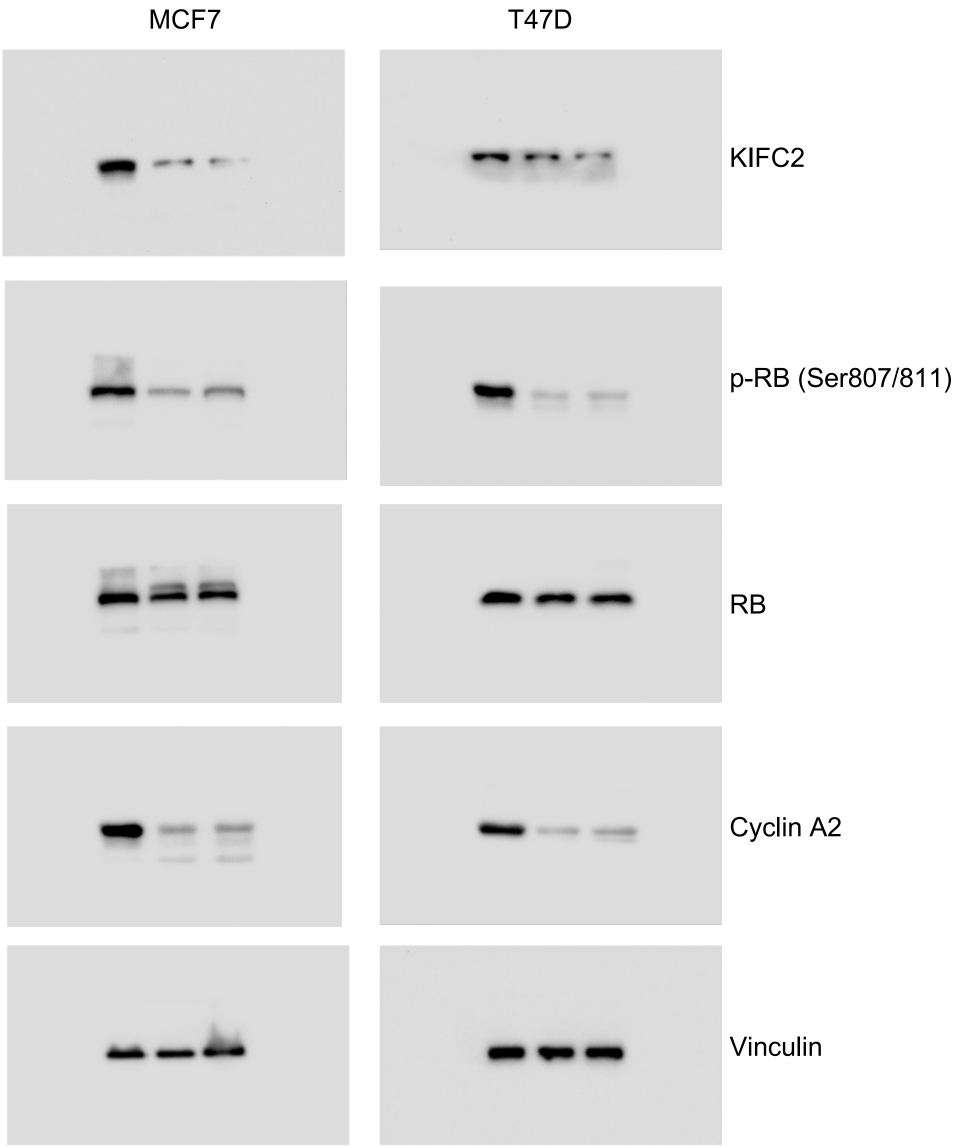

Supplemental Figure 11A

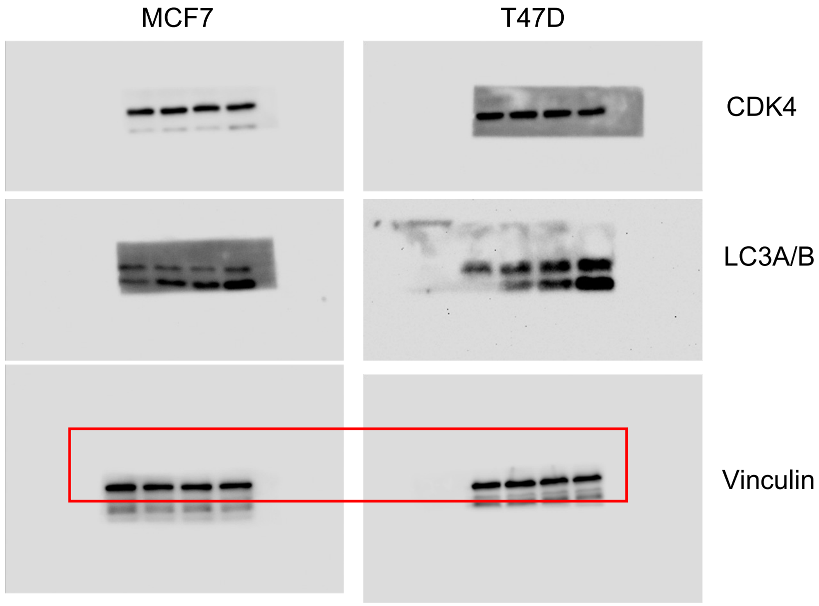

Supplemental Figure 11B

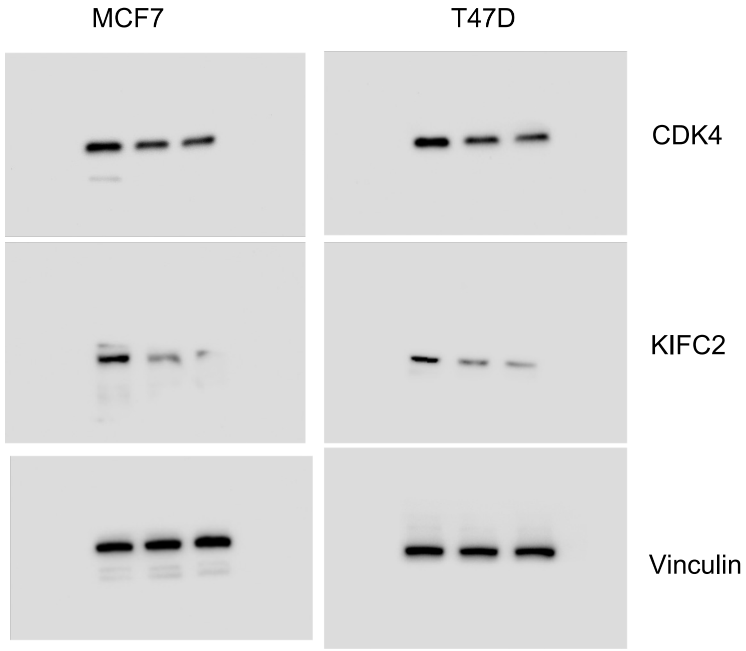

Supplemental Figure 11C

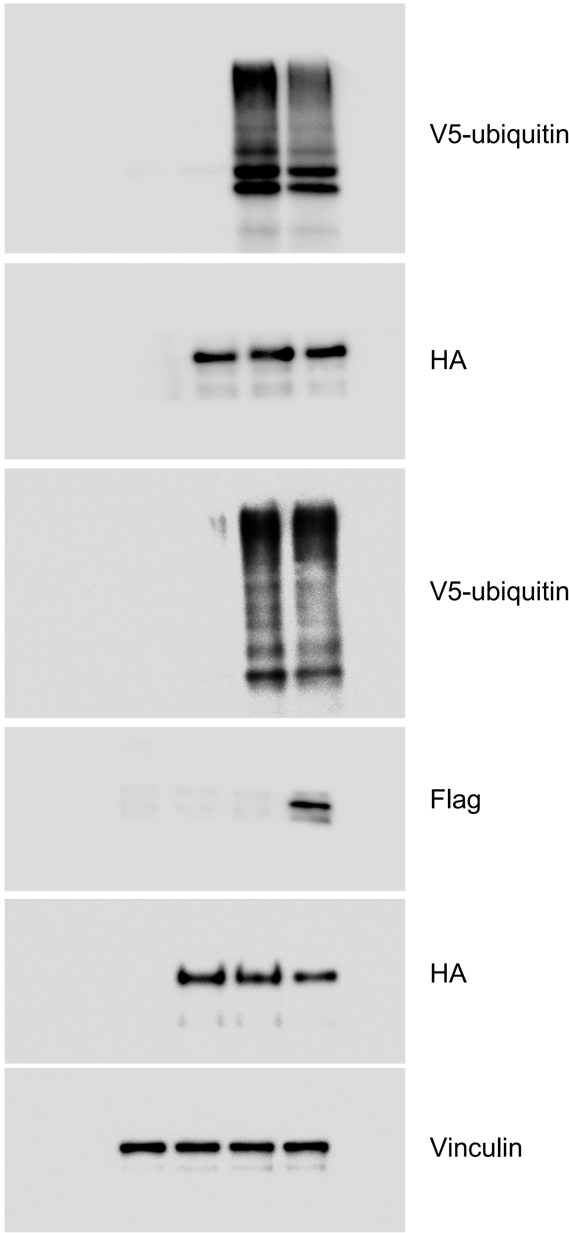

Supplemental Figure 11G and H

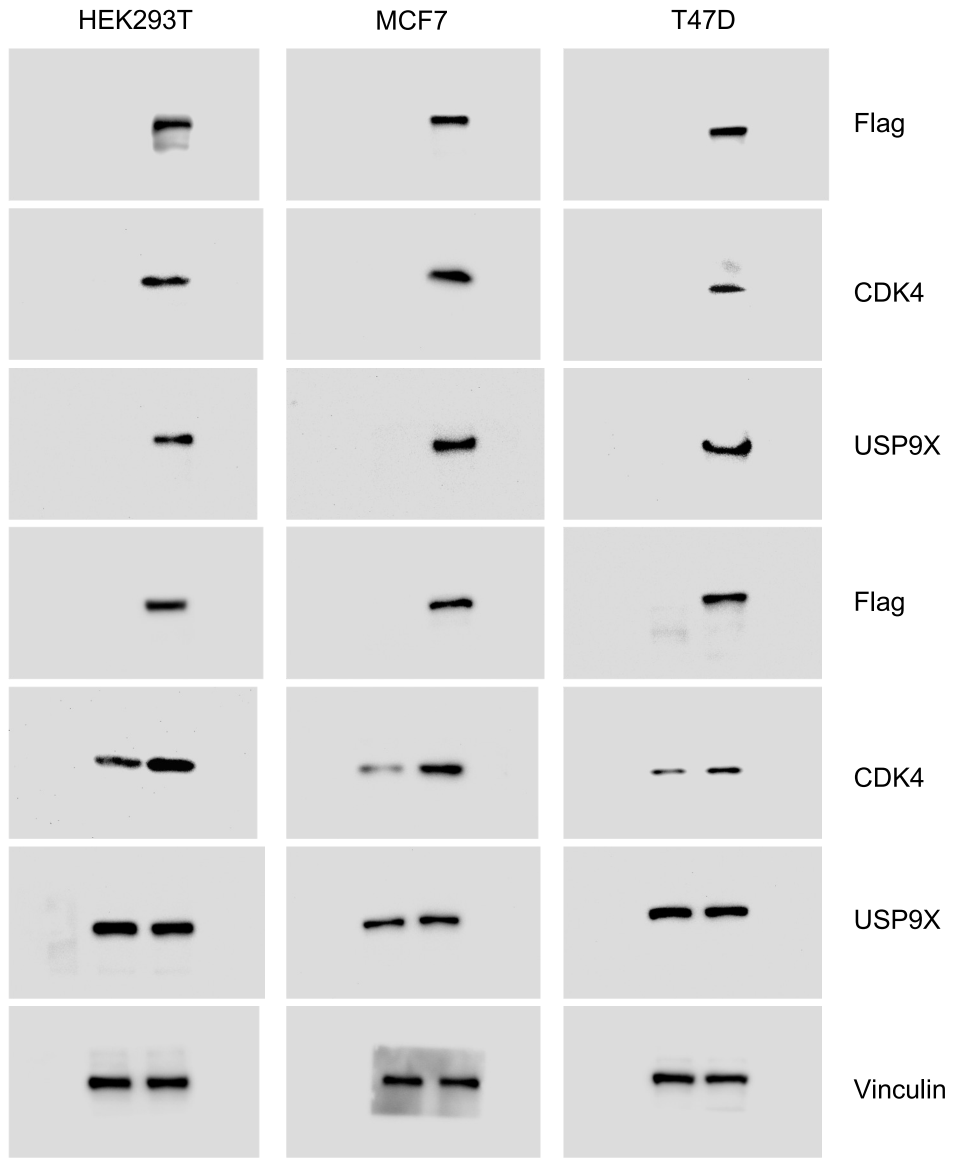

Supplemental Figure 11I and J

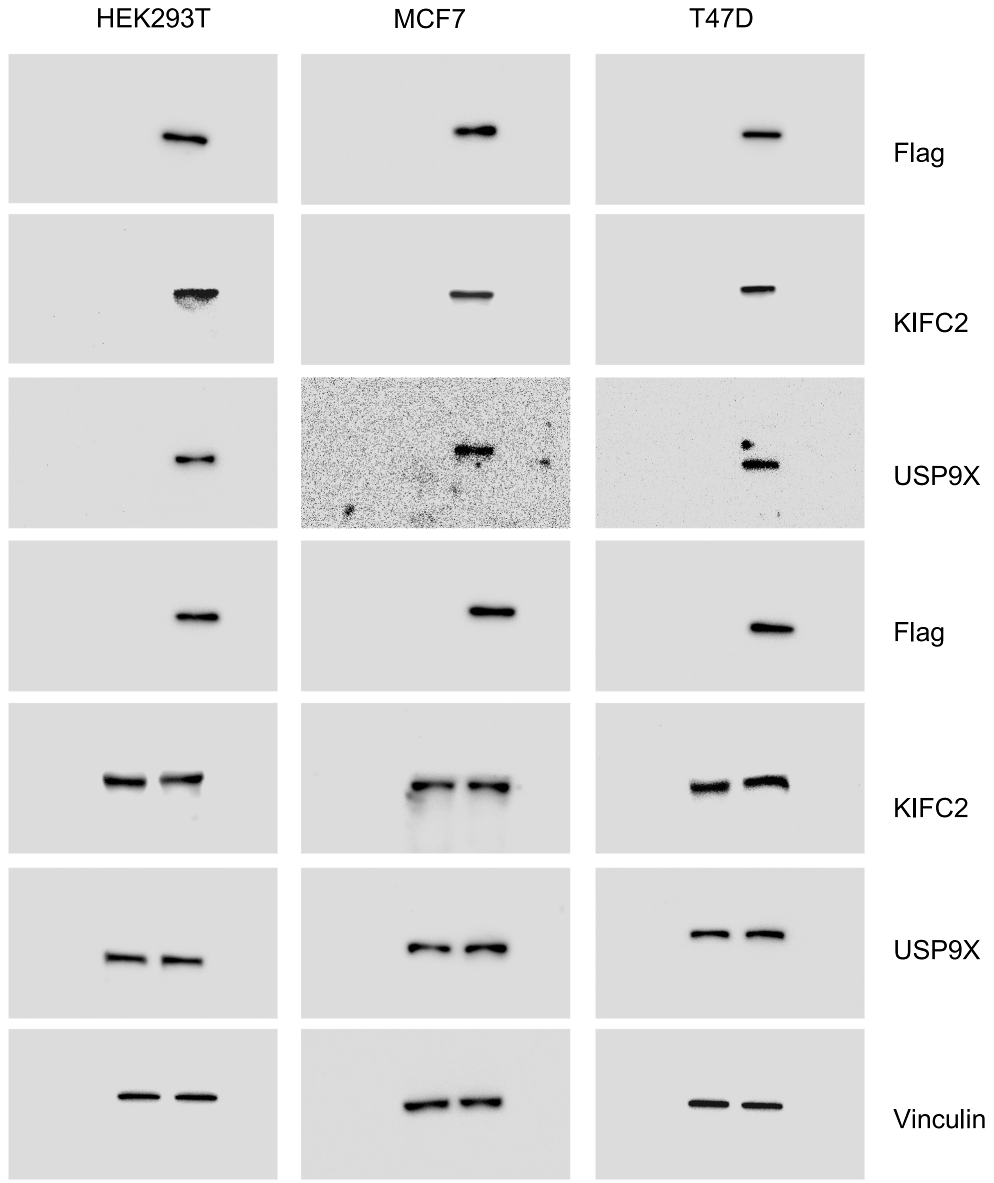

Supplemental Figure 12B

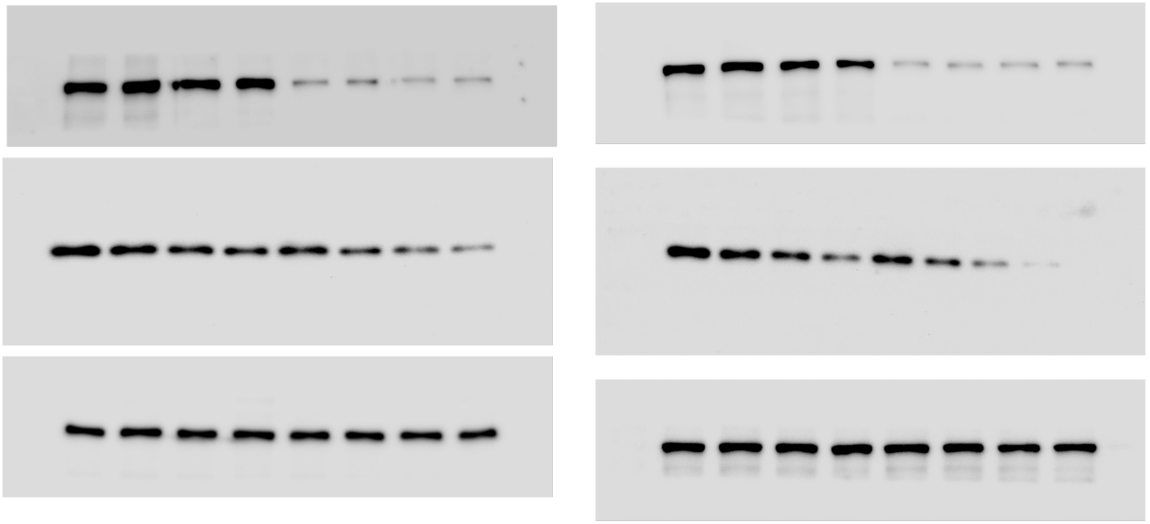

Supplemental Figure 12D

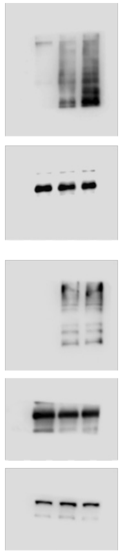

Supplemental Figure 12E

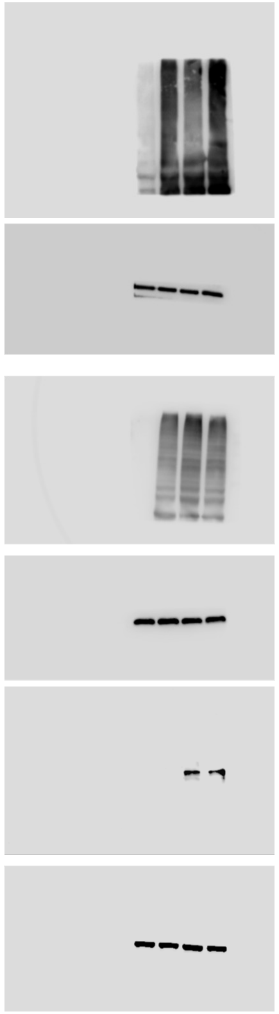

Supplemental Figure 12F

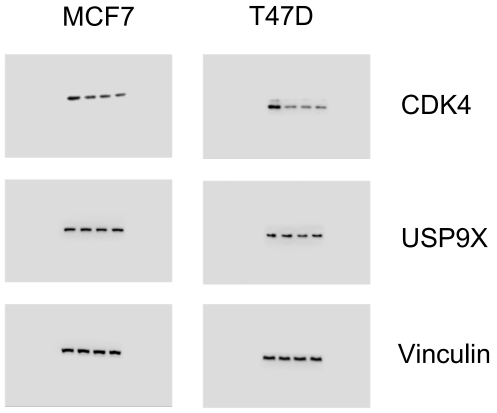

Supplemental Figure 12G

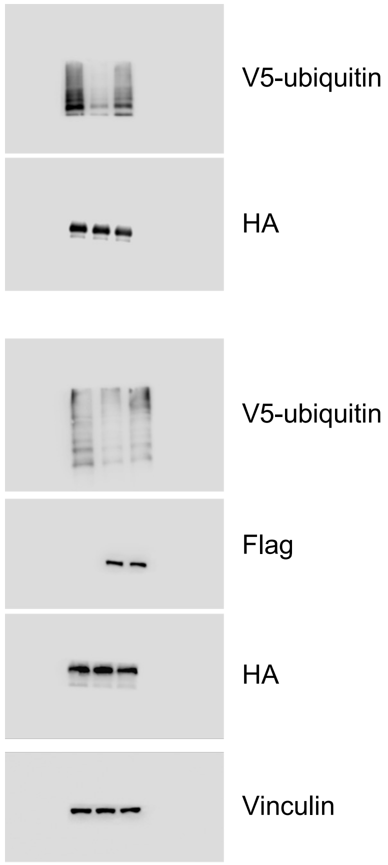

Supplemental Figure 12H

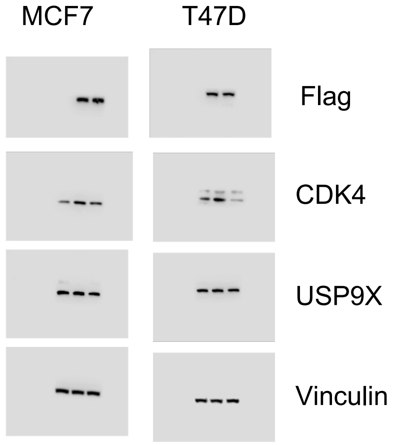

Supplemental Figure 12I

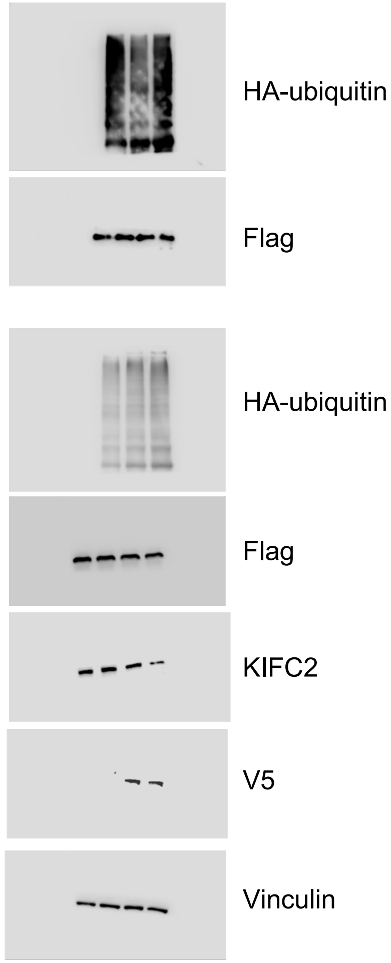

Supplemental Figure 12J

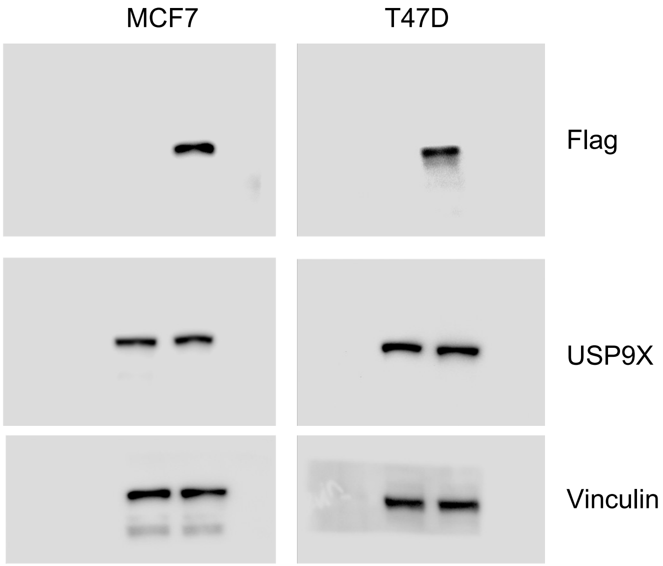

Supplemental Figure 12K

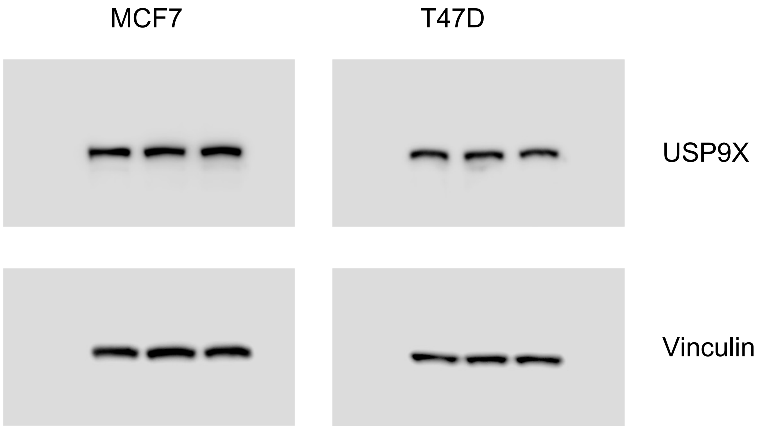

Supplemental Figure 16A

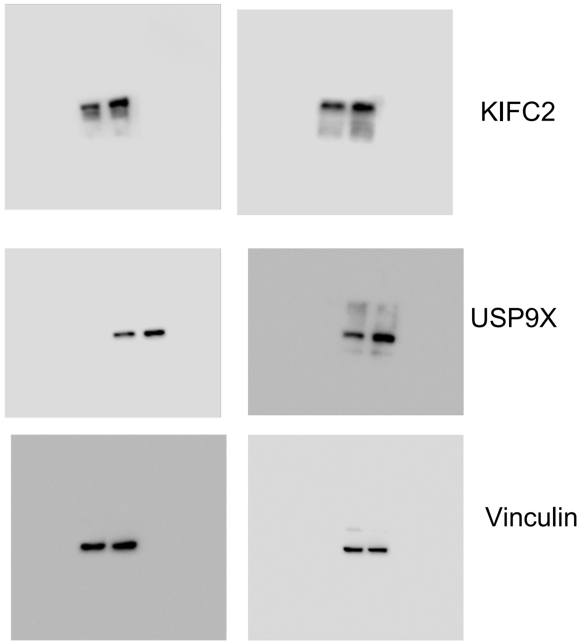

Supplement: Unedited blot and gel images [file jci-135-183531-s139.pdf]
